# Dementia care in the United Arab Emirates: an environmental scan of services, policy, workforce, and caregiving contexts

**DOI:** 10.1186/s12913-026-14598-9

**Published:** 2026-05-26

**Authors:** Seada A. Kassie, Arlene J. Astell

**Affiliations:** 1 University of Reading, Reading, UK; 2 Middlesex University Dubai, Knowledge Park, Dubai, United Arab Emirates; 3 Northumbria University, Newcastle Upon Tyne, UK; 4 University of Toronto, Toronto, Canada

**Keywords:** Dementia care, Environmental scan, United Arab Emirates, Health policy and services, Qualitative description analysis, Workforce preparation, Family caregiving, National dementia strategy

## Abstract

**Background:**

Dementia care in the United Arab Emirates remains poorly documented despite the country’s advanced healthcare infrastructure and rapidly ageing population. The absence of a national dementia strategy, combined with limited local research, leaves policymakers and practitioners without baseline evidence to guide service development. Cultural expectations position families as primary caregivers, yet how families navigate this responsibility has not been systematically documented. This study produced the first comprehensive environmental scan of dementia care in the UAE, mapping services, policies, workforce preparation, and caregiving contexts.

**Methods:**

A two-component environmental scan was conducted. Grey literature sources were systematically identified and analysed, including government policies, health authority documents, service descriptions, and materials from non-governmental organisations; sixty-one sources published between 2014 and 2025 were included. Semi-structured interviews were conducted with twelve healthcare professionals working in dementia care settings. Both components of the data were analysed using a qualitative descriptive approach, with patterns identified inductively and integrated across sources.

**Results:**

Several patterns converged across both components: the absence of dementia-specific policy frameworks, fragmented and uncoordinated services, gaps in workforce training, and the centrality of families in caregiving amid limited formal support. National policies addressed older adult wellbeing without naming dementia, and services operated without clear care pathways. Interviews revealed delayed diagnoses attributed to the perception of cognitive decline as a normal part of ageing, reliance on pharmacological management, and the absence of community-based respite services. Families increasingly relied on domestic helpers and private homecare providers, taking supervisory roles rather than delivering care directly. Professionals were unfamiliar with person-centred care as a concept and lacked formal training in dementia-specific care approaches.

**Conclusions:**

Dementia care in the UAE is characterised by strong health infrastructure but fragmented dementia-specific services, limited workforce preparation, and heavy reliance on families and private care service providers. The absence of a national dementia strategy represents a critical gap. These findings provide the first baseline evidence of the UAE dementia care landscape, offering a foundation for policy development, workforce training, and service design that is responsive to the demographic, cultural, and structural realities of this context.

**Supplementary Information:**

The online version contains supplementary material available at 10.1186/s12913-026-14598-9.

## Introduction

The United Arab Emirates (UAE) presents a distinctive context for dementia care research and policy development. As a high-income state in the Gulf Cooperation Council (GCC) where expatriates constitute approximately 88% of residents [1, 2], the country combines rapid demographic transition with policy ambitions around healthy ageing, wellbeing, and quality of life for older citizens [3]. Yet dementia remains largely absent from national strategies and planning frameworks, addressed only implicitly within broader agendas for care for older people [4, 5]. Family-led care remains the normative model, grounded in cultural and religious values that position filial responsibility as a moral duty [6, 7]. At the same time, increasing reliance on domestic helpers and private home-care agencies reflects the practical pressures facing families navigating dementia care without adequate institutional support [8]. The healthcare system itself is stratified by citizenship, with nationals entitled to comprehensive government-funded services while expatriates depend primarily on employer-sponsored insurance or private provision [9, 10]. This dual structure shapes access to diagnosis, treatment, and long-term care in ways that remain underexplored in the dementia care literature. Existing reviews have highlighted the scarcity of research on dementia in the region, with available studies predominantly focused on prevalence, caregiver burden, and risk factors, while comparatively little attention has been given to service delivery, workforce preparation, or system-level planning [4–6, 11, 12]. This gap is particularly pronounced in the Gulf region, where health systems are evolving rapidly but dementia-specific infrastructure and training pathways remain underdeveloped.

Environmental scanning offers a systematic approach to mapping a domain across multiple evidence sources, including analysis of government and organisational documents, interviews with key stakeholders, and review of published and grey literature to identify current resources, emerging trends, and critical gaps [13–15]. Environmental scans have been applied in dementia care contexts to map primary care models across multiple countries [16], to identify available interventions for older adults with neurocognitive disorders [17], and to assess equity-related workforce measures [18]. However, no environmental scan of dementia care has been conducted in the Gulf region or the broader Middle East. This approach aligns with calls for context-specific evidence to inform dementia care policy and practice in the Gulf region [19, 20]. The present study applies this methodology to examine the dementia care landscape in the UAE through analysis of UAE-specific grey literature sources and interviews with healthcare professionals directly involved in dementia care delivery.

### Study aim and research questions

The aim of this study was to conduct an environmental scan of dementia care in the UAE, mapping services, practices, and barriers and challenges to providing optimal dementia care. The study addressed the following questions: What dementia care services and initiatives exist in the UAE, and how are these framed in policy and public discourse? What are the experiences and perspectives of healthcare professionals providing dementia care? And what gaps and challenges characterise the current landscape? By integrating documentary and experiential sources, the study generates country-specific baseline evidence to inform policy development, workforce training, and future research, while offering insights of relevance to other Gulf states facing similar demographic and cultural dynamics.

## Method

### Study design

This study was designed as a comprehensive environmental scan to examine the dementia care landscape in the UAE. This approach is particularly suited to applied health services research in contexts where formal evidence is sparse or fragmented, and where policy-relevant insights must be drawn from both published and unpublished sources [21].

The environmental scan comprised two components:

1. Grey literature analysis of non-indexed but locally relevant sources on dementia care services and practices in the UAE
2. Interviews with healthcare professionals directly involved in dementia care in the UAE

These two components serve complementary purposes. The grey literature analysis captures the formal landscape of services, policies, and initiatives as documented by government and non-government entities, while the interviews provide experiential perspectives from practitioners navigating that landscape in everyday practice. Together, they offer a comprehensive account of dementia care in the UAE that neither source could provide alone. The study was granted ethics approval by the Dubai Scientific Research Ethics Committee (DSREC) of the Dubai Health Authority (Initial approval letter reference: DSREC-SR-03/2023_06; Annual renewal approval letter reference: DSREC-SR-05/24_08) and the University of Reading Research Ethics Committee (UREC) at the University of Reading, United Kingdom (Ethics approval letter reference: UREC 23/07). Preparatory work for the environmental scan included structuring the extraction templates, refining the search strategy, and arranging the interviews. The interviews with healthcare professionals were conducted between September 2023 and April 2024. The grey literature search was conducted between July and August 2025, with final synthesis and reporting completed between August and September 2025.

### Grey literature analysis

A structured grey literature search was conducted to capture non-indexed but locally critical information on dementia care services and practices in the UAE. Grey literature, including government reports, policy documents, organisational websites, and service descriptions offers essential insights in contexts where peer-reviewed research is limited and where local knowledge is held by institutions rather than documented in academic publications [22–24]. Sources included government portals, local health authority websites, private care service and training providers, and Non-Governmental Organizations (NGOs) active in dementia care policies, community initiatives, and service delivery.

### Search scope and strategy

The search targeted UAE entities including the UAE Government Portal [25], Ministry of Health and Prevention [26], Dubai Health Authority [27], Department of Health Abu Dhabi [28], Emirates Health Services [29, 30], Sharjah Social Services Department [31], the Al Qasimi Foundation, and local non-governmental organisations (e.g., 4get-me-not [32]). Manual browsing, Google searches, and snowballing technique were employed to ensure comprehensive coverage of relevant sources. The specific search terms used to identify relevant grey literature sources are documented in Supplementary File 1.

### Data extraction and synthesis

Information from each included grey literature source was extracted into a structured spreadsheet (see Supplementary File 2), recording source category, source name, focus area, publication date, type of service/initiative, specific dementia services, target population, key features/description, cultural/religious/family considerations, and dementia care service gaps. The extraction process captured both descriptive details and interpretive elements, such as information on how dementia is framed in policy and public discourse. Metrics such as length of operation, number of service users, and audience reach were not consistently documented across the identified grey literature sources, and in most cases were unavailable. This opacity is itself reflective of the limited formalisation of the dementia care landscape in the UAE.

The extracted data were synthesised using a qualitative descriptive approach [33–35], with patterns, contrasts, and omissions identified inductively across sources. Recurring threads included policy visibility, service fragmentation, family centrality, and limited dementia-specific training for formal and informal caregivers. Given the heterogeneous nature of grey literature, the synthesis was exploratory and aimed at mapping breadth rather than judging quality, while acknowledging challenges in consistency of reporting and continuity of services and initiatives [22, 23].

### Interviews with healthcare professionals

Semi-structured individual and focus group interviews (See Supplementary File 3 for interview transcripts) were conducted with healthcare professionals involved in dementia care delivery in the UAE, providing experiential accounts to complement the documentary evidence from the grey literature. Semi-structured interviews employ a guide of pre-determined questions while allowing flexibility for follow-up prompts and exploration of emergent topics [36]. The interview guide for this study was developed through a structured process aligned with the study’s research questions and reviewed and refined in consultation with the supervisory team prior to data collection. The formal grey literature search was conducted as a subsequent and independent component of the environmental scan, following the completion of the interviews.

### Sampling and recruitment

Using purposive sampling, which is the deliberate selection of participants based on their capacity to provide information-rich accounts relevant to the phenomenon under study [37], four specialist and consultant geriatricians were recruited for the individual interviews, as well as two gender-specific focus groups consisting of nurses and assistant nurses - in line with cultural norms. Participants were selected based on their direct professional involvement in dementia care delivery in the UAE, ensuring representation across the range of clinical roles (geriatricians, nurses, assistant nurses) and care settings (government hospital, long-term care facility) in which dementia care is provided.

### Data collection

Conducted between September 2023 and April 2024, the individual interviews were held in person or online (Microsoft Teams) depending on the availability of the participants, whereas both focus group interviews were conducted in person. All interviews were led by the first author, audio-recorded with consent, and transcribed verbatim.

### Data analysis

The interview data were analysed using a qualitative descriptive approach [33, 34], which prioritises comprehensive summary and low-inference interpretation over deep thematic abstraction. This approach is well-suited to applied health services research where the aim is to describe phenomena as experienced by participants and to identify patterns relevant to practice and policy [35, 38]. The analysis involved repeated and close engagement with the transcripts: each transcript was read in full multiple times before coding commenced, and initial codes were generated inductively from the data using Quirkos (version 2.5.3), then reviewed and refined through subsequent passes of the data. This iterative process refers specifically to the analytic engagement with the transcripts and did not involve modification of data collection in response to emerging analytical categories, as analysis commenced following the completion of all interviews. Codes were grouped into descriptive categories reflecting recurring topics across participants, including service fragmentation, workforce constraints, family expectations, and training gaps. Attention was paid to both consistencies and tensions within the data, and findings are presented as narrative summaries that synthesise perspectives across participants rather than foregrounding individual voices. Illustrative quotations are used selectively to anchor key points.

### Integration across datasets

Across the grey literature analysis and the interview data, synthesis followed the same principles: systematic extraction, inductive categorisation, and descriptive pattern identification. This allowed findings from policy documents, service descriptions, and practitioner perspectives to be examined together while preserving the distinctiveness of each source type. The integration was iterative, moving between datasets to identify convergences, divergences, and gaps. The process was exploratory and descriptive [33–35], aimed at mapping the current dementia care landscape in the UAE.

### Researcher positionality and trustworthiness

Transparency about the researcher’s positioning in relation to the study context is important for evaluating the trustworthiness of qualitative findings [39, 40]. The first author is a doctoral researcher at the University of Reading, U.K. and Senior Lecturer in Psychology at Middlesex University Dubai, UAE with over a decade of academic experience in the UAE, during which they have developed professional relationships with healthcare providers and facilities involved in dementia care. This insider familiarity facilitated access and rapport during recruitment and data collection but also required critical reflexivity to ensure that prior assumptions did not constrain the analytical process. Prior to this doctoral research, the first author worked in clinical research at a private neuropsychiatric clinic, gaining direct experience with populations living with neurological and neurodegenerative conditions, including Alzheimer’s disease and dementia. The first author had no prior professional connection to the specific healthcare settings in which the interviews were conducted. To support trustworthiness, the first and second authors met weekly via Microsoft Teams throughout the data collection and analysis phases. These meetings provided a regular forum for reviewing emerging analytical decisions, discussing interpretive choices, and monitoring consistency in the analytical process.

Data collection and analysis were conducted by the first author as part of a doctoral research programme. As the doctoral supervisor, the second author oversaw the study design, reviewed and refined the interview guide, monitored data collection procedures, and independently reviewed the analytical categories and interpretive decisions at each stage of the analysis. While the single-coder approach is a recognised limitation of the study, it was consistent with the structure of doctoral research and was mitigated through sustained supervisory engagement, iterative discussion of emerging categories, and transparent documentation of analytical decisions throughout.

## Results

The results from the environmental scan are presented across the two components of the study. First, analysis of grey literature focusing on UAE-specific policies, services, initiatives, and emerging practices is presented. All sources were reviewed and coded as part of the iterative analysis; only representative examples are cited to illustrate each pattern, but the synthesis draws on the full dataset. Some sources that duplicated information or provided only tangential detail are not cited individually in order to avoid redundancy, though their content contributed to the patterns reported. Second, the interviews with healthcare professionals offer experiential insights into diagnosis and delivering everyday care, training and education, and barriers and challenges.

Each component was analysed using a qualitative descriptive approach [33–35], with descriptive categories generated inductively from the data and refined through repeated engagement. The purpose was not to produce rigidly separate categories, but to identify recurring patterns that illustrate how dementia care is positioned across policy and practice discourse and lived professional experience. Together, these components capture both the formal landscape of dementia care in the UAE, as documented by government and non-government entities, and the realities of practice as experienced by those delivering care.

### Grey literature analysis

A total of 61 sources were extracted and reviewed, spanning government policies and strategies, public and private healthcare service providers, news releases, academic contributions, training services, and community initiatives. Table 1 provides a condensed summary of findings from the 61 grey literature sources organised by source category. Detailed descriptions of the individual sources are provided in Supplementary File 2. Sources were read iteratively, with initial codes generated inductively. Codes were then grouped into descriptive categories reflecting recurring patterns across sources. The narrative that follows presents findings organised around seven cross-cutting patterns identified through this process and discussed in the narrative below.

**Table 1:** Summary of grey literature findings on dementia care in the UAE, organised by source category

Source Category	Sources	Key Findings	Gaps and Challenges Noted
National Healthcare Policies, Strategies, and Plans	[25, 41–45]	Policies emphasise dignity, protection, inclusion, and improved quality of life for senior Emiratis across physical, psychological, and social domains; healthcare strategies address universal access, mental health, and healthy ageing	Dementia not named as a distinct priority in any federal policy; no dementia-specific provisions, implementation mechanisms, or operational guidance; geriatric focus indirect
Government Foundation Campaigns and Evaluations	[46, 47]	One of the few initiatives integrating awareness, research, clinical training, and home-based care; 2014 system review explicitly called for an integrated dementia care system in the UAE	Fragmented care, lack of coordination across services, limited public awareness, and workforce gaps identified as early as 2014; unclear whether activities are ongoing
Government Health Authority News Releases	[26, 27, 29, 30, 48–52]	Alignment with WHO healthy ageing framework; launch of new memory clinics and screening initiatives; EHS comprehensive senior care model covering 14 integrated initiatives	Dementia rarely named explicitly in announcements; general elderly care described without dementia-specific pathways; no detail on long-term follow-up or community care integration
Government Healthcare Service Providers	[28, 31, 48–51, 53, 54]	Dedicated elderly care centres offering geriatric, rehabilitation, and in some cases memory care services; multidisciplinary teams in some facilities; home visit programmes documented	Services predominantly target senior Emiratis; access for expatriates unclear or unavailable; no dementia-specific protocols or integration with community or home care documented across most providers
Academic and Research Sources	[55–58]	Growing scholarly engagement with UAE dementia care; documentation of fragmented services and dominance of private providers; identification of inadequate culturally and linguistically appropriate diagnostic tools for Arabic-speaking populations	Calls for national dementia strategy, workforce training, and culturally appropriate services remain unaddressed; potential misdiagnosis or underdiagnosis due to absence of validated Arabic-language assessment tools
Local Media Sources	[59–72]	Personal narratives of family caregiving highlighting emotional toll and reliance on memory clinics and support organisations; projected 1,795% increase in UAE dementia cases by 2050	Limited awareness and stigma remain barriers to early diagnosis; lack of national-level screening programmes; family-based care systems acknowledged as potentially unsustainable without formal support
Private Home Healthcare Providers	[57]	Home-based dementia care services targeting expatriate families; over 80 licensed home care agencies nationally as of 2020; acknowledgement of gaps in government support for expatriates	Limited government support for expatriates in long-term dementia care; unclear pathways for accessing affordable specialised services; home-based dementia care often not covered under standard insurance plans^87^
Private Hospitals and Clinics	[69, 73–86]	Diagnostic services, neuropsychological assessments, and treatment plans available across multiple private providers; descriptions of multidisciplinary teams and personalised care; standard UAE insurance plans typically exclude dementia care alongside certain psychiatric conditions^87^	No coordination with community or home-based care documented; caregiver training and integration with national planning absent; services vary in scope and quality with no standardisation across providers
Community and NGO Initiatives	[32, 87]	Workshops, public campaigns, and awareness activities during globally observed dementia awareness periods; member organisation of Alzheimer’s Disease International	No official national recognition or integration into government dementia care strategy; activities episodic and tied to specific events; reach and sustainability inconsistently documented
Private Caregiver Training Providers	[88–90]	Caregiver certification courses covering dementia care basics, daily living support, safety, mobility, hygiene, and nutrition; foundational training available to general public and aspiring caregivers	No standardisation across providers; no cultural or religious tailoring documented; no formal integration into national training pathways; no evidence of standardisation across providers, cultural tailoring, or formal integration into national training pathways.

*Note: Full source-by-source details are provided in Supplementary File 2*

### Dementia absent from explicit policy recognition

The absence of dementia from national policy frameworks is documented in the peer-reviewed literature [4, 5, 12], but the grey literature analysis establishes which specific documents were examined and confirms that the gap is consistent across federal, emirate, and health authority levels [25, 26, 41–44, 46]. The National Policy for Senior Emiratis [41, 42] establishes rights to medical care, social participation, dignity, and family support, with emphasis on intergenerational solidarity and Islamic values, but contains no dementia-specific provisions or implementation mechanisms. The National Strategy for Wellbeing 2031 [43] sets out fourteen components and nine strategic objectives covering mental health, active lifestyle, and social cohesion, without reference to dementia or dementia-related indicators. Strategic health plans including UAE Vision 2021 and subsequent national agendas [25] address universal healthcare access and healthy ageing without identifying dementia care as a specific service area. Health authority announcements from the Ministry of Health and Prevention [26] and Emirates Health Services [29] describe alignment with WHO’s healthy ageing framework and early detection priorities, but dementia remains embedded implicitly within these broader agendas rather than named as a target condition. The Department of Community Development Abu Dhabi [45] similarly upholds values of dignity and family cohesion for senior citizens without dementia-specific guidance. Although these policies set positive aspirations for older adult wellbeing, they do not establish dementia as a health priority requiring dedicated resources, services, or planning. Across all six sources, dementia-specific provisions, implementation mechanisms, and operational guidance were consistently absent. The grey literature analysis adds to the existing literature by providing ground-level documentary evidence of the policy gap across the sources identified and reviewed. Review of the policy documents and government portal sources included in the search revealed that each addresses older citizen wellbeing without naming dementia as a distinct priority, and that dementia-specific provisions, implementation mechanisms, and operational guidance were consistently absent across the sources examined.

### Family positioned as the default caregiving unit

The positioning of the family as the primary unit of dementia care is documented across government policies, academic sources, media narratives, and hospital documentation in the grey literature - a convergence that the peer-reviewed literature anticipates but does not establish at the level of specific local sources. National documents affirm family responsibility in caregiving and uphold values of dignity, protection, and intergenerational cohesion [3, 42, 45]. Academic commentaries identify the family as the default caregiving unit, noting the limited availability of institutional alternatives [55, 56]. Media narratives across fourteen sources reinforce this expectation, portraying family caregiving as a cultural duty rooted in Islamic values and Emirati traditions, while also acknowledging the strain and stigma it imposes [60–68]. Hospital and clinical sources similarly describe family involvement as central to the care process [69, 70]. Religious and cultural norms are woven into wellbeing strategies, with initiatives promoting community engagement and family-centred approaches. While this cultural framing is presented as a strength reflecting values of respect, cohesion, and filial duty, it simultaneously exposes systemic vulnerabilities. When families are positioned as the default caregivers without access to training, respite, or coordinated support, the caregiving burden falls disproportionately on informal networks. Several sources noted caregiver stress, emotional toll, and the unsustainability of family-based systems without formal support structures [62, 63, 69]. What the grey literature contributes here is evidence of how pervasively and uncritically this framing operates across source types that serve different purposes and audiences: government portals affirm it as policy, clinical sources assume it as operational reality, and media sources celebrate it as cultural duty while simultaneously documenting its human costs. No source identified a structural mechanism to address that burden, pointing to a gap that policy discourse consistently acknowledges but has not acted to address.

### Services fragmented across public and private sectors

The grey literature analysis identified a substantial and varied service landscape across government hospitals, private clinics, and home-care agencies, yet no source described coordination between these providers or documented care pathways connecting diagnosis to long-term support. Government health authorities have announced new memory clinics and frameworks for healthy ageing [26, 48, 49], and media sources have highlighted specialised geriatric facilities [30, 50, 51]. Thirteen private hospitals and clinics describe diagnostic services, neuropsychological assessments, and treatment plans for dementia [69, 71–86], with many referring to multidisciplinary teams and personalised care. Yet across these thirteen sources, none documented coordination with community or home-based care, caregiver training provision, or integration with national planning. The Salama bint Hamdan Al Nahyan Foundation’s 2014 review [46] identified this fragmentation explicitly, calling for coordinated services, trained staff, and improved public awareness. Academic sources reached the same conclusion [47, 55, 56]. What the grey literature analysis adds to these existing observations is a current and specific account of which services exist and what each documents about its own provision, establishing that the fragmentation identified a decade ago persists and that the service landscape, while expanded, remains without a coordinating framework.

### Private sector expanding without national integration

The grey literature documents a substantial and growing private sector response to unmet dementia care needs, operating independently of national planning. As of 2020, more than 80 home-care agencies were licensed nationally, with approximately 50 actively operating [91] - a scale of private provision not documented in the peer-reviewed literature. Enayati Home Healthcare [57] explicitly described filling gaps in dementia care for expatriate families who lack extended family networks and cannot access government-funded services, noting unclear pathways for expatriates seeking affordable specialised care. Three private training providers offer caregiver certification courses covering dementia care basics [88–90], yet review of their publicly available materials revealed no evidence of standardisation across providers, cultural tailoring, or formal integration into national training pathways. The insurance landscape compounds these gaps: standard UAE insurance plans typically exclude dementia care alongside certain psychiatric conditions [91, 92], meaning that the private sector on which many families depend is itself inaccessible or financially prohibitive for a substantial portion of the population. Taken together, these findings document a private sector that has expanded to meet a need that public infrastructure has not addressed, but that operates without oversight, standardisation, or coordination with national planning.

### Community and academic initiatives episodic and small-scale

The community and academic activity documented in this section operates at a different level of the dementia care system than the policy frameworks described in the preceding patterns. The absence of dementia from national policy does not preclude activity by civil society organisations, academic institutions, and awareness campaigns; in contexts where formal policy has not yet responded to an emerging public health need, such actors often function as early movers. Their co-existence with policy silence is itself analytically significant, reflecting bottom-up momentum in the absence of top-down coordination.

Beyond formal policy and healthcare service provision, the grey literature identified a small number of community organisations, academic institutions, and awareness initiatives engaged with dementia in the UAE. The 4get-me-not Alzheimer’s Organization [32], the only UAE-based member of Alzheimer’s Disease International identified in the search, offers workshops, training, and public campaigns but has no documented official recognition or integration into government strategy. A dementia awareness event held by UAE University during Ramadan in 2017 [87] demonstrated cultural sensitivity in its timing but was documented as a one-off event with no recorded follow-up. The Salama bint Hamdan Al Nahyan Foundation [46, 47] represents the most integrated non-governmental effort identified, combining awareness, research, clinical training, and home-based care, though its health programme webpage has not been updated since 2017 and it remains unclear whether these activities are ongoing. Academic contributions from Khalifa University [58], the Oxford Institute of Population Ageing [55], and Tse et al. [56] signal growing scholarly engagement with UAE dementia care, but these remain individual outputs rather than part of a coordinated research agenda. Media and awareness sources documented projections of a 1,795% increase in UAE dementia cases by 2050 [52, 93] and called for urgent investment in care infrastructure, but without documented policy response. Across these sources, the grey literature establishes that the specific actors operating in this space are few, loosely connected, and working without a national framework within which to situate or coordinate their contributions.

### Access stratified by citizenship status

The grey literature provides specific documentary evidence of citizenship-based stratification in dementia care access, moving beyond the structural observation in the peer-reviewed literature to name the facilities and entitlements involved. Government portal sources consistently specify ‘Senior Emiratis’ as the target population for dedicated services [3, 45], and two specialised elderly care centres, Al Mamzar Community Centre for Elderly and the Dubai Health Seniors’ Happiness Centre [53, 54], are documented as serving older citizens with unclear or no access for expatriates. These are among the most comprehensively described dementia-relevant facilities in the grey literature, yet their documented beneficiary group excludes the majority of the UAE population. The practical consequences of this exclusion are documented from the private sector side: Enayati Home Healthcare [57] explicitly describes filling gaps in dementia care for expatriate families, noting limited government support, unclear access pathways, and dependency on private agencies as defining features of the expatriate experience. This account, drawn from a private provider rather than a policy document, offers ground-level evidence of what citizenship-based stratification means in practice for the majority of the UAE population seeking dementia care: a daily navigational challenge shaped by unclear pathways, limited government support, and dependency on private provision.

### Workforce preparation limited and unstandardised

The grey literature provides a specific account of the workforce training landscape rather than simply confirming that gaps exist. Three private training providers were identified: NLP Tech Training Centre, Samson Training Center, and UAE Montessori [88–90], each offering caregiver certification courses covering dementia care basics, daily living support, safety, mobility, hygiene, and nutrition. Review of their publicly available materials revealed no evidence of standardisation across providers, cultural or linguistic tailoring, or formal integration into national training pathways. Across the thirteen private hospital and clinic sources reviewed, caregiver training and workforce development were rarely mentioned; providers documented services for patients but not preparation of the staff delivering them. The Salama bint Hamdan Al Nahyan Foundation [47] supported dementia-related research and public awareness initiatives, though its health programme webpage has not been updated since 2017, leaving the continuity of these activities undocumented. Khalifa University [58] identified a specific and consequential training gap: the absence of culturally and linguistically appropriate diagnostic tools for Arabic-speaking populations, with documented potential for misdiagnosis or underdiagnosis. This finding is notable because it locates the workforce preparation deficit not only in caregiving practice but in the diagnostic process itself, suggesting that the consequences of inadequate training extend to whether dementia is correctly diagnosed in the first place. Taken together, the grey literature establishes not just the known gap that workforce preparation is limited, but also the precise nature of that limitation in terms of what training exists, what it covers, what it omits, and where the consequences are most clinically significant.

### Summary

Collectively, the grey literature depicts a dementia care environment that is evolving but underdeveloped. National strategies embed dementia within broader ageing agendas without establishing it as a distinct health priority. Services exist in pockets through government hospitals, memory clinics, and private providers, but remain fragmented and lack coordination. Cultural and religious values position families as the primary caregivers, a framing that reflects genuine cultural strengths but simultaneously exposes systemic vulnerabilities when families lack support. The private sector is expanding to meet demand but operates independently of national planning. Community and academic initiatives contribute through awareness and research, but their efforts are episodic and unintegrated. Access to services differs by citizenship status, with expatriates largely dependent on private provision. Workforce preparation remains limited and unstandardised. Across all source types, these gaps were consistently noted: the absence of a national dementia strategy, insufficient dementia-specific services, limited workforce preparation, and inadequate support for family caregivers.

To explore how these systemic patterns are experienced in practice, the next section presents findings from interviews with healthcare professionals working directly within the dementia care environment in the UAE.

### Interviews with healthcare professionals

While the grey literature analysis captures the formal landscape of policies, services, and initiatives, the interviews explored how dementia care is experienced by healthcare professionals working within the public healthcare sector. Table 2 provides an overview of participants’ personal and professional characteristics and the original interview transcripts are presented in anonymised form in Supplementary File 3. Inclusion criteria were: (1) currently working in a healthcare setting in the UAE, and (2) directly involved in the care of older people living with dementia. Years of professional experience, postgraduate qualifications, and advanced training in dementia care were not inclusion criteria.

**Table 2:** Demographic characteristics of interview participants

Participant ID	Gender	Professional Role	Years of Experience
G1	Male	Specialist Geriatrics	>15 years
G2	Male	Consultant Geriatrics	>27 years
G3	Female	Consultant Geriatrics	>25 years
G4	Male	Senior Specialist Geriatrics	>13 years
RN1	Female	Registered Nurse	>17 years
RN2	Female	Registered Nurse	>20 years
RN3	Female	Registered Nurse	>12 years
RN4	Male	Registered Nurse	>15 years
AN1	Female	Assistant Nurse	>10 years
AN2	Male	Assistant Nurse	>10 years
AN3	Male	Assistant Nurse	>20 years
AN4	Male	Assistant Nurse	>18 years

*Note; Years of experience reflects overall professional tenure and was not an inclusion criterion for participation*

Transcripts were read iteratively, with initial codes generated inductively from the data. Codes were grouped into descriptive categories reflecting recurring patterns across participants’ accounts. The analysis involved comparing across participants’ accounts to identify similarities and differences in their experiences and perspectives. Findings are presented as narrative summaries that synthesise perspectives across participants, with illustrative quotations used selectively to anchor key points. Four descriptive categories were identified: (1) local research and prevalence data, (2) diagnostic pathways and management practices, (3) workforce preparation and training, and (4) day-to-day care practices and challenges.

### Local research and prevalence data

Participants described dementia as substantially more prevalent than official figures suggest. Physicians estimated that between 40 and 75% of patients they encountered presented with some form of cognitive impairment, though these figures were based on clinical experience rather than documented data. Cognitive issues were often identified incidentally during assessments for falls or fractures rather than through targeted screening. The absence of a health registry was consistently noted as a barrier to establishing prevalence: “we cannot find out the exact percentage of dementia” (G3). The practical obstacles were substantial: no research associates, no protected scholarly time, and a large volume of patient records to work through independently. As one physician summarised: “All of this is on the investigators and out of our clinical time … what is normally meant to be done in six months takes a long time. It’s exhaustive. It’s cumbersome.” (G1) There was recognition among the physician participants that locally derived evidence was necessary, but competing clinical demands consistently precluded it: “no time … our academic life was a long time ago.” (G2). Gendered patterns in long-term care admissions were also noted. Female patients were described as more likely to remain at home with extended family, even in the absence of immediate children. One physician observed that “women are protected in the community” and that families were reluctant to admit women to institutional care (G3). This pattern has direct implications for the data gap described above: if female patients are systematically less likely to appear in institutional care settings, prevalence estimates derived from those settings will underrepresent women, compounding the existing limitations of the available epidemiological data. The prevalence and research data findings connect directly to the grey literature’s documentation of absent epidemiological infrastructure. Academic and media sources identified the lack of local data as a significant barrier to dementia care development, but the interviews add the practitioner perspective: the data gap is not merely a research priority but a lived constraint that clinicians navigate daily, estimating prevalence from clinical experience in the absence of the registry and research infrastructure that would make systematic documentation possible. Without local data, clinical practice defaults to global standards that may not reflect the demographic, cultural, and structural realities of the UAE context.

### Diagnostic pathways and management practices

Diagnosis was described as typically occurring late in the disease trajectory, with late presentation attributed to two compounding factors: the cultural normalisation of cognitive decline as part of ageing, and the availability of domestic and social support structures that compensate for early functional deficits. On the first point, G2 noted that families consider memory problems “a normal part of aging” and do not seek medical attention early. On the second, G4 offered a detailed account of the mechanism:

“If an elderly person lives alone, and he starts to have a problem with his memory, this will be easy to catch so [he] will find difficulty to go to places, he will find difficulty to handle his money, and he will try to find the … the problem [be]cause he is the one who is taking care of himself. That’s why he will take attention. While if the patient is having or surrounded by all these kinds of support as I mentioned … okay … you don’t need to handle the finance, you don’t need to remember the places, a driver will take you to wherever you need … this will make it to later on to discover that you really have a problem or you may ask for help.” (G4)

The consequence is that by the time families seek clinical evaluation, the disease has typically progressed well beyond the mild stage. As G2 described: “Because mostly they consider it as part of aging. They don’t come to us. Usually they come to us when there is behavioural struggle, or extreme cognitive deterioration, or they start to get lost with the car or they go out and they are not able to come back home. They get lost at this stage. Usually we are not talking about early Alzheimer’s.”

Even when families do recognise that something is wrong, navigation of the care system presents its own barrier. As G3 observed: “people in the community are still not sure where to go and whom to contact in case they are noticing problem with cognitive function.” (G3) Compounding this navigational uncertainty, awareness of dementia services was limited more broadly: the term “dementia” was unfamiliar to many families, while “Alzheimer’s” was more widely recognised, and awareness of a diagnosis could itself trigger stigma, with some families reluctant to seek formal confirmation of what they already suspected (G1; G3).

Neuropsychologists and clinical psychologists are not typically available in government facilities. Participants indicated that diagnosis was nonetheless achievable through clinical assessment by geriatricians or neurologists: “We don’t have a neuropsychologist, but we can diagnose dementia without a neuropsychologist” (G3). The absence of clinical psychologists nonetheless had significant consequences for post-diagnostic care. As G1 explained: “Unfortunately, we don’t have other … other auxiliary methods like we don’t have a clinical psychologist on board. We don’t have other support personnel, so our options here are we will directly start off with medications to aim, you know, prevent further degeneration of the condition.” (G1) The phrase “our options here are” reflects systemic constraint: non-pharmacological management was made unavailable by the absence of the personnel needed to deliver it. The same physician identified the psychosocial gap in broader terms, observing that depression rooted in isolation and loneliness was “a very strong condition” among older patients, one that medication addresses inadequately and that dedicated psychosocial services would be better placed to manage. The reliance on pharmacological management documented in this section therefore reflects a workforce configuration in which the clinical psychology infrastructure necessary to support non-pharmacological care does not exist.

Management of behavioural changes associated with dementia relied heavily on antipsychotic medication. Physicians estimated that 60 to 70% of patients exhibiting agitation or aggression received antipsychotics (G1). A stepwise approach was described: physical causes such as infection, pain, or constipation were ruled out first, followed by non-pharmacological strategies, with medication used when these approaches proved insufficient. However, antipsychotic use was also described as serving a dual purpose, managing patient symptoms and alleviating family caregiver burden. G2 articulated this explicitly in the context of advanced-stage dementia: “At this point of the disease, the family maybe is the number one target for me because the patient is no longer aware about anything, is becoming just a source of annoyance for the family. So here I consider the family even if I give him a dose of antipsychotics, he will not suffer anything but at least to relieve the family.” (G2) This account makes clear that prescribing decisions at the advanced stage were shaped not only by patient-level clinical presentation but by the burden carried by those surrounding the patient, a pattern with direct implications for how workforce training and community support services are designed.

Family involvement in care was consistently described as central, reflecting cultural values of filial responsibility. Physicians rated family dedication highly and noted that government leave provisions facilitated caregiving. At the same time, an increasing reliance on domestic helpers and private home-care agencies was evident, particularly among Emirati families. One physician estimated that 95% of Emirati patients had care provided through nursing agencies, with family members supervising rather than providing direct care (G1). This pattern represents a meaningful shift in the caregiving role: the cultural expectation of family care persists, but its practical expression has moved from hands-on delivery to oversight and coordination of contracted staff. The shift is enabled partly by financial capacity and partly by insurance arrangements that, as G2 observed, do not consistently cover dementia caregiving: “Most of the cases of dementia are not without these issues. They need caregiver and nursing but insurance does not support covering caregivers. So what they often do is bring domestic helpers on their own visa from India, from the Philippines, or they make shifts between the daughters.” (G2) For non-Emirati families, this delegation has not occurred to the same extent. Spouses and children remained the primary hands-on caregivers, navigating the same cultural expectation of family responsibility but without equivalent financial resources or access to government support, a distinction that maps directly onto the stratified access patterns documented in the grey literature.

Language barriers were described as manageable in government facilities due to mixed staffing. Care teams included Arabic-speaking staff who assisted with communication, and this arrangement was described as effective: “keeping one of the [Arabic-speaking] staff is the magic technique” (G4). Occasional challenges with patients who spoke only Arabic were acknowledged, but these were addressed through team collaboration.

The diagnostic pathway findings collectively reflect the practice-level consequences of the systemic conditions documented in the grey literature. Late presentation, unclear referral routes, and reliance on pharmacological management in the absence of psychology infrastructure are predictable outcomes of a care environment in which dementia has no dedicated policy framework, services operate without coordination, and workforce configurations do not include the personnel needed to support non-pharmacological care. Where the grey literature identified these gaps at the system level, the interviews show what they mean for the people navigating them: families who do not know which specialist to consult, clinicians whose options have been constrained before any individual clinical decision is made, and a diagnostic process that begins, for many patients, only when the disease has become unmanageable at home. Without a national dementia strategy to establish accountability and coordination, the practice-level failures documented here are unlikely to be addressed at the level at which they originate.

### Workforce preparation and training

Dementia-specific training was described as limited across the care team, with a clear differential between physician and nursing staff preparation. Physicians reported receiving dementia exposure as part of their residency programmes, including rotations with inpatients and home visits to patients with cognitive impairment, though this was embedded within general geriatric education rather than constituting specialised dementia care preparation. For nursing and assistant nursing staff, the picture was starker. When asked directly whether dementia was covered as part of nursing training, RN2 responded: “No.” When asked whether dementia-specific training was provided on commencing employment, the same participant confirmed: “N … no actually.” (RN2) G2 summarised the situation plainly: “Nothing special for nurses. Routine stuff.” (G2)

In the absence of formal dementia training, learning occurred informally and experientially. G4 described how nursing staff acquired dementia-related knowledge in practice: “Most probably we are advising the nurses during our rounds about any questions they need … we do give them lectures in a regular way so it’s not exactly assigned, but it is coming with experience and with the knowledge that we share … but as far as we understand it is not part of their mandatory [training].” (G4) This account is significant: it describes a workforce development model built on incidental knowledge transfer rather than structured competency development, in which what staff know about dementia depends on what they happen to encounter and who happens to share it. Communication gaps between care team members reflected this informal structure. Nurses and assistant nurses described learning about patients’ diagnoses by reading records or asking physicians rather than through formal handover. Psychosocial concerns were frequently delegated to social workers, who were described as responsible for emotional support and family communication, reflecting both role boundaries and language considerations, with Arabic-speaking social workers better positioned to communicate with patients and families.

Familiarity with person-centred care concepts was limited across all participant groups, with terminology frequently unfamiliar or conflated with patient-centred care. The analytical interest of the findings lies not only in what participants did not know but in the gap between formal vocabulary and actual practice. G2 illustrated this most fully. When asked about person-centred care, the initial response was: “I’m not familiar with that in practice, except maybe I’m doing it, but I don’t know that it is the terminology … No, actually nothing in my mind.” When pressed to describe their approach, the same physician offered: “I do it. I’m the best one to do this, but I don’t know the name … We are not disease concerned, we are whole person concerned, holistic application.” (G2) This trajectory from unfamiliarity with the concept to a confident claim of its practice captures a pattern evident across participants: the principles of person-centred care were present in how practitioners described their work, even where the formal framework was unknown. G4 similarly conflated person-centred with patient-centred care: “Patient-centred care? Person-centred care? Sorry, no … we are trying to do all our intervention and care just for what is good for the patient.” (G4) G1 acknowledged uncertainty about whether any formal policies governed such approaches: “I don’t know if there are any particular guidelines or any particular policies that are followed about it.” (G1)

Among nurses and assistant nurses, person-centred practice was evident in routine acts of personalisation that were described naturalistically rather than in any formal framework. AN2 described the rationale for offering choice: “For example, you will explain and he will tell no, I don’t want to go now, I want to sit for a while. Maybe he will sit five minutes then he will go, but at least he did what he want … Yeah, yeah, the choice is better.” (AN2) The same participant offered a broader orientation toward patient acceptance: “We should know that they are not changing their behaviour because they mean to do that, no, [it is] because they have a problem. We have to accept them as what they are doing, we have to be patient with them.” (AN2) These accounts suggest that dementia care training in this context need not introduce person-centred care as an entirely foreign concept; the intuitive practices already present in routine care provide a foundation on which structured training could build.

The workforce findings connect directly to the grey literature’s documentation of limited, unstandardised, and non-dementia-specific training across the sector. Where the grey literature identified the absence of standardisation across private training providers and the lack of cultural tailoring in available caregiver courses, the interviews reveal what that gap looks like from inside the public healthcare workforce: nursing staff who have received no dementia-specific preparation, physicians who deliver informal knowledge transfer during clinical rounds as a substitute for structured education, and a care team in which person-centred care principles are practised intuitively but without the formal frameworks that would make those practices consistent, transferable, and developable. The convergence of these findings across documentary and experiential sources strengthens the case for a structured, culturally informed dementia workforce training programme as a priority intervention.

### Day-to-day care practices and challenges

Physicians described a systematic approach to managing behavioural changes: first ruling out physical triggers such as infection, pain, or constipation; then attempting non-pharmacological strategies before considering medication. These non-pharmacological strategies included establishing sleep rhythms, stimulating patients during the day, managing environmental factors such as noise and lighting, and keeping patients “surrounded by familiar faces” (G4). The emphasis on identifying triggers before escalating to medication reflected a shared clinical orientation across physician participants, even where the formal frameworks underpinning such approaches were unfamiliar.

Nurses and assistant nurses described relying on teamwork to address challenges that exceeded their individual capacity. When their own interventions were insufficient, they called on social workers, physicians, or Arabic-speaking colleagues. Social workers were described as responsible for psychological support and family communication, with Arabic-speaking staff better positioned to address the emotional and relational dimensions of care. This division of labour was not simply a matter of professional role boundaries but a reflection of the absence of dementia-specific training that might otherwise equip nursing staff to address psychosocial needs directly. The structural consequence is a care model in which emotional and psychological support is consistently delegated rather than integrated into nursing practice, leaving a gap that social workers alone cannot reliably fill. On difficult days, the limitations of this arrangement were felt directly: as AN3 observed, “it happens sometimes, when you can’t speak with them and you cannot make them happy or cannot change the situation, like that, but I think this is normal.” (AN3) The normalisation of that difficulty, i.e., the sense that being unable to reach or comfort a patient is simply part of the work, reflects a workforce that has learned to absorb the consequences of structural gaps rather than expect them to be addressed.

The absence of community-based respite services was identified as a significant gap, with physicians describing family caregiving as unsustainable over the long term without structured relief. G1 articulated both the need and the cultural parameters within which any solution would need to operate: “I would suggest something like a respite care setup where at least the primary caregivers have some respite from the constant … this [type of] caregiving for a dementia patient is very exhausting. You’re looking at ten years, fifteen years, twenty years. Something that wears down the primary caregiver … A daycare centre kind of setup … those are the things I think will have good social impact.” (G1) G2 extended this argument by addressing the cultural resistance that dedicated dementia facilities face, and the reframing required to overcome it: “I would like people here to understand the importance of having, in the future, long-term institutions for dementia patients, for better quality of their life and safety … not to be considered like an elderly home, not to be considered like you’re throwing your loved one in a place. No, we can help them more … We need to change the image a little bit. Not everything outside the home is negative.” (G2) Together these accounts reveal a workforce that has identified both the need and the cultural pathway toward addressing it, in the absence of any policy framework to act on either.

Nurses and assistant nurses also described particular difficulty with patients in the early stages of dementia, whose needs were less predictable and whose communication was more variable. As RN1 observed, early-stage dementia is “difficult to manage” precisely because behavioural and emotional needs are less legible than in later stages when patients are bedridden and their care routines more established. Physical demands were also noted: patients who walked continuously, who forgot to swallow food, or who denied having been fed moments after eating. The emotional dimension of this work was acknowledged with characteristic restraint. AN2 described the relational effort of engaging with patients as a reciprocal source of meaning: “I feel happy, really and I think also they are feeling happy, because they are laughing some … they are happy.” (AN2) The same participant captured the motivation underlying that effort: “we are feeling that this is same as our family, just put yourself … I am putting myself in my home when I’m sitting my home and my kids all they are busy with the devices, and they are not talking with me I feel bad, for that one I am sitting here.” (AN2) These accounts suggest a workforce that sustains its practice through relational commitment in the absence of the formal training and structural support that would make that practice more effective and less personally costly.

The day-to-day care findings connect directly to the grey literature’s documentation of episodic and small-scale community initiatives and the absence of sustained respite or community-based support services. Where the grey literature identified the gap at the level of service provision, the interviews show its consequences for the people working within it: clinicians who have identified the need for respite infrastructure and articulated culturally appropriate models for it, nursing staff navigating psychosocial demands without the training to address them systematically, and a workforce sustaining care through personal commitment rather than institutional support. The convergence of these findings across both components of the environmental scan points to a care environment in which the informal sector involving families, domestic helpers, agency nurses, and committed but undertrained facility staff is bearing a burden that formal policy and service infrastructure have not yet been designed to address.

### Summary

Across the interviews, participants described a care environment characterised by high but undocumented prevalence, delayed diagnosis, reliance on antipsychotic medication, limited dementia-specific training, and family-centred care arrangements increasingly supplemented by private care agencies. Physicians emphasised systemic issues, including the absence of local data, the shortage of specialists, the lack of respite services, while nurses and assistant nurses focused on the practicalities of daily care and the challenges of responding to patients whose needs they could not always interpret or address. Person-centred care concepts were unfamiliar in formal terms, though small acts of personalisation were evident in routine care practice. Together, these accounts depict a workforce navigating dementia care with limited infrastructure, training, and support, while remaining committed to the wellbeing of patients and families within the constraints of the current system.

### Integration of findings

Several patterns recurred across the analyses of both grey literature sources and interviews with healthcare professionals: the absence of dementia-specific policy recognition, fragmented and uncoordinated services, the centrality of families in caregiving, reliance on private providers and domestic helpers, and gaps in workforce training. The consistency of these patterns across documented policies and lived professional experience strengthens confidence in the findings. As Table 3 illustrates, the two components of the environmental scan converge on several key areas, including the absence of dementia-specific policy, service fragmentation, family centrality, workforce training gaps, and the prevalence data deficit while each also makes distinctive contributions. The convergence of findings from independent documentary and experiential data sources strengthens confidence in the identified patterns and supports their use as a foundation for future policy and training development.

**Table 3:** Integrated summary of environmental scan findings across grey literature and interview components

Key Area	Grey Literature Findings	Interview Findings	Convergence
Policy recognition	Dementia absent from explicit policy; embedded within broader ageing and wellbeing agendas for older citizens	Not directly discussed, but systemic gaps attributed to absence of national planning	Converge: both sources document dementia’s invisibility in formal policy
Family as default caregiving unit	Policy documents and media position family as primary care unit; cultural and religious framing	Physicians describe high family involvement and evolving supervisory role over domestic helpers and agency nurses	Converge: both identify family centrality; interviews reveal delegation to domestic workers
Service fragmentation	Services exist across public hospitals, private clinics, and home-care agencies without coordination or documented pathways	Professionals describe navigating fragmented referral routes; families unsure which specialists to consult	Converge: both identify fragmentation; interviews reveal consequences for diagnosis and continuity
Private sector expansion	Growth of licensed home-care agencies and private training providers operating outside national integration	Physicians describe families contracting private agency nurses; quality and standards variable	Converge: both document private provision; interviews add lack of oversight
Access stratified by citizenship	Government entitlements specified for “Senior Emiratis”; expatriate access limited to employer-sponsored insurance or private provision	Not directly discussed by participants in these terms	Grey literature only: not raised by clinical participants
Workforce preparation	Training variable, unstandardised, and not dementia-specific; caregiver courses cover general older adult care	Nurses report no specialised dementia training; PCC terminology unfamiliar; learning occurs informally through observation	Converge: both identify training gaps; interviews reveal informal learning strategies
Local data and prevalence	Academic and media sources note absence of epidemiological data	Physicians estimate prevalence far exceeds official figures; attribute data gaps to absent research infrastructure	Converge: both identify data gaps; interviews add clinical estimates of underreporting
Diagnostic pathways and management	Not addressed in detail by grey literature sources	Late diagnosis common; reliance on antipsychotics (60–70%); absence of neuropsychological services in government facilities; language barriers manageable	Interview only: practice-level detail not captured in grey literature
Community and respite services	Awareness campaigns and academic initiatives documented as episodic and small-scale	Physicians identify absence of respite services as a significant gap contributing to family burnout and pharmacological management	Converge: both identify gaps in community support; interviews describe consequences

At the same time, each component made distinctive contributions. The grey literature captured the formal dementia care landscape: national policies that address ageing without naming dementia, service announcements that lack detail on continuity or care pathways, episodic NGO and academic initiatives, and the stratification of access by citizenship status. The interviews added experiential depth: late diagnosis resulting from delayed help-seeking and navigation challenges, antipsychotic use serving the dual purpose of managing patient symptoms and alleviating family burden, communication gaps within care teams, unfamiliarity with person-centred care terminology despite evidence of personalised practice, and the absence of community-based respite services.

Where the grey literature documented fragmentation, the interviews described its consequences. Where the grey literature noted family centrality as a policy framing, the interviews revealed how this plays out in practice - families supervising nurses from care agencies, cultural expectations shaping care arrangements, and the implications of long-term caregiving without respite. Together, these components provide a layered account that neither source could offer alone: a formal mapping of the landscape and an experiential account of navigating it. These findings establish a baseline understanding of the dementia care environment in the UAE and identify key issues that warrant closer consideration in the discussion that follows.

## Discussion

### Overview of key findings

This study set out to produce a comprehensive account of dementia care in the United Arab Emirates through an environmental scan combining analysis of UAE-focused grey literature with interviews with healthcare professionals directly engaged in dementia care delivery. The discussion that follows interprets the findings in relation to existing research, policy frameworks, and cultural considerations, with particular attention to how the two components inform and contextualise one another and what their convergence implies for service development, workforce training, and policy in the UAE.

The findings extend the peer-reviewed literature in two directions. By examining UAE-specific grey literature sources systematically, the study produces a ground-level documentary map of the care landscape, naming the specific facilities, policies, providers, and actors involved and establishing what each does and does not address. By documenting the perspectives of healthcare professionals navigating that landscape in daily practice, the study connects the structural features of the formal landscape to their consequences at the level of clinical decision-making, caregiving arrangements, and workforce preparation. The variability across the two components, with some patterns appearing strongly in practice but absent in formal documentation and others framed positively in policy discourse but contested in lived accounts, is itself analytically significant, underscoring how dementia care in the UAE is constructed differently across knowledge domains. The following discussion evaluates these patterns, explores their convergences and divergences, and considers their implications for policy, research, and practice.

### Grey literature findings in context

The grey literature findings position dementia as a relatively underdeveloped domain within the UAE’s otherwise advanced healthcare system [20, 94]. Despite policy frameworks that address older adult wellbeing, dignity, and inclusion, the consistent absence of dementia as a named priority suggests that the condition has not yet been recognised as requiring dedicated resources, planning, or service infrastructure [4, 20]. This gap between broad policy aspiration and condition-specific action is significant: it means that emerging services and initiatives operate without the coordinating framework that a national dementia strategy would provide.

The independent contribution of the grey literature analysis warrants explicit articulation. The peer-reviewed literature has established that dementia is absent from Gulf national policy frameworks, that services are fragmented, and that family caregiving is the normative model [4, 5, 12]. The grey literature analysis adds the specific documentary evidence underlying these observations: the identification of which policy instruments were examined and what each contains; a current mapping of thirteen private hospital and clinic sources and what they do and do not document about their provision; the specific figures on the scale of private home-care agency operation; the insurance exclusion finding that has not previously been documented in published research on this context; the inventory of community and NGO actors and their operating conditions; and the naming of the specific facilities whose entitlements are restricted to Emirati citizens. This level of specificity is unavailable in the peer-reviewed literature and cannot be derived from the interview data alone, and the finds from the grey literature analysis constitutes a baseline documentary record of the UAE dementia care landscape that provides the infrastructure for future monitoring, policy development, and research.

The documented growth of private provision, such as licensed home-care agencies and private hospitals offering dementia-related services independently of national planning reflects market responsiveness to unmet need but raises questions about standardisation, quality assurance, and equity of access. The stratification of services by citizenship status, with government entitlements specified for Emirati citizens and expatriates largely dependent on private provision, emerged as a significant structural feature of the care landscape.

### Interview findings in context

The interviews illustrated how the systemic limitations documented in the grey literature translate into daily care routines. The absence of dementia-specific training and local research capacity [4, 20] left professionals reliant on informal learning strategies and pharmacological management when non-pharmacological alternatives were both unknown and inaccessible. Cultural expectations of family-led care coexisted with growing reliance on domestic helpers and private home-care agencies, reflecting a care model in transition - one in which the cultural framing of family responsibility persists while the practical arrangements increasingly involve delegation and supervision [6]. Critically, the availability of domestic support structures, such as drivers, helpers, and contracted care workers may compensate for early functional decline, inadvertently masking the behavioural and cognitive changes that would otherwise prompt families to seek clinical evaluation.

The concepts of personhood and person-centred care were unfamiliar in formal terms, frequently conflated with patient-centred care [95, 96]. Although participants were unfamiliar with the formal terminology of person-centred care, the practices described by nurses and assistant nurses, such as offering choices, reading non-verbal cues, adjusting approaches based on patients’ moods and individual responses, and attending to personal preferences align with core elements of established PCC frameworks, including Kitwood’s [95] emphasis on recognising personhood and Brooker’s [96] VIPS model (Valuing people, Individualised care, Personal perspectives, Social environment). This suggests that dementia care training in this context could build on existing intuitive practices rather than introducing person-centred care as an entirely foreign concept.

The pattern of antipsychotic prescribing warrants particular attention. Participants described prescribing as shaped not only by clinical presentation but also by the need to alleviate family caregiver burden, indicating that treatment decisions were influenced by systemic pressures as much as by individual patient need [97, 98]. This prescribing pattern is particularly concerning given the well-documented adverse effects of antipsychotic use in dementia populations, including increased mortality, cerebrovascular adverse events, Parkinsonism, sedation, and accelerated cognitive decline [99, 100]. Regulatory agencies including the US FDA and the European Medicines Agency have issued explicit warnings against the routine use of antipsychotics in dementia [100]. The reliance on pharmacological management in this context reflects not individual clinical decisions but system-level deficits: the absence of dementia-specific training in non-pharmacological approaches, the lack of community respite services to alleviate family burden, and the limited availability of multidisciplinary behavioural management teams. This finding connects the workforce training deficit to prescribing practice: without access to non-pharmacological approaches or community respite services, pharmacological management becomes the default response to behavioural changes that the wider system is not equipped to address.

The analysis also revealed differences in emphasis across participants: physicians focused on systemic issues such as the absence of local data, the shortage of specialists, and the lack of respite services, while nurses and assistant nurses focused on the practicalities of daily care and the challenges of responding to patients whose needs they could not always interpret or address. Together, these perspectives depict a dementia care landscape shaped simultaneously by systemic constraints, professional competencies, ethical considerations, and pragmatic responses.

### Integration of findings

Across the two components, the same systemic barriers surfaced independently: the invisibility of dementia in national planning, fragmented services without clear care pathways, the centrality of family caregiving, and a workforce operating without dementia-specific preparation. At the same time, each component made distinctive contributions. The grey literature captured the formal architecture of policies and services, while the interviews revealed its consequences in practice - delayed diagnoses, families navigating opaque referral routes, and medication used to manage what systems cannot support. The contrast between how family caregiving is framed in policy and how it is experienced in practice is particularly significant. Policy documents and media coverage celebrate family duty as a cultural strength [41, 42], yet the interviews revealed the difficulty of sustaining this role without structural support. This disjuncture between idealised discourse and lived reality points to a vulnerability that current policy frameworks do not adequately address [5, 101]. The role of non-governmental actors appears inconsistent and often peripheral. Community awareness campaigns, university-led events, and private training initiatives were documented but remained episodic and disconnected from national planning. The interviews reinforced this gap, highlighting the absence of respite and community-based services. This unevenness underscores both the potential of non-governmental contributions and their limitations when left outside national coordination.

The findings also reveal glimpses of optimism: new memory clinics, awareness campaigns, and training initiatives suggest that momentum is building, albeit unevenly. The challenge, therefore, appears to lie less in the absence of activity and more in the absence of coherence, integration, and cultural tailoring. Taken together, these patterns depict dementia care in the UAE as a field that is at a transitional stage and no longer invisible, but not yet fully integrated into health and social care systems. That both documentary and experiential sources independently identified the same systemic barriers, including policy invisibility, fragmented services, limited training, and family burden lends additional credibility to these findings and suggests that they represent robust features of the care landscape rather than artefacts of any single data source.

### Policy and cultural dimensions

The extent to which dementia care in the UAE is shaped by the interaction of cultural norms and governance structures warrants specific consideration. While family-led care aligns with long-standing Gulf traditions of filial duty, the increasing strain on households as the ageing population grows raises questions about the sustainability of this model without formal support [5, 6]. The growing reliance on domestic helpers and private healthcare agencies introduces additional complexities, including variability in training, language and cultural barriers, and limited familiarity with dementia-specific needs [4].

At the policy level, the UAE reflects the broader Gulf model of citizen-centric welfare provision, where nationals are entitled to comprehensive, government-funded health and social services, while expatriates access care primarily through employer-sponsored health insurance [9, 10, 102]. From a Western welfare-state perspective, such arrangements may appear inequitable, but in the Gulf context, they reflect a governance model that prioritises citizens as the primary beneficiaries of state-funded welfare [103]. Within this model, expatriates rely on employment-based or private-sector provision, resulting in marked variation in service accessibility and continuity. Nevertheless, given that expatriates constitute approximately 88% of the UAE’s resident population [1, 2], this structural divergence raises fundamental questions about the sustainability and equity of dementia care provision. Unlike conditions that present acutely, dementia requires sustained engagement with diagnostic, therapeutic, and support services over years. Such services may not be adequately covered by employer-sponsored insurance or available through the private market. For the majority expatriate population, the absence of a pathway into publicly funded dementia care represents not merely a gap but a systemic exclusion from the care infrastructure. Addressing this will require policy responses that extend beyond the citizen-centric welfare model that has characterised Gulf health governance [103].

The absence of a national dementia strategy further compounds these challenges [4, 5]. Although policy aspirations for improved care of older citizens have been articulated in various government visions and health strategies [3], dementia-specific planning remains fragmented. This policy gap contributes to reliance on ad-hoc initiatives by individual emirates, private institutions, or NGOs, leading to uneven service availability [20, 104]. The absence of such a strategy places the UAE behind comparable high-income nations, including Australia, Canada, Japan, and several Western European countries, that have adopted dedicated national dementia plans aligned with the WHO Global Action Plan on Dementia [105, 106]. Developing an equivalent framework for the UAE would provide the coordinating infrastructure currently lacking across services, workforce training, and research. Together, these findings suggest that any meaningful expansion of dementia care in the UAE must navigate the intersection of cultural expectations, demographic pressures, and citizen-expatriate distinctions while advancing toward more integrated and dementia-specific policy frameworks.

### Contribution of the study

This study makes several contributions to the understanding of dementia care in the UAE. First, it represents the first comprehensive environmental scan of dementia care services in the UAE, integrating evidence from grey literature sources and perspectives with healthcare professionals. By examining findings from both documented policies and professional experiences, the study provides a more complete picture of the UAE dementia care landscape than either source could offer alone, capturing both formal structures and the realities of navigating them. Second, the study addresses a critical evidence gap. Research on dementia care in the Gulf remains fragmented and often limited to narrow foci such as caregiver burden, genetic factors, and pharmacological management, with little attention to systemic service delivery or national-level strategies [4]. By generating UAE-specific evidence from grey literature and experiential accounts from healthcare professionals, the present study consolidates baseline evidence that has not previously been assembled. Third, the study advances understanding of how cultural and policy contexts shape dementia care. It demonstrates that the governance model distinguishing citizens from expatriates has direct implications for access and entitlement, while cultural expectations of family care continue to structure day-to-day practices [6]. Bringing these dimensions together underscores the importance of designing dementia care strategies that are not only evidence-based but also culturally and contextually grounded [4]. Finally, by producing baseline data on the scope, distribution, and challenges of dementia care services in the UAE, this study offers a foundation for future policy development, workforce training initiatives, and the design of culturally appropriate dementia care services [20]. It provides both a descriptive map of the current landscape and a foundation for future action.

### Strengths and limitations

This study has several notable strengths. It is the first to undertake a comprehensive environmental scan of dementia care services in the UAE, combining systematic analysis of grey literature with interviews with healthcare professionals. The use of two complementary data sources allowed findings from policy documents, service descriptions, and practitioner perspectives to be examined together while preserving the distinctiveness of each. Methodological transparency was maintained through the use of structured extraction frameworks and detailed documentation of sources. Both components were analysed using a qualitative descriptive approach [33, 34], which prioritises comprehensive summary and low-inference interpretation. This approach is well-suited to applied health services research where the aim is to describe phenomena and identify patterns relevant to practice and policy [35]. Findings were generated inductively through iterative engagement with the data, and attention was paid to both consistencies and tensions across accounts. The convergence of key patterns across both components, including the absence of dementia-specific policy recognition, service fragmentation, family centrality, and workforce training gaps, strengthens confidence in the findings.

At the same time, certain limitations should be acknowledged. Grey literature searches are inherently incomplete and time-bound, and it is possible that some relevant local initiatives were not captured [22–24]. The sample for the interest-holder interviews was relatively small and focused on healthcare professionals in the public sector, which means that perspectives from private sector providers, family caregivers, people living with dementia, and policymakers were not directly represented. The study’s focus on the UAE limits the generalisability of findings to other countries, although the patterns identified are likely to resonate in contexts with similar demographic and governance structures, such as the other Gulf states.

A further consideration regarding the interview sample is that all participants had substantial professional experience, with a minimum of ten years of overall professional tenure. While this facilitated rich, experienced-practitioner accounts of systemic challenges and established care practices, it means that the perspectives of less experienced staff, who may carry different assumptions, be more recently trained, or be less embedded in existing care cultures, are not represented in this study. Future research should include practitioners at earlier career stages to provide a fuller account of the workforce landscape.

### Implications for practice, policy, and research

The findings of this study carry several implications for practice, policy, and future research. At the practice level, the limited dementia-specific training identified across both components underscores the need for structured dementia care education programmes [4]. Enhancing the competencies of healthcare professionals is essential to reduce reliance on pharmacological interventions and promote non-pharmacological, person-centred approaches to dementia care. Support for family caregivers also requires strengthening, whether through dementia care training, counselling, or respite services [6]. The growth of private home-care agencies as a pillar of dementia care delivery underscores the need for standardised accreditation and regulatory oversight of these services. Currently, private providers operate without formal integration into national care pathways, and the quality and scope of their dementia-specific provision remain undocumented.

At the policy level, the absence of a coordinated national dementia strategy in the UAE represents a critical gap [4]. While sector-specific initiatives and NGO activities demonstrate promising efforts, they remain fragmented and uneven in scope. Developing a comprehensive, integrated dementia strategy at the federal level would provide the structure needed to harmonise existing initiatives, align them with international standards, and ensure equitable access across the country. Such a strategy should also explicitly address the distinct needs of expatriate populations, who constitute the majority of the UAE’s residents but often fall outside citizen-centric entitlement frameworks [10]. The establishment of a national dementia registry would also provide a mechanism to address the prevalence data gap identified by participants and to enable systematic monitoring of diagnostic pathways, treatment patterns, and care quality over time. The Swedish Dementia Registry (SveDem), which tracks over 28,000 patients across specialist, primary care, and nursing home settings [107], offers a model that could be adapted to the UAE context.

At the research level, this study highlights the need for systematic investigation of dementia care services and practices in the region. Future research should move beyond descriptive mapping to develop and evaluate tailored dementia care interventions, assess the impact of culturally adapted training and awareness programmes, and explore innovative care models suited to the region’s contexts [4]. Longitudinal studies are needed to capture the evolving needs of persons living with dementia and their families, while cross-country comparisons within the Gulf region could help identify transferable practices and policy lessons.

### Conclusion

This study provides the first comprehensive environmental scan of dementia care in the United Arab Emirates, drawing together evidence from UAE-specific grey literature and the perspectives of healthcare professionals. The findings reveal a landscape that is simultaneously marked by strong health infrastructure and policy ambitions yet constrained by fragmented services, the absence of dementia-specific policy recognition, reliance on family and informal caregivers, and limited dementia-specific training and education. The structural distinction between citizens and expatriates further shapes access and provision, highlighting the need for coordinated, culturally informed approaches to dementia care. By consolidating diverse sources of evidence, this study establishes a baseline that can guide future research, policy development, and dementia care education, while also offering insights of relevance to other Gulf states facing similar demographic and cultural dynamics.

## Electronic supplementary material

Below is the link to the electronic supplementary material.

Supplementary File 1

Supplementary File 2

Supplementary File 3

## Data Availability

All data generated or analysed during this study are included in this published article and its supplementary information files.
