# Supplementary material for: Dementia care in the United Arab Emirates: an environmental scan of services, policy, workforce, and caregiving contexts
Source: BMC Health Serv Res. 2026 May 26;26:951. doi: 10.1186/s12913-026-14598-9 (PMC13356796; doi:10.1186/s12913-026-14598-9)
Supplement: Supplementary file 1 [file 12913_2026_14598_MOESM1_ESM.docx]

**Supplementary File 1: Grey Literature Search Strategy and Data Extraction**

# Grey Literature Search Strategy

A structured grey literature search was conducted between July and August 2025. Sources were identified through manual browsing of institutional websites, targeted Google searches, and forward/backward snowballing from included sources. The following table documents the source categories searched, specific entities and platforms targeted, search methods employed, search terms used, and the search period. Search terms were applied individually and in combination, adapted to the search functionality of each source (e.g., internal site search, Google site-restricted search, manual navigation of relevant sections).

| **Source Category** | **Sources Searched** | **Search Method** | **Search Terms** | **Search Period** |
| --- | --- | --- | --- | --- |
| Government portals and health authorities | UAE Government Portal (u.ae)  Ministry of Health and Prevention (mohap.gov.ae)  Dubai Health Authority (dha.gov.ae)  Department of Health Abu Dhabi (doh.gov.ae)  Emirates Health Services (ehs.gov.ae)  Sharjah Social Services Department (sssd.shj.ae)  Department of Community Development Abu Dhabi (pchr.gov.ae)  UAE Legislation Portal (uaelegislation.gov.ae)  Abu Dhabi Media Office (mediaoffice.abudhabi) | Internal site search and manual browsing of relevant sections (health services, policies, news, older citizens/seniors) | "dementia"; "Alzheimer"; "memory clinic"; "elderly care"; "senior citizens"; "older adults"; "ageing"; "cognitive impairment" | July–August 2025 |
| Public and private hospitals and clinics | Websites of hospitals and clinics identified through government health authority directories and Google searches | Manual browsing of service pages, neurology/psychiatry departments, and press releases | "dementia"; "Alzheimer"; "memory"; "neurology"; "geriatric"; "cognitive assessment" | July–August 2025 |
| Private home-based healthcare providers | Google search; Dubai Health Authority licensed providers list; health authority directories | Google search followed by manual review of provider websites and service descriptions | "dementia home care UAE"; "home nursing dementia Dubai"; "elderly home care Abu Dhabi"; "Alzheimer home care" | July–August 2025 |
| Non-governmental and community organisations | 4get-me-not Alzheimer's Organization; Alzheimer's Disease International member directory; Google search | Manual browsing of organisational websites, event pages, and campaign materials | "dementia awareness UAE"; "Alzheimer organisation UAE"; "dementia support group" | July–August 2025 |
| Academic and research institutions | University websites (e.g., Khalifa University, UAE University); Oxford Institute of Population Ageing; Salama bint Hamdan Al Nahyan Foundation | Manual browsing of research output pages, news, and reports | "dementia research UAE"; "ageing research Gulf" | July–August 2025 |
| News media and online publications | Google News; direct browsing of Khaleej Times, Gulf News, The National, Arabian Business, ARN News, Middle East Health, LiveHealthy.ae, UAE Stories | Google search and manual browsing of health/society sections | "dementia UAE"; "Alzheimer Dubai"; "elderly care UAE"; "memory clinic Dubai"; "dementia families UAE" | July–August 2025 |
| Caregiver training providers | Google search; referrals from other grey literature sources (snowballing) | Google search followed by manual review of training provider websites | "dementia training UAE"; "caregiver training Dubai"; "dementia care course" | July–August 2025 |
| Supplementary: Snowballing and cross-referencing | References and links identified within included sources | Forward and backward snowballing from included documents | N/A - identified through cross-referencing within included sources | Ongoing throughout search period |

1. **Summary of Grey Literature Sources by Category**

A total of 61 grey literature sources were included. Table S2 summarises the number of sources identified within each category. Sources were classified during data extraction based on the type of entity or publication from which they originated.

| **Source Category** | **Number of Sources** | **Typical Source Types** |
| --- | --- | --- |
| National healthcare policies, strategies, and plans | 9 | Government portals, legislation portals, media offices |
| Public healthcare services and initiatives | 12 | Health authority websites, government hospital pages, media releases |
| Private hospitals and clinics | 13 | Hospital and clinic websites |
| Private home-based healthcare providers | 6 | Provider websites, health authority directories |
| Non-governmental and community organisations | 5 | Organisational websites, ADI member directory |
| Academic and research contributions | 4 | University websites, research institute reports |
| Caregiver training providers | 3 | Training provider websites |
| News media and online publications | 9 | Online newspaper articles, health magazines |
| **Total** | **61** |  |

1. **Data Extraction Fields**

Information from each included grey literature source was extracted into a structured spreadsheet using the fields described below. The extraction process captured both descriptive details (e.g., type of service, target population) and interpretive elements (e.g., how dementia is framed in policy discourse, identified gaps in care provision). The complete extraction table with data for all 61 sources is available as a separate supplementary file.

| **Extraction Field** | **Description** |
| --- | --- |
| **Source category** | Classification of source type (e.g., government portal, private hospital, news media) |
| **Country** | Country of origin (UAE for all included sources) |
| **Focus area** | Primary topic addressed (e.g., policy, services, training, awareness) |
| **Source/Organization Name** | Name of the organisation, entity, or publication |
| **Publication date** | Date of publication or date of last access for undated web content |
| **Type of service/initiative** | Nature of the service, programme, or initiative described |
| **Specific dementia services** | Whether dementia-specific services were explicitly described and/or are available/applicable |
| **Target population** | Population served or addressed (e.g., Senior Emiratis, expatriates, general public) |
| **Key features/description** | Summary of main content, claims, or services described |
| **Cultural/religious/family considerations** | References to cultural values, family roles, religious practices, or gendered norms |
| **Dementia care service gaps/challenges noted** | Gaps, limitations, or unmet needs identified by or inferred from the source |
| **Links to source** | Web address of the source |

**Notes on Search Approach and Limitations**

The grey literature search was designed to be comprehensive within the constraints inherent to non-indexed sources. Unlike database searches for peer-reviewed literature, grey literature searching does not follow a standardised protocol and cannot be replicated with identical precision (Adams et al., 2017; Paez, 2017; Pedersen et al., 2023). The following points are noted for transparency:

**Search scope:** The search was restricted to UAE-specific sources in English. Sources were identified through targeted browsing of institutional websites, Google searches using the terms documented in Table S1, and snowballing from references and links within included sources. The search targeted entities known to be involved in health policy, service delivery, or public discourse on ageing and dementia in the UAE.

**Inclusion decisions:** Sources were included if they contained substantive information on dementia care services, policies, training, or related topics in the UAE. Sources that mentioned dementia only peripherally, duplicated content from already-included sources, or were no longer accessible at the time of searching were excluded. Inclusion decisions were made by the first author and reviewed with the supervisory team.

**Temporal coverage:** Included sources span publication or access dates from 2014 to 2025. Many web-based sources (e.g., hospital service pages, organisational profiles) were undated; for these, the access date was recorded.

**Reproducibility:** Given the dynamic nature of web-based content, some sources may have been updated, relocated, or removed since the search was conducted. URLs were verified at the time of final manuscript preparation. The inherent limitations of grey literature reproducibility are acknowledged in the manuscript (see Strengths and Limitations).
