# Supplementary material for: Dementia care in the United Arab Emirates: an environmental scan of services, policy, workforce, and caregiving contexts
Source: BMC Health Serv Res. 2026 May 26;26:951. doi: 10.1186/s12913-026-14598-9 (PMC13356796; doi:10.1186/s12913-026-14598-9)
Supplement: Supplementary file 2 [file 12913_2026_14598_MOESM2_ESM.docx]

Supplementary File 2: Grey literature sources included in the environmental scan, organised by source type

| **No.** | **Source Category** | **Country** | **Focus Area** | **Source/Organization Name** | **Publication Date** | **Type of Service/**  **Initiative** | **Specific Dementia Care Services Available/**  **Applicable** | **Target Population** | **Key Features or Description** | **Cultural/Religious/Family Considerations** | **Dementia Care Service Gaps/Challenges Noted** | **Link to Source** |
| --- | --- | --- | --- | --- | --- | --- | --- | --- | --- | --- | --- | --- |
| **1** | **National Healthcare Policies, Strategies, & Planning** | UAE | Government support & policy | UAE Government Portal (u.ae) | Updated 2025 | Social services and benefits for senior Emiratis | Not Applicable | Senior Emiratis (age 60+) | Various benefits including health insurance, home care services, priority in government service queues, and family support services; Abu Dhabi and Dubai offer slightly different packages | Emphasis on family cohesion and caregiving within the family unit; "home care" framed in the context of cultural respect for elders | No specific mention of dementia or specialized dementia care; details are general and nonspecific | [Link](https://u.ae/en/information-and-services/social-affairs/senior-emiratis) |
| **2** | **National Healthcare Policies, Strategies, & Planning** | UAE | Policy and planning | UAE Legislation Portal – Federal Government | Updated 2025 | National Policy for Senior Emiratis | Not Applicable | Senior Emiratis | Focus on improving physical, psychological, and mental health of older adults, including mental health and neurodegenerative conditions; commitment to expanding access to specialized services | Emphasis on values of dignity, protection, inclusion, and family support | Policy remains high-level; no specific mention of dementia or implementation mechanisms; unclear what services currently exist | [Link](https://www.uaelegislation.gov.ae/en/policy/details/lsy-s-lotny-lkb-r-lmo-tnyn) |
| **3** | **National Healthcare Policies, Strategies, & Planning** | UAE | Policy and wellbeing | UAE Government Portal – Federal Strategy | Updated 2025 | National Strategy for Wellbeing 2031 | Not Applicable | All UAE residents, including older adults | Aims to improve quality of life through 14 components and 9 strategic objectives, including good mental health, active lifestyle, and cohesive society. Highlights the role of healthcare, environment, and work-life balance. | Encourages positive thinking, community engagement, and supportive social values | No specific mention of dementia or older adults; broad strategy without dementia-specific indicators or measures | [Link](https://u.ae/en/about-the-uae/strategies-initiatives-and-awards/strategies-plans-and-visions/social-affairs/national-strategy-for-wellbeing-2031) |
| **4** | **National Healthcare Policies, Strategies, & Planning** | UAE | Healthcare system overview | UAE Ministry of Foreign Affairs | Updated 2025 | Overview of the UAE healthcare system | Not Applicable | General population, includes older adults | Describes the UAE’s comprehensive, government-funded and private-sector healthcare system. Notes dual-level governance (federal and emirate). Includes preventive, curative, and rehabilitative services. | Not directly discussed | No specific mention of dementia or elder care services | [Link](https://www.mofa.gov.ae/en/Missions/Paris/The-UAE/Health-Care) |
| **5** | **National Healthcare Policies, Strategies, & Planning** | UAE | Healthcare strategy | U.AE (Federal Government Portal) | Updated 2025 | National Healthcare Strategies and Policies | Not Applicable | General population, includes older adults | Summarizes the UAE’s strategic health plans: UAE Vision 2021, National Agenda, National Health Strategy 2014-2021, and updated strategy 2023-2026. Focus on quality, accessibility, mental health, healthy aging, and staff capacity development. | Not explicitly discussed | Dementia care not mentioned; geriatric focus is indirect | [Link](https://u.ae/en/information-and-services/health-and-fitness/healthy-policy-and-laws) |
| **6** | **National Healthcare Policies, Strategies, & Planning** | UAE | Government strategy and policy | Department of Community Development – Abu Dhabi (PCHR) | Updated 2025 | National Policy for Senior Citizens | Not Applicable | Emirati senior citizens | The National Policy promotes rights to medical care, social participation, active aging, safety, and dignity; aims to integrate seniors in development across sectors; upholds intergenerational solidarity | Emphasis on family cohesion and preserving dignity of seniors; values align with Islamic and cultural norms | No specific mention of dementia or dementia-specific services or training; policy remains broad | [Link](https://www.pchr.gov.ae/en/priority-details/senior-citizens) |
| **7** | **Campaigns & Service Evaluations - Government Foundations** | UAE | Public awareness | Salama bint Hamdan al Nahyan Foundation | March 2024 (inferred from newsletter URL) | Awareness campaigns, healthcare staff training, and service enhancement through research | Accessibility not specified but efforts are UAE-based and collaborative with existing providers | Dementia patients and their families in the UAE | - Public awareness campaigns - Professional training for healthcare workers - Home visits - Research support - Coordination with government and private hospitals to enhance services | Implicit in the focus on home visits and collaboration with families; emphasis on support tailored to patient needs | One of the few initiatives integrating awareness, research, clinical training, and home-based care; could serve as a model for multi-level dementia interventions | [Link](https://www.shf.ae/en/what-we-do/health/#:~:text=Memory%20Program%3A%20A%20multifaceted%20effort%20to%20increase,services%20for%20dementia%20patients%20and%20their%20families.) |
| **8** | **Campaigns & Service Evaluations - Government Foundations** | UAE | Policy/service evaluation | Salama bint Hamdan Al Nahyan Foundation | Not Stated (Review period mentioned as December 2014) | Dementia Care System Review | Not directly available – focused on system review | Policymakers, healthcare planners, health professionals | A high-level review calling for an integrated dementia care system in the UAE. Emphasizes the need for early diagnosis, coordinated services, and trained staff, including the use of global best practices. | Culturally sensitive care mentioned as an emerging need, but not elaborated | Highlights fragmented care, lack of coordination across services, limited public awareness, and workforce gaps | [Link](https://shf.ae/newsletter/health/dementia-care-system-review.html) |
| **9** | **News Releases - Government Health Authorities** | UAE | Policy announcement via news media | Ministry of Health and Prevention (MOHAP) in coordination with national health and social entities | July 15 2024 | National framework for healthy ageing in the UAE | Available (general services for older adults; no explicit mention of dementia-specific services | Older adults in the UAE | Aligns with WHO’s healthy ageing framework; encourages early detection, health promotion, lifelong care, and intersectoral coordination | Indirectly (emphasizes holistic care, wellbeing, and quality of life); no explicit mention of cultural, religious, or family integration | Not mentioned | [Link](https://www.wam.ae/en/article/b46a5dp-mohap-highlights-national-framework-for-healthy) |
| **10** | **News Releases - Government Health Authorities** | UAE | Institutional service announcement | Sheikh Shakhbout Medical City (SSMC) in collaboration with Sakina, involving neurologists, psychiatrists, psychologists, and geriatricians - Memory Clinic at SSMC, Abu Dhabi | March 19 2025 | Memory assessment, early diagnosis, interdisciplinary evaluation, patient and caregiver education, individualized care plans | Available: memory assessment, early diagnosis, interdisciplinary evaluation, patient and caregiver education, individualized care plans | Older adults in the UAE | Launch of a multidisciplinary memory clinic to enhance early dementia detection and intervention | Implied through inclusion of caregiver education and person-centered care model; no explicit mention of culture or religion | Responds to gap in early detection and interdisciplinary dementia care in the region | [Link](https://www.mediaoffice.abudhabi/en/health/sheikh-shakhbout-medical-city-partners-with-sakina-to-launch-multidisciplinary-memory-clinic-to-enhance-early-detection-for-dementia/) |
| **11** | **News Releases - Government Health Authorities** | Gulf Cooperation Council (focus on UAE) | Projected dementia rise and system readiness | LiveHealthy.ae (Health journalism platform) | May 7 2023 | Awareness-raising article based on research findings | Not Available (focuses on projections, not services) | General population and healthcare policymakers | Article highlights a study predicting a 1000% increase in dementia cases in the GCC by 2050. Discusses healthcare system unpreparedness, lack of dementia plans, and call for strategic planning. | Brief mention of family responsibility but no cultural/religious adaptation of care is discussed | Lack of national dementia strategies in GCC countries; lack of trained professionals; absence of care pathways | [Link](https://livehealthymag.com/study-gcc-dementia-cases-to-jump-1000-percent-by-2050/) |
| **12** | **News Releases - Government Health Authorities** | UAE/MENA Region | Public health burden, need for preparedness | Middle East Health | March 1 2024 | Editorial/feature article (informative) | Not Available (mostly commentary on future needs) | General population and healthcare systems | Highlights regional unpreparedness for projected surge in dementia cases; calls for urgent investment in care infrastructure, research, and awareness campaigns. Mentions WHO collaboration and global dementia strategy. | Acknowledges cultural barriers to early diagnosis and stigma surrounding dementia in Arab societies | Major gaps in early detection, care infrastructure, policy frameworks, and workforce preparedness | [Link](https://middleeasthealth.com/medical-specialty-features/dementia/middle-east-faces-tidal-wave-of-dementia-cases/) |
| **13** | **News Releases - Government Health Authorities** | UAE | Healthcare service provision for older adults | Emirates Health Services (EHS) | October 1 2024 | Streamlined service model for senior citizens | Available | Senior citizens in UAE | EHS launched a pioneering model of care for older adults, with 14 integrated initiatives including proactive screening, chronic disease management, geriatric clinics, fall prevention, and palliative care. Emphasis on preventive services and enhancing autonomy of older adults. | Not explicitly discussed | No specific mention of dementia services or workforce training | [Link](https://www.ehs.gov.ae/en/media-center/news/emirates-health-services-streamlines-services-and-delivers-pioneering-care-for-senior-citizens) |
| **14** | **News Releases - Government Health Authorities** | UAE | Elderly healthcare service coverage | Emirates Health Services (EHS) | October 25 2023 | Comprehensive healthcare services for older adults | Available | Senior citizens and residents in the UAE | EHS highlights efforts to expand access to high-quality health services for older adults, including specialized clinics, preventive screenings, chronic disease management, home care, and telemedicine. Aims to promote well-being and ensure health equity among elderly citizens and residents. | Not explicitly mentioned | No mention of dementia-specific services or cultural tailoring | [Link](https://www.ehs.gov.ae/en/media-center/news/ehs-spotlights-elderly-care-for-citizens-and-residents) |
| **15** | **News Releases - Government Health Authorities** | UAE | A memorandum of understanding to promote integrated elderly care in Dubai | Dubai Health Authority (DHA) | March 26 2016 | Plans to enhance current care services | Not explicitly stated | Elderly population in Dubai | Announcement of a move towards promoting an integrated elderly care in Dubai. Plans for a series of workshops and conferences, as well as training for healhcare staff to improve the quality of care. | Mentions culturally sensitive care tailored to Emirati elders and community norms | No detailed mention of long-term follow-up, integration with community dementia care | [Link](https://www.dha.gov.ae/en/media/news/88) |
| **16** | **Care Services - Government Healthcare Service Providers** | UAE | Primary care and integrated health services | Department of Health – Abu Dhabi (DOH) | Not stated (active webpage) | Comprehensive, patient-centered primary care model | Not explicitly stated (likely available through integrated care) | All residents, including elderly and those with chronic conditions | Focuses on prevention, early detection, management of chronic diseases, and mental health. Promotes continuity of care through integrated services in primary care settings. | Emphasis on patient-centeredness may reflect cultural sensitivity, but no specific mention of dementia, family roles, or religious tailoring | No specific dementia care pathways or mention of geriatric or neurodegenerative specializations | [Link](https://www.doh.gov.ae/en/programs-initiatives/Primary-Care) |
| **17** | **Care Services - Government Healthcare Service Providers** | UAE | Elderly care services | Sheikh Shakhbout Medical City (SSMC) | Not stated (active webpage) | Hospital-based clinical service | Available | Patients with dementia and other cognitive conditions | Provides diagnostic and treatment services for various cognitive disorders, including Alzheimer’s. Multidisciplinary team includes neurologists and neuropsychologists. | No specific mention of cultural or family integration | Limited public information on care pathways, caregiver education, or dementia-specific support programs outside diagnostics. | [Link](https://ssmc.ae/doctors-specialities/dementias-other-cognitive-conditions/) |
| **18** | **Care Services - Government Healthcare Service Providers** | UAE | Health facility – Elderly care | Rashid Hospital Dubai | Not stated | Outpatient Geriatric Services | Available | Older adults, possibly including those with dementia | Offers general family medicine and geriatric care. While dementia is not explicitly stated, geriatric services may include cognitive screening or referral pathways. | Family-oriented name and scope suggest alignment with local familial caregiving structures. | Lack of specific mention of dementia-focused care/services | [Link](https://services.dha.gov.ae/sheryan/wps/portal/home/medical-directory/facility-details?facilityId=0047970) |
| **19** | **Care Services - Government Healthcare Service Providers** | UAE | Elderly care services | Al Mamzar Community Centre for Elderly | Not stated | A dedicated elderly care center | Available | Senior Emiratis | A center specialized in elderly care, offering geriatric, dementia, and memory care services. The facility includes comprehensive services such as screening, diagnostics, rehabilitation, psychiatric support, home care integration, and social support. It is designed to deliver multidisciplinary, preventive, and person-centered care. | Emphasis on home visits, family involvement, and social support reflecting local caregiving norms | Limited to Emiratis; unclear access for expats and wider dementia demographics | [Link](https://dubaihealth.ae/l/200344) |
| **20** | **Care Services - Government Healthcare Service Providers** | UAE | Elderly care services | Dubai Health - Seniors' Happiness Centre | June 3 2020 | Elderly care services at Al Safa Health Center (Specialized Geriatric Centre) | Available | Senior Emiratis | The center offers comprehensive geriatric seervices on inpatient as well as outpatient basis, including comprehensive geriatric care, early detection of cognitive decline, fall risk assessment, medication review, chronic disease management. The approach is multidisciplinary, covering mental health and physical wellbeing as well as rehabilitation and recreational services. | Family involvement is encouraged; services customized for senior Emiratis and home-based follow-up is provided | Mentions the increasing number of citizens above the age of 60%, highlighting the need for such comprehensive geriatric services. | [Link](https://mediaoffice.ae/en/news/2020/June/03-06/DHA-elderly-care) |
| **21** | **Care Services - Government Healthcare Service Providers** | UAE | Elderly cares/Social welfare | Sharjah Social Services Department (SSSD) | Not stated (active webpage) | Home care, day care, social inclusion programs for elderly | Not explicitly stated (likely available under elder care umbrella) | Senior Emiratis and older residents | Provides a range of elder care services: home visits, elderly day centers, mobile units, and family support programs. Emphasis on dignity, inclusion, and care in home/community settings. | Services emphasize family engagement and intergenerational support, consistent with local cultural norms; strong religious and social responsibility framing | No explicit mention of dementia-focused services, care pathways, or specialized training | [Link](https://sssd.shj.ae/) |
| **22** | **Care Services - Government Healthcare Service Providers** | UAE | Geriatric and elderly care | Obaidalla Geriatric Hospital | Not stated (active webpage) | Specialized geriatric and rehabilitation hospital | Available (geriatric and rehabilitation services suggest dementia-related care is offered) | Senior citizens (elderly population) | Provides inpatient and outpatient services for geriatrics, with focus on rehabilitation and long-term care. Services include physical therapy, chronic disease management, and elder-specific care. | No direct mention of religious or cultural considerations, but the facility likely aligns with national elderly policies that emphasize dignity and family involvement | No detailed information on dementia-specific protocols or integration with community/home care | [Link](https://www.ehs.gov.ae/en/services/health-care-facilities/obaidalla-geriatric-hospital) |
| **23** | **Care Services - Government Healthcare Service Providers** | UAE | General healthcare and geriatric services | Al Fujairah Hospital | Not stated (active webpage) | Government hospital providing comprehensive medical care | Not Specified (general hospital with potential for related services) | General population including elderly | Offers a wide range of specialties including internal medicine, neurology, psychiatry, rehabilitation, and geriatrics. Serves as a referral hospital in the region. Dementia not explicitly mentioned. | No direct mention; cultural and family considerations are likely embedded in general EHS service delivery models | No specific mention of dementia-focused programs or memory clinics | [Link](https://www.ehs.gov.ae/en/services/health-care-facilities/al-fujairah-hospital) |
| **24** | **Engagement - Academic Institutions/**  **Sources** | UAE | Book chapter title: Dementia care practices in the United Arab Emirates | Oxford University Press (from *Dementia Care: International Perspectives*) | 2019 | Dementia care practices | Limited public sector services; private care is more common, but expensive | Persons with dementia and caregivers | - Describes landscape of dementia care in the UAE - Highlights fragmentation of services - Notes the dominance of private providers - Discusses family caregiving and lack of professional support | Strong emphasis on family’s central caregiving role and cultural norms of filial duty | Calls for national dementia strategy, investment in workforce training, culturally appropriate services, and improved coordination of care | [Link](https://academic.oup.com/book/25044/chapter-abstract/189131943?redirectedFrom=fulltext) |
| **25** | **Engagement - Academic Institutions/**  **Sources** | UAE | Positive ageing and public policy | Oxford Institute of Population Ageing (University of Oxford) | June 20 2023 | Policy-level initiatives for ageing populations | Not Available (focuses on ageing more broadly) | Older Emiratis | The blog discusses the UAE’s public policy initiatives to support positive ageing, including the National Policy for Senior Emiratis. It highlights government efforts to support active ageing, inclusion, and wellbeing. | Recognizes strong family structures and religious values that support care of older adults; policies aim to work in tandem with these traditions | Lack of integration between private and public sectors; need for more data to inform policy; fragmented service landscape for elderly care | [Link](https://www.ageing.ox.ac.uk/blog/Positive-Ageing-and-the-Role-of-Public-Policy-in-the-United-Arab-Emirates) |
| **26** | **Engagement - Academic Institutions/**  **Sources** | UAE | Awareness and community engagement | United Arab Emirates University (UAEU) | May 1 2017 | University-led public awareness campaign | Not Available (focus on awareness, not services) | General public, students, healthcare professionals | Event organized by UAEU's College of Medicine with Alzheimer's Disease International and Reaya Home Healthcare Center to promote dementia awareness and early intervention | The event was held during the Holy Month of Ramadan, suggesting consideration for cultural and religious context | Limited focus on sustained services or structured training; awareness events are episodic | [Link](https://www.uaeu.ac.ae/en/news/2017/may/dementia.shtml) |
| **27** | **Engagement - Academic Institutions/**  **Sources** | UAE | Diagnosis, language barriers, cultural adaptation | Khalifa University | January 16, 2024 | Research article highlighting linguistic and cultural gaps in dementia diagnosis | Not available (focuses on diagnosis, not services) | Arabic-speaking older adults in the MENA region | Discusses challenges in cognitive assessment tools not being adapted linguistically/culturally; collaboration between KU and NYU Abu Dhabi to develop more inclusive tools | Strong emphasis on linguistic and cultural adaptation; notes lack of culturally grounded diagnostic frameworks | Inadequate culturally and linguistically appropriate diagnostic tools; potential misdiagnosis or underdiagnosis | [Link](https://www.ku.ac.ae/bridging-the-language-and-culture-divide-in-dementia-diagnosis-in-the-mena-region) |
| **28** | **News Releases - Local Media Sources/**  **Newspapers** | UAE | Specialist memory care | UAE Stories / Unspecified private provider | June 10 2024 | Alzheimer’s & Memory Care Center | Available | Older adults with Alzheimer’s and dementia | Describes a state-of-the-art facility providing early diagnosis, therapy, care coordination, and family support; includes brain health programs | Family support is mentioned as an integral part of care; attention to the region's growing need for specialized services | Lack of previous dedicated Alzheimer’s centers; increasing cases highlight the need for specialized infrastructure | [Link](https://uaestories.com/cutting-edge-centre-for-alzheimers/) |
| **29** | **News Releases - Local Media Sources/**  **Newspapers** | UAE | Opinion piece/not a study | Arabian Business contributor (name not specified) | February 8 2022 | Role of families, social inclusion, and community in supporting older adults; development of the longevity sector in the UAE | Not available: General reference to elder care, intergenerational support, and long-term care; not specific to dementia but relevant to contextual framing | Primarily families; emerging mention of state and private sector collaboration | General reference to elder care, intergenerational support, and long-term care; not specific to dementia but relevant to contextual framing | Yes — strongly emphasizes the traditional family responsibility for elderly care in Emirati society | Highlights the tension between growing elderly population and evolving care needs; notes that family-based systems may not remain sustainable alone | [Link](https://www.arabianbusiness.com/opinion/social-inclusion-for-old-age-people-in-the-uae-at-the-fore-in-emerging-longevity-sector) |
| **30** | **News Releases - Local Media Sources/**  **Newspapers** | UAE | Alzheimer's prevalence and public health awareness | Zawya (interview with Aster DM Healthcare experts) | September 11 2023 | Awareness and early detection efforts (mentioned generally) | Not clearly specified | General UAE population, with a focus on aging adults | Highlights rising rates of Alzheimer’s disease in the UAE due to increasing life expectancy. Experts call for more awareness, early screening, and public health strategies to address the issue. | Family burden and social taboos around dementia are briefly mentioned; culturally informed caregiving not emphasized | Lack of national-level screening programs; stigma and low awareness; minimal institutional support for long-term care | [Link](https://www.zawya.com/en/business/alzheimers-disease-could-become-far-too-common-in-uae-omatemjb) |
| **31** | **News Releases - Local Media Sources/**  **Newspapers** | UAE/MENA Region | Regional prevalence and public health response | Zawya (in collaboration with Alzheimer’s Disease International report) | September 21 2023 | Global report findings; public awareness campaign; policy call to action | Not available (service availability not discussed) | MENA region population, especially older adults | Cites new ADI report predicting 14 million people with dementia in MENA by 2050. Calls for increased awareness, infrastructure, and regional dementia plans. Highlights the fast-growing rates in countries like UAE, Egypt, and Saudi Arabia. | Cultural silence and stigma mentioned as barriers to early diagnosis and support | Lack of structured national dementia strategies in most MENA countries; shortage of trained healthcare workers | [Link](https://www.zawya.com/en/life/health/dementia-rising-rapidly-in-mena-region-almost-3mln-people-live-with-the-condition-gr215af3) |
| **32** | **News Releases - Local Media Sources/**  **Newspapers** | UAE | Innovation in early dementia detection | Health Tech World | March 6 2024 | Partnership between Cognetivity Neurosciences and Emirates Health Services | Available | General population, with focus on early detection | The deal will deploy AI-powered cognitive testing tools to help with early diagnosis of dementia. Cognetivity’s platform uses an iPad-based test that evaluates information processing without language bias, and has been validated in clinical trials. | No direct mention, but the tech’s ability to remove language/cultural bias is implied as beneficial in diverse populations | Implementation and integration challenges in healthcare systems not discussed in depth | [Link](https://www.htworld.co.uk/news/uae-steps-up-dementia-fight-with-cognetivity-deal/) |
| **33** | **News Releases - Local Media Sources/**  **Newspapers** | UAE | Dementia service provision | PureHealth / SceneNow | March 2024 | Opening of a memory clinic | Available | Individuals with memory impairments, elderly population | A state-of-the-art memory clinic has been launched in Abu Dhabi to improve dementia diagnosis and care, emphasizing early intervention, cognitive testing, and personalized care. | Not explicitly discussed | General reference to regional need for early diagnosis and access to quality care | [Link](https://scenenow.com/Buzz/PureHealth-Opens-Memory-Clinic-to-Advance-Dementia-Care-in-Abu-Dhabi) |
| **34** | **News Releases - Local Media Sources/**  **Newspapers** | UAE/MENA region | Dementia prevalence and awareness | Al Arabiya News / WHO / Alzheimer’s Disease International | September 21 2022 | Awareness and public education efforts | Not directly available (focus on prevalence) | General public and healthcare systems in MENA | Article highlights alarming rise in dementia cases across the MENA region; calls for urgent action and investment in healthcare infrastructure | Notes that stigma and low public awareness impede early diagnosis and care-seeking in some Arab cultures | Lack of public awareness, limited early detection services, insufficient care frameworks | [Link](https://english.alarabiya.net/News/gulf/2022/09/21/World-Alzheimer-s-Day-Dementia-growing-at-a-shocking-rate-in-MENA) |
| **35** | **News Releases - Local Media Sources/**  **Newspapers** | UAE | Public health framing of dementia | The National News / WHO / UAE health officials | March 30 2024 | Strategic recognition and framing of dementia as a mental health condition | Not directly available (policy framing focus) | National policymakers, healthcare systems | Dementia is officially being recognized and addressed as a mental health condition by UAE officials in collaboration with WHO; intended to improve care frameworks and resource allocation | Recognizes community awareness and family expectations as factors to consider in developing dementia services | Lack of mental health integration in dementia care previously; the shift requires system-wide coordination and education | [Link](https://www.thenationalnews.com/health/2024/05/30/who-states-dementia-mental-health/) |
| **36** | **News Releases - Local Media Sources/**  **Newspapers** | UAE | Dementia prevention and aging population | The National | October 1 2017 | Public awareness and risk reduction | Not available (focus on prevention and awareness) | General public, younger adults, aging population | Highlights the importance of lifestyle choices—such as exercise, diet, mental stimulation, and blood pressure control—in midlife to prevent dementia later | Cultural reference to UAE’s increasing life expectancy and changes in traditional lifestyle | Lack of national awareness campaigns; limited focus on dementia prevention in younger adults | [Link](https://www.thenationalnews.com/uae/lifestyle-changes-early-in-life-may-prevent-dementia-as-the-uae-s-population-ages-1.612931) |
| **37** | **News Releases - Local Media Sources/**  **Newspapers** | UAE | Early diagnosis and neuroscience-based innovation | ARN News / Dubai Health Authority | September 1 2023 | Launch of a diagnostic research center | Available (diagnostics) | Patients with neurological disorders including dementia | The center will advance early detection and diagnostics for neurological diseases, including dementia, using neuro-biochemical markers | Not mentioned explicitly | The need for early diagnostics signals prior limitations in detection capabilities | [Link](https://www.arnnewscentre.ae/news/lifestyle/menas-first-centre-for-advanced-neuro-biochemical-diagnostics-launched/) |
| **38** | **News Releases - Local Media Sources/**  **Newspapers** | UAE (Global context, with local coverage) | Prevention and risk reduction | Khaleej Times (reporting on a global study published in *The Lancet*) | September 21, 2023 | Not Available (global study discussed, no UAE-specific services mentioned) | Public dissemination of research findings | General public and policymakers | The article summarizes a *Lancet* study identifying 12 modifiable risk factors for dementia, and notes that many cases could be delayed or prevented through public health strategies | Not discussed | Lack of UAE-specific data or policy response; article does not connect global findings to local context | [Link](https://www.khaleejtimes.com/world/many-dementia-cases-could-be-prevented-but-far-from-all-study?_refresh=true) |
| **39** | **News Releases - Local Media Sources/**  **Newspapers** | UAE | Epidemiological trends and healthcare planning | Khaleej Times | September 24 2023 | Not Available | Report on projected rise in dementia cases | UAE population (general, future projections) | UAE projected to see a 1,795% increase in dementia cases by 2050. Article emphasizes urgency for planning, awareness, and investment in dementia care infrastructure | Brief reference to population aging and family support norms | Lack of current national dementia strategy, insufficient services to meet projected rise | [Link](https://www.khaleejtimes.com/lifestyle/health/uae-to-see-second-highest-percentage-increase-in-dementia-cases-globally?_refresh=true) |
| **40** | **News Releases - Local Media Sources/**  **Newspapers** | UAE | Coping strategies, caregiving experience, dementia awareness | UAE Times | October 1 2023 | Feature article on lived experiences and support systems | Available | People living with Alzheimer’s and their families | Highlights personal stories of families managing Alzheimer’s, including emotional toll, care responsibilities, and use of available services like memory clinics and support from organizations such as the Alzheimer’s Association | Mentions the importance of family involvement in caregiving and the impact on family dynamics; reflects cultural values of caregiving at home | Awareness still limited, stigma remains a challenge, access to specialized care varies | [Link](https://uaetimes.ae/living-with-alzheimers-how-patients-and-families-in-the-uae-are-coping/) |
| **41** | **News Releases - Local Media Sources/**  **Newspapers** | UAE | Family and caregiver experiences | Gulf News | September 21 2023 | Community support and caregiver narratives | Available | People living with Alzheimer’s in the UAE and their families | The article shares lived experiences of families coping with dementia, highlights available services including memory clinics and home care support, and discusses challenges in daily care | Family involvement and respect for elders emphasized; stigma and silence around the diagnosis mentioned | Limited awareness, emotional toll on caregivers, need for more structured services and early diagnosis | [Link](https://gulfnews.com/uae/health/living-with-alzheimers-how-patients-families-in-uae-cope-1.96559506) |
| **42** | **Information articles - Private Home Healthcare Service Providers** | UAE | Dementia care access for expats (Information) | Enayati Home Healthcare | Mar-24 | Private home healthcare services for dementia | Available (Enayati provides home-based dementia care) | Primarily expat families in the UAE | Discusses the growing concern about dementia care for expatriates in the UAE, highlights how home healthcare services like Enayati aim to fill gaps, particularly for non-Emirati residents, emphasizes home-based caregiving and customized dementia support. | Cultural and family caregiving patterns differ between Emiratis and expats. Expats often lack extended family networks, increasing reliance on private services. | Limited government support for expatriates, especially long-term dementia care. Unclear pathways for expats to access affordable and specialized services. Dependency on private agencies. | [Link](https://enayati.ae/dementia-dilemma-for-expats/) |
| **43** | **Care Services - Healthcare Institutions/**  **Hospitals** | UAE | Clinical dementia care | Burjeel Hospital | Not stated (active webpage) | Specialized outpatient service | Available | Individuals with Alzheimer’s disease and their families | Provides information about Alzheimer’s disease, signs and symptoms, stages, and treatment options. Describes multi-modal management including medication and lifestyle changes. | No direct mention, though family involvement is implied in patient management | No detail on long-term care, caregiver support, or integration with national dementia strategies. | [Link](https://burjeel.com/alzheimers-disease/) |
| **44** | **Care Services - Healthcare Institutions/**  **Hospitals** | UAE | Clinical dementia care | King’s College Hospital Dubai | Not stated (active webpage) | Neurology clinic with Alzheimer’s care | Available | Individuals with Alzheimer’s disease and their families | Describes causes, risk factors, and symptoms of Alzheimer’s disease. Offers diagnostic services and personalized treatment plans including medications and supportive therapies. | Not explicitly discussed | No mention of caregiver training, long-term care pathways, or cultural tailoring of services. | [Link](https://kingscollegehospitaldubai.com/service/neurology/alzheimers-disease/) |
| **45** | **Care Services - Healthcare Institutions/**  **Hospitals** | UAE | Geriatric & elderly care services | Northwest Clinic | Not stated (active webpage) | Geriatric outpatient services | Available | Older adults, particularly those with chronic conditions or cognitive decline | Multidisciplinary approach including preventive care, chronic illness management, fall prevention, cognitive assessments, and support for memory-related conditions | Mentions family involvement as part of holistic care but does not expand on cultural or religious aspects | No explicit gaps or system-level challenges mentioned | [Link](https://northwestclinic.org/what-are-geriatric-services-in-dubai-and-why-are-they-important/) |
| **46** | **Care Services - Healthcare Institutions/**  **Hospitals** | UAE | Dementia & Alzheimer’s care | German Neuroscience Center (GNC) Dubai | Not stated (active webpage) | Specialized psychiatric and neurological outpatient services for dementia and Alzheimer’s | Available | Adults and elderly individuals with Alzheimer’s, dementia, or related neurocognitive disorders | Offers comprehensive diagnosis and treatment including neuropsychological assessments, medication management, and long-term outpatient care. Multidisciplinary team includes neurologists, psychiatrists, and psychologists | No specific mention of cultural, religious, or family involvement on the webpage | No explicit challenges or systemic gaps mentioned | [Link](https://www.gncdubai.com/disease/psychiatric-alzheimer-dementia/?tbjp=380&utm_campaign=alzheimer&gad_source=1&gad_campaignid=184327384&gclid=Cj0KCQjwvajDBhCNARIsAEE29WqYj0rGmnrlz7ZCLuSQUQC2lJqsV4F8R6yWXBzEweQ9WX23CbdDEZsaAgQgEALw_wcB) |
| **47** | **Care Services - Healthcare Institutions/**  **Hospitals** | UAE | Dementia & Alzheimer’s care | Cleveland Clinic Abu Dhabi | Not stated (active webpage) | Educational support for caregivers and clinical care for Alzheimer’s patients | Available | Persons diagnosed with Alzheimer’s disease and their family caregivers | Describes Alzheimer’s progression and caregiving responsibilities. Emphasizes caregiver roles in safety, nutrition, medication, and emotional support. Encourages early diagnosis and routine care. Highlights support for caregivers’ well-being. | Family’s role is central; reflects cultural expectation of family caregiving, but no explicit mention of religious or cultural tailoring | Notes caregiver stress and burnout as challenges; urges caregivers to seek professional guidance | [Link](https://www.clevelandclinicabudhabi.ae/en/health-hub/health-resource/diseases-and-conditions/alzheimers-disease-and-caregivers-role) |
| **48** | **Care Services - Healthcare Institutions/**  **Hospitals** | UAE | Memory and cognitive care | Canadian Medical Center (CMC) Dubai | Not stated (active webpage) | Outpatient Memory Clinic services | Available | Adults with memory complaints, dementia, or cognitive decline | Offers memory assessments, neurological consultations, imaging, and neuropsychological testing. Multidisciplinary team includes neurologists, psychiatrists, and psychologists. Early detection and diagnosis emphasized. | No explicit reference to cultural or religious aspects. Family role implied in the diagnostic and care process. | Lack of mention of long-term follow-up, care navigation, or support for caregivers | [Link](https://cmcdubai.ae/solution/memory-clinic/) |
| **49** | **Care Services - Healthcare Institutions/**  **Hospitals** | UAE | Geriatric and dementia care | Mediclinic City Hospital (Dubai) | Not stated (active webpage) | Geriatric outpatient services and Memory Clinic | Available | Older adults, especially those with memory concerns, multiple chronic conditions, or complex care needs | Multidisciplinary assessment and management of memory disorders (e.g., Alzheimer's, other dementias), cognitive screening, early diagnosis, holistic geriatric care (physical, psychological, functional, and social), fall risk and frailty assessment | No specific cultural or religious tailoring mentioned; family involvement is implied in care process but not emphasized | Lack of detailed care pathways or mention of coordination with community/home-based dementia support; cultural sensitivity not explicitly addressed | [Link](geriatrihttps://www.mediclinic.ae/en/city-hospital/services-and-specialities/geriatrics.html%20cs,%20Memory%20Clinic) |
| **50** | **Care Services - Healthcare Institutions/**  **Hospitals** | UAE | Clinical diagnosis and treatment of dementia | Dubai Royal Hospital Clinic (DRHC) | Not stated (active webpage) | Neurology clinic offering dementia care | Available | Older adults with memory and cognitive concerns | Provides evaluation and treatment for patients with Alzheimer’s and other types of dementia. Focus on neurology-led diagnosis, cognitive testing, and personalized treatment plans. Multidisciplinary support hinted at but not expanded. | No direct mention of cultural, religious, or family context in care provision | Lack of detailed info on long-term care pathways, non-pharmacological interventions, or caregiver involvement. | [Link](https://www.drhc.ae/dementia/neurology-clinic-dubai) |
| **51** | **Care Services - Healthcare Institutions/**  **Hospitals** | UAE | Clinical neurology – Alzheimer’s care | Novomed | Not stated (active webpage) | Specialized neurology clinic | Available | People with Alzheimer’s disease | Provides diagnostic evaluations, medication management, and symptom-focused treatment for Alzheimer’s disease. Emphasizes early diagnosis and personalized treatment plans. | No mention of specific cultural or family-based adaptations | Does not mention interdisciplinary collaboration or family support services; cultural integration is lacking. | [Link](https://www.novomed.com/services/specialized-clinics/neurology/alzheimers-disease/) |
| **52** | **Care Services - Healthcare Institutions/**  **Hospitals** | UAE | Neurological care and services | Fakeeh University Hospital | Not stated (active webpage) | General neurology and brain health | Not available (No explicit mention of dementia) | Patients with neurological disorders (e.g., stroke, epilepsy, Parkinson’s, migraines) | The hospital offers comprehensive neurological care including diagnostics, treatment, and neurophysiology services. However, the site does not explicitly mention dementia or Alzheimer’s-related services. | Not mentioned | No mention of cognitive disorders or dementia-specific care; lacks geriatric neurology or memory-focused services. | [Link](https://www.fuh.care/specialties/brain-nervous-system-neurology) |
| **53** | **Care Services - Healthcare Institutions/**  **Hospitals** | UAE | Dementia clinical services | Emirates Hospitals | Not stated (active webpage) | Memory Clinic | Available | Adults experiencing memory loss or cognitive decline | Offers diagnosis and management of dementia through its memory clinic. Services include early detection, treatment plans, and support for cognitive disorders, focusing on improving quality of life and delaying progression of symptoms. | Not explicitly mentioned | No information on integration with caregiver training or community-based dementia support systems | [Link](https://emirateshospitals.ae/services/neurology/memory-clinic/dementia/) |
| **54** | **Care Services - Healthcare Institutions/**  **Hospitals** | UAE | Clinical dementia educational article | American Centre for Psychiatry and Neurology | Not stated (active webpage) | Health education and clinical services | Available | Individuals with or at risk of Alzheimer’s disease | Provides an overview of Alzheimer’s disease, including symptoms, risk factors, and progression. Highlights the importance of early diagnosis and medical intervention. Promotes clinical support available at the centre but focuses mostly on public awareness through education. | Not mentioned | No detailed service breakdown, no reference to culturally informed approaches or community/family support programs. | [Link](https://americancenteruae.com/health-education/alzheimers-disease-ad/) |
| **55** | **Care Services - Healthcare Institutions/**  **Hospitals** | UAE | Clinical dementia care | Brain Hub UAE | Not stated (active webpage) | Alzheimer’s Clinic | Available | Older adults with Alzheimer’s and related cognitive disorders | Provides neurological consultation, neuropsychological assessments, and tailored treatment plans. Focus on early diagnosis, personalized cognitive therapies, and ongoing monitoring. | Not mentioned | No mention of integration with national dementia strategy, caregiver involvement, or cultural adaptation. | [Link](https://brainhubuae.com/clinics/alzheimer-clinic-in-dubai-uae/) |
| **56** | **Care Services - Healthcare Institutions/**  **Hospitals** | UAE | Cognitive rehabilitation services | Brainnovation | Not stated | Non-medical cognitive training | Available | Individuals with Alzheimer’s or other dementias; families seeking non-drug therapy options | Offers brain training programs using neuroplasticity principles to support memory, focus, reasoning, and attention, describes benefits for older adults with dementia and Alzheimer’s, and mentions customized brain stimulation sessions. | Not explicitly mentioned | Lacks discussion of clinical diagnosis, integration with broader care models, or cultural tailoring. | [Link](https://brainnovation.ae/alzheimer-and-dementia-treatment-dubai/) |
| **57** | **Care Services - Healthcare Institutions/Hospitals** | UAE | Occupational therapy for dementia | QE Enrichment Centre | Not stated (active webpage) | Occupational therapy interventions | Available | Adults with dementia, especially in early to mid-stages | Offers personalized occupational therapy sessions to help individuals with dementia maintain independence in daily activities, focuses on cognitive stimulation, memory strategies, routine building, and sensory integration. | No explicit mention of cultural or religious tailoring; family role implied but not described | Lacks detail on coordination with medical or long-term care providers, and absence of culturally grounded approaches. | [Link](https://qenrichment.com/occupational-therapy-for-adults/dementia-ot/) |
| **58** | **Community Initiatives - International Organizations** | UAE | Awareness, Support Services | 4get-me-not Alzheimer’s Organization (via ADI) | Not stated (active webpage) | NGO-run dementia awareness and support | Available | Persons with dementia, families, and caregivers | UAE-based Alzheimer’s organization focused on dementia awareness, family support, and advocacy, offers workshops, training, and public campaigns, member of Alzheimer’s Disease International (ADI). | Emphasizes family-centred support and culturally relevant awareness materials. | No official national recognition or integration into government dementia care strategy noted. | [Link](https://www.alzint.org/member/4get-me-not-alzheimers-organization/) |
| **59** | **Private Caregiver Training Centres** | UAE | Caregivers training | NLP Tech Training Centre, Dubai | Not stated (active webpage) | Caregiver Training Course in Dubai - open enrolment, short-term course, offered regularly | Available | Caregivers, family members | A Professional caregiver certification, covers elderly and dementia care basics, emphasis on daily living support, safety, mobility, hygiene, and nutrition - Includes theoretical and practical components | Not explicitly addressed, but course is UAE-based and may include implicit adaptation to local norms | Highlights the emerging demand for structured caregiver education; could inform future national standards or training regulations for informal or paid home care | [Link](https://nlptechforma.com/medical-courses/caregiver-training-course-in-dubai/) |
| **60** | **Private Caregiver Training Centres** | UAE | Dementia education and aged care | UAE Montessori | Not stated (active webpage) | Training and caregiving approach resource | Available (in educational/training form) | Caregivers and aged individuals | Offers a brief overview of dementia and aged care principles, emphasizing person-centred approaches, advocates compassionate caregiving and basic knowledge sharing. | No UAE-specific cultural or religious tailoring explicitly mentioned | Lacks depth on care models, clinical pathways, or structured training frameworks; no service delivery described. | [Link](https://uaemontessori.com/dementia-and-aged-care/) |
| **61** | **Private Caregiver Training Centres** | UAE | Training and workforce education | Samson Training Center | Not stated (active webpage) | Introduction to Dementia – Training Course | Available | General public, aspiring caregivers, healthcare staff | A foundational course providing an overview of dementia, common symptoms, communication strategies, and caregiving techniques. Offered in the UAE with certification. | Not reported | No formal integration into national dementia training pathways noted | [Link](https://www.samsontraining.ae/courses/introduction-to-dementia/) |
