# Supplementary material for: Dementia care in the United Arab Emirates: an environmental scan of services, policy, workforce, and caregiving contexts
Source: BMC Health Serv Res. 2026 May 26;26:951. doi: 10.1186/s12913-026-14598-9 (PMC13356796; doi:10.1186/s12913-026-14598-9)
Supplement: Supplementary file 3 [file 12913_2026_14598_MOESM3_ESM.docx]

**Interview with Geriatrician 1**

December 28, 2023

**Interviewer:**

So, what do you do? What does your position involve?

**Interviewee:**

So we are basically the people in charge in the hospital for looking after people with dementia. **Interviewer:**
OK, good. How long have you been associated with this organization? **Interviewee:**
This is my ninth year. Yeah. **Interviewer:**
OK. What proportion of the people under your care are diagnosed with dementia? **Interviewee:**
Umm, currently as of now as of today, all of our patients are demented. **Interviewer:**
Uh, OK. And what would be the estimated number for that?

**Interviewee:**
We handle around 10, 11 to 50 patients a day. **Interviewer:**
OK, so, in the outpatient clinic. **Interviewee:**
No, inpatient.  **Interviewer:**
Inpatient. In the outpatient clinic, it varies.

**Interviewee:**
So there we have, I would say around 60 to 75% of them have some form of dementia, some form of cognitive decline. **Interviewer:**
OK. And once they are admitted, what's the average number of days or weeks that they stay with you under your care?

**Interviewee:**

Well, there isn’t a specific number, but I would say anywhere around 10 to 14 days. **Interviewer:**
OK. And what are the reasons that they get admitted in the 1st place?

**Interviewer**:
Do they carry the diagnosis [of dementia] when they come to see you or you do the diagnosis once they are admitted? And what are the reasons that they get admitted?

**Interviewee:**

No, you have both, but most often they come in with the diagnosis. They already have an established diagnosis of dementia and then they have some of these comorbidities that happen. They could have pneumonia at the community acquired pneumonia, aspiration pneumonia, urinary tract infection, strokes, or increasing confusion, agitation, not eating well, enough, lack of appetite, dehydration, such complications. Rarely we see a cardiac event, maybe a heart attack or heart failure. Yep. **Interviewer:**
OK. And what is the demographic of your patient population if you don't mind me asking?
Are they Emiratis, the majority, or do you see, I mean, we live in such multicultural city. What's the demographics like when it comes to your patient population? **Interviewee:**
We have 80% of our patients are Emiratis and maybe around 15 to 20% is other nationalities. **Interviewer:**
OK, so as you know, there aren't yet any kind of epidemiological studies done on the prevalence and incidence rates of dementia in the UAE. So the exact number of Emiratis diagnosed and living with dementia is still unknown. And the few articles that are out there really underestimate that number. In your estimates, and based on your experience working in the city, what proportion of the population that you monitor, do you believe is living with dementia? **Interviewee:**
We know not everyone gets the diagnosis even though they live with it. **Interviewee:**
Yes, yes. But well, generally here the population and the Emirati population have a longer lifespan compared to other countries, for instance, in the average lifespan. **Interviewee:**
I think here from my experiences, the number of people above 75 who has significant cognitive decline is anywhere around 70%-ish because that's what…that's the worst number of people in that age group who tend to have cognitive decline. Family would not notice it, but they come in for something else. They come in with a fall. They come with a fracture and come in for assessment, and as part of the assessment, we pick up these augmentive issues. So I think the numbers are quite high.No, it's just that we don't have enough…enough data and research to show us that…that the numbers are high. We have a significant amount of people here who are cognitively compromised. **Interviewer:**
So I appreciate you for mentioning that and that's one of the reasons I reached out to you is because stakeholders such as yourself are very important in getting your voices to be heard. I mean, these interviews that I'm collecting from are going into an environmental scan of dementia care practices and services in the UAE. So your input is is extremely valuable. One of the the things that I noticed once I this research project is the lack of peer reviewed articles in, you know, with regards to dementia. **Interviewee:**
Thank you. **Interviewer:**
What are your interests like when it comes to research? **Interviewee:**
Yes. I believe research is very important.  **Interviewer:**
We know that topics such as neurodevelopmental, neurological, neuropsychiatric disorders.
such as epilepsy, multiple sclerosis - these are widely covered in the literature. Dementia is an up and coming topic, and if I may ask, as a qualified practitioner, what are the reasons that…what's stopping you from pursuing research? Is it sheer lack of time? Is it lack of interest or resources?

**Interviewee:**
Well, I think I mean I I can't comment about what others, but personally I am quite…quite keen on research and finding out information. But The thing is that there is a distinct lack of resources with regard to research…we are very tight strapped when it comes to time. I need to find time for collecting the data. We're looking at a sample size of around 1800 patients, so skimming through the files of 1800 patients, collecting data is very exhaustive. Yes. We don't have research associates or anything of that sort. So what happens is that all of this is on the investigators and out of our clinical time.
It's very difficult to find time to complete these things. And what is normally meant to to be done in six months takes a long time. So what happens is exhaustive. It's cumbersome. **Interviewer:**
Umm. **Interviewee:**
I think the scientific community in this part [of the world] is not yet tuned to the fact that, you know, we need to start doing more research. We need to look into the data that we have in the local population.
What can we derive out of it rather than blindly going by what the global standards are, we'll need to have local numbers. **Interviewer:**
Yeah, very good. I hope it works out and you know people start. Clinicians need to start engaging in more research. **Interviewee:**
Exactly. **Interviewer:**
Yeah. Umm my next question is, with regards to your definition of what dementia is as a degenerative condition. What is your definition of dementia as a degenerative condition? **Interviewee:**
Dementia is an umbrella term that comes in with, you know, numerous cognitive domains. So compromising either one or many of the cognitive domains itself is what you mean shares. **Interviewer:**
How would you describe the type of care you provide to people with dementia, both in at outpatient and inpatient capacity? What's a typical day like when it comes to caregiving, providing care for a patient with dementia? **Interviewee:**
Well, this being basically an in-hospital unit and a geriatric unit. Uh, primarily, what happens is that we would have a patient being called in by the trauma team saying that they've had somebody who's coming in with a hip fracture following a fall. So we assess the patients. So as a part of our routine assessment, we do have this tool called the Comprehensive Geriatric Assessment, wherein we assess multiple domains, cognitive function mode, function status, comorbid medications, nutrition.
All of these things, and then we prepare a problem list of what are the issues that are currently bothering this patient and what is preventing them from achieving maximum functional status.
And we go ahead tackling either of these things. So one of them is most often dementia of the problem here is that they have come in with an acute stress or an acute illness, and they are obviously going to be demented. So it is going to be difficult task to…to kind of delineate what is the dementia focus out of this delirium because you really can't do a full dementia assessment while the patient is in delirium state. So you need to wait for this delirium to come down. So then we continue.
So we have, we follow this patient on a regular basis and see when the delirium comes down, delirium settles down, we repeat our assessments to actually ascertain what level of cognitive decline this patient has got. Unfortunately, we don't have other…other auxiliary methods like we don't have a clinical psychologist on board. We don't have other support personnel, so our options here are we will directly start off with medications to aim, you know, prevent further degeneration of the condition…to arrest the progression or reduce the rate of progression of dementia. Meanwhile, we consult the family about the diagnosis. What is it going forward? What are other things that we'll have to anticipate? What are the problems that we're expecting and how do you tackle these problems with regard to eating, sleeping, management of functions, status management, and what are the problems going to be encountered there? How do you manage them? How do you make sure that the patient is not get delirious and how an acute illness can actually uh, actually, you know, hasten the progressive dementia. So we'll be counseling the family about their whole support system, what to expect and what is coming up and how the medication is going to be working, how the medications might not work and what to expect with the medications. And we continue to follow them up on an outpatient basis. Once in 4 to 8 weeks, and once we've achieved a level of stability, then once since every six months. **Interviewer:**
Ok. Generally speaking, how do family members receive the news of a dementia diagnosis? **Interviewee:**
Here they generally are not surprised, [it’s as if] they were anticipating it because I think inherently the Emirati population beyond a certain age, the level of dementia is quite prevalent. But people don't understand the word dementia. You tell the word Alzheimer’s, then they understand. So Alzheimer’s is something that the local population understands quite well and they've had one or many family members with Alzheimer’s and therefore they tend to relate to the diagnosis really well. They're more accepting, but the diagnosis is something that we already know, [it’s just that] we just reaffirm that it is one of these things. **Interviewer:**
I mean, knowing what, knowing that it's Alzheimer’s is very different from knowing what Alzheimer's is actually, do you in your experience that? **Interviewee:**
Yes, yes. **Interviewer:**
Are they actually aware of? You know what comes with that kind of diagnosis, for example, about what dementia is and that it has different types? What is their reception like? Yes, they recognize.
What's Alzheimer's is, but do they go and modify things to suit the patient, or do they continue as things were?  **Interviewee:**
Most most of the time they do, they do.They do make changes with, you know, once they…they accept the fact that, yes, this is something we do. Tell them that this is. This looks like Alzheimer's, or this is not Alzheimer's. This is another case waiting to turn into Alzheimer's, and sometimes, you know, based on those details in the process, how fast does this disease progress. And Alzheimer’s is a very uh, slow progressive disease. So we prime them for that. They understand the repercussions that come with it and very, very often I think extremely rarely we've seen otherwise, but they, they do adapt and make changes according to what works and what is best for the patient. And I think the social fabric is excellent in terms of that, and also what happens is that sometimes when they need to be when they will take off from work, the government simply gives them leaves and things that facilitates taking care of an ailing person. So yes, they are very accommodative of the needs and they do the need for when it comes to. **Interviewer:**

OK. Umm. I'm not sure if if you're part of that group, but there is an Alzheimer's support group that is open to the public [on WhatsApp]. **Interviewee:**
Umm. **Interviewer:**
I have been umm added it to that group and I've been following the trend, and one of the patterns that I see is, you know, the trend of outsourcing care to services to provide, for example nurses on call. From your experience, who are the primary caregivers, caregivers for patients with dementia, is it family members such as spouses, daughters, sons or do they hire help as part of, you know, the caregiving plan? **Interviewer:**
What kind of trend do you see in your clinical practice? **Interviewee:**
Uh, I'd say hired help is…is I think 95%. It's always an external agency, one of the nursing agencies. They have their people coming in, they are often the primary caregivers. Very rarely do you see in that in non-Emiratis. [In the case of non-Emiratis]. It's often the spouse, any of the Indian sub-content crowds, 99% it's a spouse or one of the children, but in in the case of Emiratis, the trend is most often it is, it is hired help.

**Interviewer:**

And would you say that they know how to provide care for a person living with dementia? What is their knowledge like? Both family members and hired help?

**Interviewee:**

Knowledge is lacking, generally. I would say with everyone.  **Interviewer:**
So would you reckon that targeting, you know, these care services on the agencies in terms of providing training, establishing you know a standard strategy or framework in caregiving that would bring more positive results? Or do you think even training the family members? Which one would you say would bring a difference in the quality of life and the and the life satisfaction for patients living with dementia? **Interviewee:**
We have to choose between them. Yes, the agencies, definitely. But ideally I think we need to have training on both sides because the family also needs to understand what are the standards, what are we expecting, what are the targets, what are the interventions? Because sometimes the interventions may not make sense to them. For example, one of the commonest, the commonest recommendations we tell people is that to not let them sleep in the morning. So we want to establish this circuit so then we want them to be sleeping only the night we need to be stimulating them during the mornings. But doesn't misconception that he hasn't slept in the night. The patient has not slept in the night, so let him sleep. So when we tell them this, initially the family is quite surprised…he's not slept [the night before], so let him sleep, they say. You can't let him do that, because if you do that then that becomes rhythm. That becomes a pattern. He's going to be sleeping like this. That will hasten his delirious spell. Delirium. You know the slow progression of dementia. So that is something, it's…even though the hired help tries to make this change, the family has to be on board with the plan because eventually they make the call how they want. So, if so many working with the agencies or the hired help alone will not be enough, we will have to have all of them on board. **Interviewer:**
OK, from you may not know this directly, but when they hire a help from the agencies, is it on a daily basis? In other words, every day the person would get a different nurse. Or is, is there a plan where they stick to one nurse so that she or he gets familiar with the patient and they know their routine? **Interviewee:**
It’s always the same. Same nurses. They have two nurses generally and they work in 12 hour shifts.
I think it's when one goes on leave they have somebody replacing them. Otherwise it's always the same. The same two people. **Interviewer:**
That's very insightful. In terms of agitation, aggression, or any type of psychosis or psychotic behavior, how is it generally handled in the clinical environment when a person shows those symptoms alongside their dementia diagnosis, what is the first form of treatment that you provide to them? **Interviewee:**
Do you mean to say in the acute situation or on the long term? You know when they go back and generally we'll find, we'll have to find out if there is any trigger to this agitation. Is there pain?
Is he constipated? Any of these things?Is that an ongoing infection? Is there a trigger for this, if there isn't an identifiable trigger, there isn't something that is correctable then you have to push this into being agitation associated with dementia and then the question arises, how manageable is it?
If it is manageable, if it is not problematic, if it is not inducing self-harm or harm to other people, we would generally let it pass. But if it comes to either self-harm or harming other people, then we would be using restraints, chemical restraints, or physical restraints. **Interviewer:**
Umm. And the use of antipsychotic medication. How common is that? **Interviewee:**
Quite common, quite common. **Interviewer:**
OK, OK. And why is it common, if I may ask? **Interviewee:**
Because. Because what happens is that now physical restraints generally makes them more aggressive. Most of them have other comorbid illnesses have cardiac conditions, so persistent agitation is a strain on the primary care with the strain on the patient's health as well. Well, and it is very difficult when they have these upsurges to manage them. So there's a lot of stress around the patient and the caregiving that needs to be alleviated. So most often, yes, we would be using antipsychotics. **Interviewer:**
OK. And what proportion of your patient, would you say, is on antipsychotic medication long term? **Interviewee:**
Agitation with dementia patients at least around 60 to 70%. **Interviewer:**
What about aggression? Physical aggression. **Interviewee:**
See same. **Interviewer:**
Oh. Ok. What about in acute situation? **Interviewee:**
Probably even more, probably even more if it's aggressive, if it's quite aggressive, probably even more than that. **Interviewer:**
OK. What about in acute cases? What is the first form of treatment? **Interviewee:**
Uh. In acute spells of agitation, it's often antipsychotic haloperidol injection, while in the hospital. **Interviewer:**
Is it a one-off? **Interviewee:**
One off, yes, no, not for long-term unless the patient requires it. And if the patient is requiring a haloperidol every day, and then probably, then we'll consider having something regularly, because now the frequency of usage is quite high. Then we would consider something regular. **Interviewer:**
OK. Umm, there is a concept known as person centered care. I wanted to know. First of all, what do you know about it and how much of it is being applied in your practice? For example, when you advise, consult, and advise the patient as well as family members. **Interviewee:**
Mm-hmm. **Interviewer:**
Umm, what kind of? What type of care do you do you recommend to them? And is person centered care one of them? I wanted to hear your thoughts on person centered care and the whole idea behind it. **Interviewee:**
I don't know what you mean here by person centered care, but generally based on how it is, generally a personalized care, that happens. But I don't know if there are any…any particular guidelines or any particular policies that are followed about it, but yeah. **Interviewer:**
OK, OK. So basically, you know, I'm seeing the person as a person first, and not their diagnosis. The importance of recognizing the person as a person with needs and preferences. This is the whole the fundamental idea that is behind it is that, and also personalizing the care, acknowledging that no two patients with the same diagnosis are the same and cannot be the same. This is generally…the general guiding principle behind person centered care. Would you say that this is the type of care that's implemented in this part of the world? **Interviewee:**
Yes, yes, it always is because uh, supposing somebody has a diagnosis of dementia and it is very important to have that personalization, because otherwise it is going to be difficult to managing the individual going forward because based on what their preferences, their baseline preferences what they were and what are their likes, dislikes. So you need to be within that system of identifying the individual and the diagnosis comes later because you can't treat it only as a diagnosis, so that is always there. **Interviewer:**
Umm, OK. Describe a good day to me and then a challenging day in your caregiving activities. What's a good day and what's a bad day like?  **Interviewee:**
A good day is when none of our patients that are agitated or you know that they're not having…there isn't a problem with respect to their caregiving. But that's very rare. Very often we do have because this being a geriatric unit, they are in the hospital for an acute illness. Obviously there is going to be an issue. There are going to be issues to be dealt with, varied, patterns of delirium and how the family takes it, and how we convince them on how do we manage this going forward and what are the problems and why is this happening? Because it's not always that, you know, why is he behaving like this? Why is this happening? So consulting the family each time something goes off is a cumbersome task until they come to that stage of acceptance. Realize, OK, this is how it is going forward, but it's so difficult. Very rarely do we have good days, but. **Interviewer:**
It's the nature of the work.
You do? **Interviewee:**
Yes, exactly.

**Interviewer:**
Umm. Would you say that there are challenges in language and communication? When they show these kind of behavioral changes and you're…you're not managing…or able to control…in your practice, you mentioned majority of the patients you see are Arabic speakers, for example. **Interviewee:**
Yes. **Interviewer:**
Would you say there is a disconnect between your caregiving and the care recipient because of cultural differences or language? The differences, how do you manage that? **Interviewee:**
Yes, that that is often a concern, but we are a team, so primarily, we work as a team.
So if it's most of the patients they speak some level of English or Urdu, say 20 or 30% do, but the rest, absolutely no other languages but Arabic. **Interviewer:**
Umm. **Interviewee:**
So [as part of the team] we have colleagues who handle certain patients exclusively, from the speaking aspect, counseling, assessing, and then we discuss the case and we [prepare] a management plan. **Interviewer:**
OK. My next question is, regarding staff who do not share culture and language with their patient population, is there formal training provided? You know in…in picking up at least common phrases that will create that medium of communication between them and their patients? I’ve asked this question to others as well, and the answer is…mostly the answer is no. And physicians do not receive any type of training to manage this communication barrier. I think living in this part of the world where English is is spoken by most people. We get lazy about learning a new language because in English is easily accessible.  **Interviewee:**
Well, it depends on the nature of work. Working in [government] hospitals, I think it is important to have formal training in Arabic because some level of proficiency in Arabic [is needed]. But if you're working outside the government structure, probably not. Because I have colleagues working in, say, private hospitals, and uh, they deal with very little Arabic speaking or Emirati patients. So the vast majority of patients [they deal] with are from the Indian subcontinent or from the other places, so probably not for them. You can apply this universally but depends on your place of work. I think it has to be an institutional thing rather than a central licensing requirement. **Interviewer:**
OK. Would you say, your patients with dementia are involved in making decisions that concerns them both with the care staff and family members? So, for example, diet preferences, clothing, preferences, time was sleeping time, waking up, managing their finances, taking medications, capacity, tube feeding, end of life care, and generally life sustaining treatment. To what extent are they involved in making decisions for themselves? **Interviewee:**
Uh. There is a very little number of patients who actually make decisions for themselves. I would say 10 to 15% most often it's the family that takes decisions on the patient's behalf. It is an implied thing that...that is different, you know it is, it is to be taken by the family and that's…that's very opposite to what practices are when you're comparing it in places like Europe because until they have a certain level of independence, they have a decision making capacity. They go on to take decisions regarding their health, finances, their diet and these things, but this part of the world, Indian subcontinent, the Arab world, I think it is, it is an implied thing that the family takes over these decisions and they act. **Interviewer:**
That's. Sorry to interrupt. Is that that's once the diagnosis is made, regardless of the severity of their condition? And is it forced…or is there a form of resistance that patients should? **Interviewee**:

Yes. No, no, I think, I think it's, it's culturally accepted in this part of the world that, you know, it comes with the diagnosis. **Interviewer:**
It comes with it. OK. What are your thoughts [on that]? **Interviewee:**
No, I don't. No, I don't. I'm…unless, unless the patient is severely compromised, they have lost their decision-making capacity. I believe that it is up to the patient, the individual, to take certain calls about them, unless there is, there is going to be an obvious hum, but I would not. I wouldn't want that to be done for me. I would want to make my decisions until I reach the stage where I can't anyway. **Interviewer:**
Umm do you think not being given that freedom to make decisions impact their quality of life, their life satisfaction? **Interviewee:**
To some people, yes, to some, some individuals definitely yes. But as I said, it's an acceptable cultural norm here in this part of the world and in Asian countries. So, I think they've come to realize that that's how it is so. **Interviewer:**
So you're talking about the person, the patients. From their perspective, they accept it? **Interviewee:**
Yes. **Interviewer:**
Yeah.
Do you see signs of unhappiness? Dissatisfaction. **Interviewee:**
Sometimes, sometimes yes. **Interviewer:**
OK. Do you have any strategies you use to help you carry out your caregiving activities? Manage “challenging behaviors” or emotional challenges. For example, in challenging situations, are there strategies other than going the biomedical way? Are there some strategies that you utilize if, if so, can you share? **Interviewee:**
Very often, as I said, did you mean prophylaxis is something we try and implement very strictly here, for example. Try and establish sleep rhythm. We're trying to, you know, continue to do activities to stimulate them either visually or physically during the day, switching on the lights and having the curtains open if possible, have them moved to a corner side bed with a window access that they can see it's day and night. And if it's possible, if the patient is clinically stable, try and move them to a room instead of a general ward, so that you know what is always noisy and right and busy with activity. So they move into quiet surrounding, so all of these measures are management of pain and checking they have got pain, is there any pain, making sure they are passing stool regularly. All of these things in hospital, I think that's very important and because they are in here for an acute illness, they are going to be delirious. And yeah, in management of dementia, it is going to be based on how well we manage their daily routines, so these strategies are universally applied here for our patients but otherwise it varies from patient to patient. **Interviewer:**
Can you think of a time when you didn't know how to handle a caregiving situation? And what did you do? **Interviewee:**
Umm. Offhand, I don't. I can't recall something, but yes, we do have situations where we really don't know how we're going to be managing it, but I can't. I can't think of an incident that has happened to recent past. **Interviewer:**
Umm. OK. We're reaching to the last couple of questions now. Umm, I mean the concept of care, home permanent care homes do not exist because the culture encourages family care and you know, family to take care of their own, their loved ones. But to your awareness, are there such facilities in and around Dubai? Are you aware of an organization or an institution that provides permanent dementia care? **Interviewee:**
Specifically for dementia, I don't think there are institutions. There are some institutions in Abu Dhabi, some private institutions that basically look after patients of all, for all scope, basically for patients [who are] quite advanced, more of vegetative state patients rather than exclusively for dementia, exclusively for dementia. I don't think there's anything anyway. **Interviewer:**
Would you encourage something like that to be established, what are your thoughts, hmm? **Interviewee:**
As a permanent caregiving option. Umm, it depends on what the patient and the family wants.
I think as is, as you said, the Emirati culture is not pro such a such a setup, but I think that might be of some benefit because it would be an exclusive center for dementia where professionals are going to be trained in this and there's going to be a more uniform way of…uniformity in caregiving rather than the heterogeneous way…it would be managed by the family and their social environments.
It’d also help rather than a permanent thing. I would [also] suggest something like a respite care setup where you know at least the primary caregivers have some respite from the constant…this caregiving for a dementia patient is very exhausting situation…you're looking at for that for 10 years, 15 years, 20 years. **Interviewer:**
Umm. **Interviewee:**
Something that wears down the primary caregiver. So if the primary caregiver is a family because they can't change, but the higher agency after some time, they will take it breaking the chains, the people, so they get a break at some point of time. If it's a primary care, if it's a family member, they're going to be completely exhausted in five or six years. The respite care setup, like a daycare center.
Kind of setup for those are the things I think will have good impact, social impact for dementia patients. Long term care, permanent care, maybe not. **Interviewer:**
Maybe because they're already outsourcing that type of care? **Interviewee:**
Exactly. **Interviewer:**
Yeah. Respite care. I mean, I agree with you in cases where it's…where the primary caregivers are family members. But we're seeing here the pattern is outsourcing that care to agencies. **Interviewee:**
Yes. **Interviewer:**
So I don't think families are greatly impacted as much as we think they are. Would you agree? **Interviewee:**
You, but that is that is, that is for the Emirati, Emirati subset. For the non-Emirati subset, again, the caregivers are always the family members, so yeah. **Interviewer:**
Absolutely, absolutely. But, what is the proportion of older people living here, then? Since, here the trend is once you retire, you go back to your country of origin. Right? **Interviewee:**
Yes. **Interviewer:**
So are you saying that you are seeing lots of patients in, in the older age group that are from other countries, from outside? **Interviewee:**

Yes, off of late, there has been a change, you know, yes, just yesterday I had this French gentleman who has lived all his life in France and then now he's retired and now he lives in Fujairah. So and he has got multiple comorbidities, he's got mild to moderate cognitive decline and…but he has no clue of how the healthcare system here works. So, there is change in in in the kind of people who coming in there are people like these who want to live their retirement lives in UAE. And so I think we'll have to have going forward maybe not now, but another five years, we're going to have to look at a good number of people who are older, retired, non-Emirati, requiring care. **Interviewer:**
So the need for establishing such facilities is, I think timely, then, well, I have one more question.

**Interviewee:**
Exactly, yes. Yeah, sure. **Interviewer:**
My question is, is there anything else you think is important that you haven't yet told that you would like to tell me? **Interviewee:**
Umm, my most…one point is the fact that if you can have big centres for elderly or dementia patients, you know something like a nursery for them they have an activity because most of the time with dementia or even without dementia, depression is a very strong, very, very strong, uh, condition.
Pretty common with the elderly here and that comes in mostly with isolation. Loneliness. Not having anybody to interact with in the mornings if it's locals or non-locals, the same, the same story applies everywhere. For all of these people. So, something of that sort that comes by I think will be transformative with regard to psychological care for people. I think in this part of the world.

**Interviewer:**
My last question or comment is inspired by what you told me earlier. It's the lack of resources when it comes to carrying out research. **Interviewee:**
Yeah. **Interviewer:**
Would you be interested in doing research, if time and resources allowed? **Interviewee:**
I'm more than more than open and more than welcome to that..to that idea. **Interviewer:**
Because, I mean the population, the numbers that you cited 1,800 patients, that's real data right there, and not everyone has access to data. **Interviewee**
I think it's very important. **Interviewer:**
Ok. Thank you. This was extremely informative and I really, really would like to thank you for your time and your valuable input. **Interviewee:**
Yeah. No problem. **Interviewer:**
Yeah, I hope to reach out to you. **Interviewee:**
So no problem. I'm very open to research but it's just that, you know, I'm crippled in more ways for that. **Interviewer:**
Absolutely. The time. **Interviewee:**
Yeah, yeah.

**Interviewer:**
Thank you so much. It's been amazing talking to you.

**Interviewee:**

Thank you so much.

**Interviewer:**
Bye bye.

**Interview with Geriatrician #2**

June 9, 2024

**Interviewer:**

Hello. OK. Tell me, what do you? In your position as a consultant geriatrician.

**Interviewee:**

I am. I work in geriatric clinic and also memory clinic. Uh, there we receive any one with any memory deficit. With early [stage] or advanced from the age of 60 and above. Usually we see a little bit more advanced cases of dementia. Uh, this is my job right now. Plus, home visits for all geriatric patients, especially those who are not ambulatory and cannot attend the clinic…Alzheimer’s or dementia patients at home. Those mainly with behavioural disturbances. **Interviewer:**
OK. And the home visits, are they part of your employment at the two centres? Or you do it privately? **Interviewee:**
No, no, it's related. Also part of our work is home care for elderly.

**Interviewer:**
OK. And how often does it happen? The home visits. **Interviewee:**

It's not less than three time per week. **Interviewer:**
OK. And do you do multiple patients at in one go or is it just one a day or maybe like a?

**Interviewee:**

The visits, we visit 4 cases. **Interviewer:**
Uh. Every day. **Interviewee:**

Yeah.

**Interviewer:**

How do you find it? How how do you find your schedule? Is it manageable? **Interviewee:**
Yes, it's a very busy schedule, but nowadays it would be…we have a very big waiting list.
Uh, to book appointment, you may need to wait for 60 days to book appointment in the memory clinic.
Uh, because we are also covering something called Osteoporosis Clinic, which is having a very long waiting list. Months, four and five and six. **Interviewer:**

Ah, OK. **Interviewer:**

And is there an inpatient and outpatient clinic or is it just outpatients? **Interviewee:**
Just outpatient. **Interviewer:**
How long have you been associated with _____ as part of your employment? When did you start? **Interviewee:**
Since 2003. **Interviewer:**

Could you tell me if you were to say, what percentage of your patient population is diagnosed with dementia?

**Interviewee:**
Uh, yeah, it's good question.
You know Alzheimer is difficult to diagnose, it depends mainly on pathology and clinical assessment. You cannot differentiate exactly, but I can see we can say 50-50, between Alzheimer’s and vascular dementia. Uh. Complications after stroke they start to become demented, and those without any vascular factors without dementia, without diabetes, without hypertension. Uh, I can say 50-50. **Interviewer:**
So, out of the 50% with dementia? Are there patients with other types? Anyone with frontotemporal or with Lewy body? **Interviewee:**
Have a very famous case of frontal temporal. Uh. At young age usually started at young age and with family history of front to temporal, also very unique. **Interviewer:**
OK. Is this? Is this just a single person or just one, one patient? **Interviewee:**
Sorry. It was a phone call, OK. **Interviewer:**
It's OK you can answer it. I can wait. **Interviewee:**
No, It’s okay. Go on please. **Interviewer:**
I was asking about the frontal temporal dementia case. Is it just one person or? **Interviewee:**
Uh, which is very obvious and we can give it the diagnosis of frontotemporal, as I can say one case. **Interviewer:**
And the Lewy body type. Do you come across any patients who have that diagnosis? **Interviewee:**
Uh ah. Yes, I faced them, but of course nothing in my mind right now.
**Interviewer:**

They're not that common like vascular and Alzheimer's, though. Are they?

**Interviewee:**

Yeah, no. **Interviewer:**
Umm. Now when you see patients and their families and they are receiving the diagnosis, how do you define dementia to them? Because the clinical definition is quite, you know, it's full of jargon, but when you're speaking to a lay person, you have to kind of modify the definition. So if I may ask how you go about giving that diagnosis and how you define dementia to patients and families, how would you say, how would you say to them? **Interviewee:**
Uh, actually, we depend on the clinical diagnosis we use a tool in the clinic, the Comprehensive Geriatric Assessment, which is I like more because it's better for those who are not educated and we face a lot of them. Uh, I do not give the diagnosis from the first visit and I wait at least six months to give chance for any reversible causes to be managed and to be sure that it's something persistent, cognitive impairment on the first day visit, I give the diagnosis of cognitive impairment mild cognitive impairment. Then I keep a diagnosis of dementia for the second visit to give myself and the patient the chance to be sure that it's a stationary or progressive course. I depend more than the psychometry on the interview itself. Uh, I check the dialogue with the patient. Logic, abstraction thinking, which is very important for me. About working memory. I give examples ask me how to make for example, a sandwich if a lady, how to cook, give me the steps. Then I reach a conclusion to say that this is dementia case and start to talk about the prognosis. What we can do to slow the progression curve, slow the decline. Uh. Usually I don't give them…I usually try to give hopes and the science is changing…and we have a lot to do and we have medications for the early to moderate [stages]. We have supplements that, from our experience, even if it's not evidence based, it still plays a role. We will be beside you. I'll give them my telephone number to support uh, this is my way. **Interviewer:**
OK. Thank you so much. And how many percentage of your patient population is from the UAE and how many are from outside the UAE? **Interviewee:**
The majority, more than 90% UAE.

**Interviewer:**

Yeah, my next question is about person centred care, person centred dementia care. If you have heard about it, what do you know about it? If you can just take me through it.

**Interviewee:**
Yeah. I'm not familiar with that in practice, except maybe I'm doing it, but I don't know that it is the terminology. **Interviewer:**

OK. Uh, tell me what you know about it. What do you know about person centred care? What have you heard about it? It doesn't matter if you do it or not. I just need to know if you know about it. **Interviewee:**

No, actually nothing in my mind.

**Interviewer:**
Treating the person as a person first and not as the diagnosis that they carry. Honouring personhood. How does that resonate with you? **Interviewee:**
I do it. I'm the best one to do this, but I don't know the name. **Interviewer:**
OK.
Tell me. Give me examples. **Interviewee:**

Yeah, I, we are in geriatrics because we are concerned with the comprehensive care, uh.
And this is what makes us different from, for example, internal medicine doctors. Uh, I approach everything because everything will affect the mind and affect the brain. Uh, I care about eating. I care about sleep. I care about nutrition. Is the problem with the bowel, constipation, recurrent urinary tract infection, which is very common in all elderly, which is a very high risk factor for deterioration of the behavioural issues of dementia. We care about everything, fungal infection, for example, could be a source of annoyance for the patient and he cannot express himself to say that this is and just simple pain can induce a lot of behavioural issues, agitation, aggression, sleeping issues, everything. We are not disease concerned, we are whole person concerned, holistic application. Yes, we are definitely person centred focused if this what you mean. **Interviewer:**

OK. Do the family members get it informed or educated about how to care for their loved ones when the diagnosis first comes?

**Interviewee:**
Yes, yes, I yes, we educate them and we have programs for caregiver trainings throughout out the year. We do it through zoom. Uh, me and my colleagues, my part is dementia. Other colleagues are taking other aspects, all aspects of elderly care we are doing through caregiver continuous training. **Interviewer:**
How often does it happen in a year, this training? **Interviewee:**
One or two sessions. Every month we have something throughout.

**Interviewer:**

Uh. OK. And how do you reach out to the community, via email or on the? **Interviewee:**

Whenever we visit any case, we take the information, the phone numbers and we registered the caregiver for we have like more than six, 700 cases. Uh, so those people are all involved and we ask them if you know someone else, tell them. They are all invited for our zoom education. Uh caregiver support and training. **Interviewer:**
So, this training? Is it based on a standard program or model or framework? Or is it based on your interactions with the patients you just prepare it? I mean you format it accordingly and you deliver it. How does it work? **Interviewee:**

It's a PowerPoint. Uh, that you'd really take all the aspect, the common problems that they may face, the caregiver with the dementia patient, related to making a daily program, a daily routine related to how to deal with the behavioral disturbances, how to manage what? Uh, the role of the medication went to ask for help, like this. **Interviewer:**
Umm. **Interviewee:**

Uh, we try to cover all aspects so everyone can find what he needs in our training. **Interviewer:**
OK. My next question is. As part of your qualification as a uh geriatrician, have you undergone formal training in providing dementia care? A person centred dementia care, the one you just said that you do, or is it just based your experience in dealing with patients with dementia that you developed that strategy? Or was there a formal training in caring for people that way? **Interviewee:**
Actually, my education is geriatric from the beginning. I was a resident in geriatric department for three years. Then I got master’s degree in geriatrics. Then I did a doctorate degree in geriatric, which is the last thing we have in geriatrics. We have 5 cores or geriatrics syndromes. The biggest of them is dementia. So no geriatrician would not have formal education on this disease. In very minute details and throughout our [professional] lives, the continuous education, the conferences and the seminars, uh, and so on. It’s a lot.

**Interviewer:**

Yeah. OK. Now tell me about your care, your team. How many team members do you have?

**Interviewee:**

We are five. All of us are geriatricians.

**Interviewer:**

Yes, I see. And what about the? So mainly geriatricians, as you said, are properly qualified to care for patients with dementia. What about the nurses? Are they trained for dementia? Tell me what type of training they go through. Outside their formal nursing education. **Interviewee:**
Nothing special nurses, routine stuff, nurses and assistant nurses. The other are staff nurses, mainly dedicated for the home care. They have no anything to do with the clinics in the clinics. We get support from the nurses of the clinic itself, just in taking the vital data. Uh, any other? We do it ourselves. Here, we don't receive support from nurses in our work actually, except in the home care. **Interviewer:**
Umm. Now, from my participation on the [Alzheimer’s], I've been watching the trend of how family members interact, and there seems to be a trend about umm, how people with dementia are cared for in their home environment. We know the Emirati culture encourages family members to take care of their loved ones. There isn't really the concept of care homes or permanent nursing homes does not really exist in this culture, so it's usually families who care for their loved ones. But from my understanding, the idea of home care agencies providing nurses that go into the homes of these families to give direct care, that seems to be very common in this part of the world. Can you tell me something about that? What do you see in your practice? Do you come across family members such as sons, daughters, wives or husbands giving care? Or is it the hired help? Who does the daily care?
What is it like, the dynamics in the home environment? **Interviewee:**
Actually, yeah. And even those who hire nurses, which is the majority, uh, still the daughters are the main ones, not the sons, and in few cases the sons, Uh [who are] supervising these nurses and every minute details. The nurse are just doing with their hand, but with the supervision of the daughters mainly. And in some cases, also the daughters and the sons are taking the first hand, having no nurses because the nurses are mainly [covered] through insurance, but the insurance does not always cover the nurses unless if the patient is on tube for feeding, for respiration which is not all the cases. **Interviewer:**
Umm. **Interviewee:**
Most of the cases of dementia are not without these issues. They need like caregiver and nursing but insurance does not support for covering caregivers. So, what [often happens is that] they bring domestic helpers on their own visa from India, from the Philippines or they make shifts between the daughters and this kind of arrangement. **Interviewer:**
Umm. **Interviewee:**
In general, I can say, uh, the families here are doing great job. They are very dedicated and faithful to their old ones. That's what I can say. Yeah, any uh, I can give a score of nine out of 10 for the role of family here in caring for elderly. **Interviewer:**
I see. And are they [the families] the people who attend your Zoom trainings? Or is it the hired help?
**Interviewee:**
The hired help. **Interviewer:**
OK. What about family members? Is there a reason why they don't attend? **Interviewee**
Maybe because they are busy. Uh, actually, actually, I'm not sure, but I can roughly say it's mostly the nurses and the caregivers because I can check them. **Interviewer**
Uh-huh. OK. **Interviewee**
I can guess that most are nurses or caregivers who attend. **Interviewer**
OK, I see when we say the hired one, it's from these agencies that we're talking about, not the domestic help that they bring from places like India or Philippines?

**Interviewee**
No, no, both, both. **Interviewer**
Both. OK, which is more common, doctor? **Interviewee**
Uh, the nurses highed the through the companies. Uh, are the majority. **Interviewer**
I see. And that's because there is insurance coverage or people pay out of their pockets when needed. **Interviewee**
Some of them, they pay some of them they hire and some of them, if the case is not indicated, they keep, they keep pushing [for coverage].  **Interviewer**
OK. Now, how do you typically address a patient with behavioural or emotional challenges?
Not just you, but your team, the nursing team, the physiotherapy team. When you go for home care visits how our behavioral challenges addressed things like agitation, aggression in some cases, generally, what is your approach in addressing those kind of situations? **Interviewee**
Actually, we teach the family and the nurses first about the non-behavioural tips. Usually when there is a new behaviour emerging. Yeah. We tell them try first exclude any medical and physical reasons. Uh, like infections like pain. Uh, I usually make a full operatory when it's new behaviour emerging.
If I find something, I start treatment mainly like infection. Just infection. Expect pain, sometimes I ask them to give panadol as a trial every six hours for five days to see the response because it could be a pain and the patient is not able to see. We check the nutrition, hunger, thirst, these issues. If we did not find anything, we start to talk about behavioural tips distracting the patient. Try to make it daily routine to involve him not to leave him without doing anything, because vacancy in itself could be a trigger of aggression and agitation. Try to use any artwork involving in preparing anything in home, in cooking something safe that could not harm him, but don't leave him like this and then suddenly notice and say, what is making him agitated and aggressive. Sometimes the patient misinterprets his vision, as if something is happening to him or someone is harming him like this, a lot of them complain like this. I want to go my home. This is not my home. Try to take him in a journey with your car. Uh for 15 minutes and come back home, try to make any physical exercise like walking to take his energy. Uh, try relaxing rooms. This is uh, after failing in everything I consider medications, yeah.

**Interviewer**
OK. And these are antipsychotics, typically. **Interviewee**
Uhuh. Yeah.  **Interviewer**
OK. And does that help in most cases? **Interviewee**
90% at least. **Interviewer**
Uh-huh. OK. What would you say is the proportion of male to female ratio? Who do you say is most commonly diagnosed with dementia? Is it more common in males or females? In the Emirati population mainly? **Interviewee**
Actually both I'm not having uh statistics in my mind. Uh, but both? Yeah, you, you know, maybe males are more liable to vascular dementia, to strokes. That's why we can see vascular dementia much more in male and Alzheimer’s much more in female. I can say it like this. **Interviewer**
Why do you think that is? Alzheimer's, why do you see it more commonly in females versus males?
Do you think there's a reason? **Interviewee**
Umm, because here people now have longer lifespan, and you know age is the most important risk factor in Alzheimer’s.

**Interviewer**
Do you see cases with younger onset dementia becoming more common, how common is it? Younger onset or early onset dementia in this part of the world? **Interviewee**
Yeah, I see it, not too many cases, but yes, I saw cases in the 50s. Uh, and usually it's very aggressive, very aggressive. Those who started early before 65, I have few such cases. **Interviewer**
OK. Let's just talk about those for just a couple of minutes. Obviously, with patients diagnosed at the late stage of dementia umm, then of course they may no longer be able to make decisions for themselves because the disease has progressed. But those with younger onset dementia, or even those in the early stages of dementia, even if it's diagnosed late in the in their age, are they [socially] able to make decisions for themselves? Or is it common for the family members to make decisions for them? What's the trend like? Who makes the decisions in terms of receiving care and management of the disease? **Interviewee**
The family.

**Interviewer**
The family. What do they do? Do the patients have a say in any kind of decision making. For example, if they were living alone once the diagnosis is made, is it common for the family members take them into their homes? Are they given a choice or it's the family members who make those kinds of decisions. From your experience, what have you noticed? **Interviewee**
Families are the ones making the decisions. **Interviewer**
And are patients generally okay when decisions are made for them on their behalf? What is the culture like?

**Interviewee**
Usually in the in the very early, when the patient still can make decision, uh. Really, we don't see these kinds of patients a lot. Usually the ycome a little bit late than this stage when they are no longer able to make a decision. **Interviewer**
I see. **Interviewee**
Because mostly they consider it as part of aging. They don't come to us. Usually they come to us when there is behavioural struggle, or extreme cognitive deterioration, or they start to get lost with the car or they go out and they are not able to come back home. **Interviewer**
Umm. **Interviewee**
They get lost at this stage. Usually we are not talking about early Alzheimer’s. **Interviewer**
Umm. I see. My next question, can you think back, you know, from the recent past or maybe a long time ago, a case that stayed with you a case where you could not manage a challenging behaviour coming from a patient. How was that like, where you tried all means but you couldn't manage it. It could be either you or the nursing staff. Any kind of case that comes to your mind and how that situation was handled. Umm. Because there are some cases where it, you know it's very difficult to figure out what the patient’s needs are. You've tried all means, but you're still not meeting their needs. **Interviewee**
If there is a case like this, we go for antipsychotics. As I told you. At this point of the disease, the family maybe is the number one target for me because the patient is no longer aware about anything, is becoming just a source of annoyance for the family. So here I consider the family even if I give him a dose of antipsychotics, he will not suffer anything but at least to relieve the family. Uh. **Interviewer**
Umm.  **Interviewee**
Yes, we do not reach 100% management of behavioral issues with everyone, but at least we reach a stage of making life possible. Making life possible, yeah. **Interviewer**
Any other things that you'd like to add before we finish the interview? Anything you would like to add? **Interviewee**

Oh yeah, I would like people here to understand the importance of having, if in the future any long term institution for dementia patients, for better quality of their life and safety. Uh, not to be considered like elderly home and not to be considered like you're throwing your loved one in a place and neglecting them and to feel shame and to feel harm. No, we can help them more. Uh to design specific institutions? Spacious, that allows them to walk and walk and walk and to get all of their negative emotions out and to. Exercise them so when they come back to your home, they're already relaxed. Have no any energy for agitation and aggression. Uh group therapy, and this kind of stuff like we have some examples in Western countries, like a dementia village. **Interviewer**
Mm-hmm. **Interviewee**
Uh, my main fear is that people may look at it…consider something like this as negative, again, like it will be like throwing your loved ones out.

**Interviewer**
Umm. **Interviewee**

But, no, we are not throwing. We can make it like daycare or something like this. **Interviewer**
Yeah. **Interviewee**
Uh, yeah, we need to change the image a little bit. Not everything outside the home is negative. **Interviewer**
Last question. What are your thoughts about doing research?

**Interviewee**
Yeah, yeah, yeah, sure. Research is good. But I am not a researcher because no time…and we have forgotten… academic life was a long time ago. **Interviewer**
OK. Thank you, Doctor. That's all I have.
**Interviewee**
No problem. Nice to meet you.  **Interviewer**
Thank you so much. I appreciate it. **Interviewee**
Thank you.

**Interview with Geriatrician #3**

**02 June 2023**

**Interviewer:**

My first question is, How would you describe your services? If someone who has no knowledge of your services comes to you, how would you describe it?

**Interviewee:**

Ehmm, It’s a community hub, for seniors. Ehm It’s a very unique place where ehm seniors feel at ease and this is applicable to both the inpatients, those who are staying with us and the outpatients, the community people, ehm it’s a place that makes them comfortable and happy, ehm it’s a place that gives Dubai an excellent reputation when it comes to caring for seniors. It’s a place that would remove the stigma that comes along with nursing homes and even for those people who have that idea or that link between the nursing homes or elderly centers with sadness or depression or I mean sad places, once they come and they see the situation they change their minds. So, we are working [to] influencing the community and trying to change that negative image about elderly centers.

**Interviewer:**

Would you say overall in the population of Dubai there is a stigma attached to not just getting old and what comes with old age but specifically also with dementia?

**Interviewee:**

Ehm Sort of, yes, but I think now it’s improving with time, and I can tell you that, we started announcing Alzheimer’s Support Group in 2013 and we wanted to call patients and their care givers and those who are interested about dementia to come and share their experiences, talk about the illness. We’ve noticed that ehm very few Emiratis were coming, because again we felt the stigma at that time was so obvious, they don’t want to come, they don’t want to say that my father has dementia or my mother has dementia. They used to feel it’s not it’s not ehm culturally it’s not, it wouldn’t be accepted ehm.. We had a lot of expatriates coming at that time but now ehm like that was 10 years ago, Now I feel ehm.. I mean, okay, still people are not looking at dementia in a negative way but they are afraid to get it. Once if they have any family member who has it, they will go and speak about it ehm…freely without…or they will go and ask for an opinion without being or feeling shamed, that they don’t want people to know that they have an affected member so it’s changing, yeah exactly. Because there is more awareness, people are aging ehm…they can see more cases of dementia being diagnosed, maybe I mean patients are being there before but there was lack of diagnosis and now people are starting to talk about it and they are able to pick up the symptoms that they can see and their parents for example, they can come and ask.

**Interviewer:**

Where do they typically go when they need such information? Do they go to their primary doctor? How does that process go?

**Interviewee:**

Okay, it’s not very clear yet because people are not yet ehm…sure where to go ehm…I mean very few f them would speak about the cognitive changes with their primary caregiver or with their primary sorry- physician, in their primary health care centres ehm…some would go to memory clinics which are there actually there are I think two memory clinics under Dubai Health Authority…ehm or “DAHC” Dubai Academic Health Corporation so they would go to the memory clinics ehm.. some would go to ehm… places like psychiatry units in government hospitals, some would come to us directly those who know the centre and who know that we are here I mean we can handle these issues, but it’s not clear and a lot of them would go to the private clinics and hospitals, and again in the private sector in Dubai, we don’t have a lot of geriatricians…but again people are not yet aware about the presence of the geriatricians so at the level of the private usually they are seen by neurologists.

**Interivewer:**

OK.

**Interviewee:**

so, the approach is not yet clear I mean people in the community are still not sure where to go and whom to contact in case they are noticing problem with cognitive function.

**Interviewer:**

Would you say along with the stigma, once ehm you know family members notice changes and then they are aware that it has to do with Alzheimer’s or dementia in general, then that’s when the stigma comes because there is that awareness, would you say that’s how it is in general? Obviously, we can’t really generalize this but on average people lack awareness of what dementia is or they know it and then they have stigma towards it. What has been your experience?

**Interviewee:**

It’s both , it’s both. They lack awareness and some of them they might know it but again they are reluctant to go and I mean maybe they know that the parent or their parents are having dementia but they don’t want to hear that diagnosis from a physician so it could be both.

**Interviewer:**

How long have you been working here? In this capacity? Tell me a little bit about your services.

**Interviewee:**

Since 2012. We are expanding. We are adding new beds so that we are planning to increase our capacity so this is what happened, yeah in 2001 the focus was mainly on in-patients there was very little number of outpatients. We even have a graph calculating number of outpatients from the time I came in 2012 we opened more floors for the outpatients to come. There is a lot of potential to serve the community by opening clinics, by introducing new services to the centre and this is what was done, by opening the expansion on the other side where we have more rooms for physiotherapy, rehab, hydrotherapy, for occupational therapy, and the clinics. So, I remember the outpatients were only like 58 when I came in 2012, and now our outpatients are more than 1000 maybe yeah. They come according to their appointment, they might like have twice per week physiotherapy or three times per week physiotherapy, they come to the clinic, or some even come for social gathering. So, we have increased the potential of the center to serve the community by increasing the number of staff increasing the services or introducing new services.

**Interviewer:**

So, it’s an appointment-based system. You don’t have…on a given day, you wouldn’t have to a thousand outpatients. There is a system put in place.

**Interviewee:**

Yeah, they come according to their appointments or sometimes we call them and maybe the maximum number we can have is a hundred, if we have like any special occasion. In Ramadan if we’re having special Iftar gathering, we will just simply call them.

**Interviewer:**

Now moving on to, just to move briefly to your staff. How are they recruited? what is the recruitment process?

**Interviewee:**

Ehm, okay the recruitment process is ehm…by the second half of the year, we would specify our budget and this includes the manpower budget, so if we say we are planning to have a new service like our occupational therapy, if it was not there [before] we would say we want an occupational therapist and then once we have a budget code allocated for us, because we will have allocated budget for equipment that we need for next year, ehm operational work, we need projects, do we need to expand something, do we need a specific project, then the manpower…so once we have the budget code then we can recruit staff. We then get a number of CVs through the HR, we will do the interview in the centre and we will be the ones who will do the interview based on the specialty, for example, if it was a nurse we will involve the nurse in charge, and if it is a physician, the physician would be involved, and admin, and it works like this once the staff are recruited they will have an orientation to the centre. we will have very clear orientation program for them, and we need to complete their files actually with a lot of documents that need to be submitted. We need to make sure their license is active and valid and they take at least the essential mandatory training that should be done.

**Interviewer:**

I see. So, orientation plus training, is the training standard across departments or…?

**Interviewee:**

Ehm…there is a standard training and there is a specific training, based on the specialty.

**Interviewer:**

OK. Now ehm, coming to the patients what’s the male to female ratio?

**Interviewee:**

Inpatients? It is always more males than females.

**Interviewer:**

Why do you think that is?

**Interviewee:**

Because ehm you know our admission criteria states that the patient should be Emirati, above the age of 60, with no social support, no children and usually women are protected in the community. Even if she doesn’t have children, still other family members would take care of her. They wouldn’t like us to keep her here. So, it’s always more males than females.

**Interviewer:**

So, it has nothing to do with stigma. It’s just…

**Interviewee:**

Yeah, yeah. Females are more protected in the community. It does not really accept the idea of admitting the females to the centre. I remember we once admitted one lady who fitted really our admission criteria: she didn’t have children, she was in need of the services, so we admitted her, and I think through Community Development Authority, CDA, after the admission, she improved a lot, and then her second degree family, they didn’t feel happy having her here so they arranged to take her. Actually, which we do we do accept because at the end we always believe family, ehm ehm the elderly should be with their family. It makes a lot of difference, but we do, I mean we do admit those who cannot be with their family in special cases or who cannot or who do not have a family actually.

**Interviewer:**

OK. About your outpatients, do you have a specific number or it varies?

**Interviewee:**

Yeah, the outpatients, it varies according to the number of ehm…transactions, we say I mean once we get our statistics we get it based on the number of transactions how many patients came for physiotherapy, it appears on the registration, so, this can be from 1000 to 2000 every month, yeah, but I mean imagine each physiotherapist would see like ehm 10 patients per day.

**Interviewer:**

OK. Would you say that there is that individuality, honoring of personhood, in terms of how you care for the patients?

**Interviewee:**

So, coming to patient centered care that you’ve asked me about before, I think we are applying it somehow in the centre, by allowing the patient to take the lead in the decisions that are related to their preferences, ehm ehm, if they like to decorate their room in a certain way, we don’t mind and this is even part of the JCI John Commission International accreditation for the standard of long term care, this was part of it and we say yes, we allow them, you know, we just allow them to bring their own material their own stuff and to select the timing for the service that they want, so, for example, if they don’t want to wake up early, or they don’t want to do physiotherapy today, then it’s fine we are. Ehm, actually since the last 7 or 8 months, we started even introducing physiotherapy over the weekend for them and we ask the staff who are willing to cover the weekend with extra payment in order to make sure that that our inpatients are having physiotherapy on a daily basis and including the weekend. This has made a lot of difference actually.

**Interviewer:**

OK. Is everyone who comes to use your service is diagnosed with dementia?

**Interviewee:**

No.

**Interviewer:**

How many would you say are not diagnosed? Because you have told me you don’t have a Neuropsychologist on site? Who makes the diagnosis?

**Interviewee:**

We don’t have a neuropsychologist, but we can diagnose dementia without a neuropsychologist, yes. At the end, you cannot…until now, we cannot say for sure, we do not have a single diagnostic test for dementia, but we say this Is most likely dementia.

-------------Interruption -----------

**Interviewee:**

They just come to say Hi.

**Interviewer:**

The outpatients you mean?

**Interviewee:**

Yeah, but I mean see they don’t come for their appointment they come because they are attached to the place, yeah. They like it, they feel happy, I mean, they come to talk a lot of them they just they simply want to talk they want somebody to listen to them.

**Interviewee:**

How involved are family members for both inpatients and outpatients?

**Interviewer:**

Ehm for the inpatients, most of them don’t have children, they might have second degree relatives. We try our best through our social workers to call them to maintain a good relation with the patients, and this is happening for most of the cases. Sometimes, we feel we face challenges but, ehm I can say we have excellent staff who are able to handle or bring families to make sure that they visit the patient and ehm, we have our log book, we have the name of the visitors coming to which room, and what’s their relation, and we keep an eye on those visitors who are coming and making sure that the family are coming during Eid, for example. We encourage them to come. Ehm, we make Eid feast ehm breakfast or lunch, and the family, they do come, once they know that the patient is settled…if they considered the patient as a burden and if it was difficult for them to handle him once he is here and that burden was removed from the family, they’re happy to come and just have a look into the patient and make sure that he or she is fine.

**Interviewer:**

So the relationship continues.

**Interviewee:**

Yes exactly**.**

**Interviewer:**

And with the outpatients, are they brought here by their family members or transportation. How does that work?

**Interviewee:**

No we don’t have transportation. They come either by their own cars or by the family or by their drivers or their children.

**Interviewer:**

What proportion of your patients would you say are diagnosed with dementia, clinically diagnosed with dementia?

**Interviewee:**

Ehm, inpatients? I would say 40 percent, maybe ehm, and outpatients it’s difficult because we see different cases. It varies. We see ages from 60 because our clinic start at 60, from 60. We don’t have a health registry, so I cannot, we cannot find out the exact percentage of dementia. I don’t know.

**Interviewer:**

And what proportion of your patients is not diagnosed with any kind of condition, or are there always health issues attached to admissions?

**Interviewee:**

Yeah, there are always health issues. I mean, we don’t have a patient who does not have anything at all, ehm. With old age, they would have hypertension, diabetes ehm, other ehm, chronic diseases, we don’t have a patient who has nothing.

----Interview Interrupted due to work responsibilities----

**Interview with Geriatrician #4**

**20 September 2023**

**Interviewer:**

My first question is about your position. What do you do?

**Interviewee:**

I'm a geriatrician. I've been working here for the last ten years, when we started.

**Interviewer:**

Okay. Were you transitioned here from another government hospital?

**Interviewee:**

No, this was my first job here in the country.

**Interviewer:**

Okay, okay. These are just demographic questions. What's your training, qualification?

**Interviewee:**

I had my MBBCh first, and then I got a residency program. ended with a Master's degree in geriatric medicine and gerontology. Later on I got MRCB UK, which is a certificate related to internal medicine, and after that I got SCE which is a certificate, SCAE, specialty certificate examination in geriatric medicine from the UK as well, and after that I got the clinical leadership diploma from the RCS Ireland, and later and latest, uh, was a European diploma in palliative medicine.

**Interviewer:**

So, before I go to your patients do you know anyone outside your work environment who is living with dementia? It could be family, friends...

**Interviewee:**

Family, even some of... my friend's relatives as well. I'm seeing them as a physician. Definitely, and even, relatives, I'm the one who is taking care of them, and uh, in respect of medication, giving advice, not-not giving care for them.

**Interviewer:**

Okay. Currently, what proportion of people under your care are diagnosed with dementia?

**Interviewer:**

It's hard to say, but I can give you like a range, not less than 50% of the patients that I face in the clinic, and around as well 50% of the patients who are under my care, as in, in-patient, are demented.

**Interviewer:**

Around 50% in-patients and also the outpatients?

**Interviewee:**

Yes, so you can generalise the data.

**Interviewer:**

Okay. And are they different types of dementia or…?

**Interviewee:**

Yes, according to different stages of dementia, mild, moderate or even later on or severe stages, when we get them only by later, no more.

**Interviewer:**

Okay, so, in terms of stages, or as well as types of dementia, they are quite varied, you'd say?

**Interviewee:**

Yes, yes.

**Interviewer:**

Okay. Which one is the most common?

**Interviewee:**

According to type, we definitely know that majority of them are Alzheimer's dementia, followed by vascular dementia, this is the majority of the case that I can see. and according to severity uh, and orientation of the culture that we live in, we face dementia mostly in the moderate stage or the late stage. Few cases only who are coming in the mild form of dementia.

**Interviewer:**

And are these early-onset, or..?

**Interviewee:**

Majority are late-onset, but we still do have early-onset dementias as well.

**Interviewer:**

Okay. Why did you mention culture, just now?

**Interviewee:**

Because, understanding the patients themselves, when they will come to complain, is different from culture to culture. I have a practice as well in the UK before. That's why I'm telling you, that maybe the patient, to be honest with you, at the memory clinic, they will come early-early, when they will have a just few symptoms. So you can catch patients in the mild stage, or even when they are in the MCA.

**Interviewer:**

But here, it's different?

**Interviewee:**

To be honest with you, no, they mostly came late.

**Interviewer:**

They come late, because it has something to do with culture?

**Interviewee:**

Yes.

**Interviewer:**

Let's dive into that. It wasn't part of my interview, but because culture is a big component of my project, I am really interested in knowing your perspective as well. What part of the Arab culture, or the region's culture, do you think influences that decision…to come in late..?

**Interviewee:**

Okay, okay. Let's start from the beginning that the age of retirement in our countries is much earlier than the Western countries, so a person can find himself, at the age of sixty, and in some countries as well, earlier than that you'll find themselves is not an active part of the society, to be honest with you. Plus, the presence of support here is much much much more than in the other countries, so a patient or a person can depend on the others arranging his appointments, arranging his health needs, and this makes uh, what, the exploration of his disease, coming late. On the other hand, if we look at the culture itself, they do understand and respect that having a problem with the memory is a normal part of aging, so they did not seek medical attention early as well. So we can see the presence of support, and the understanding of disease itself, may be late...

**Interviewer:**

And uh, having social support, be it from family or... in what way does that contribute to the late diagnosis? Is it because those people depend on others, so that cognitively they are not challenged, or is it because other people are taking care of them that they don't speak on their behalf?

**Interviewee:**

Ah, okay. Let's have two scenarios. If an elderly person lives alone, and he starts to have a problem with his memory, this will be easy to catch so you will find difficulty to go to places, he will find difficulty to handle his money, and he will try to find the…the problem cause he is the one who is taking care of himself. That's why he will take attention. While if the patient is having or surrounded by all these kinds of support as I mentioned…okay…you don't need to handle the finance, you don't need to remember the places, a driver will take you to wherever you need.. this will make it to later on to discover that you really have a problem or you may ask for help.

**Interviewer:**

I see. I mean, this is a completely new perspective that I didn't receive previously, so thank you for that. Now, this is just because this is part of my interview schedule, how would you diagnose dementia as a condition?

**Interviewee:**

Yes, dementia is chronic, quite problem as you mentioned it is a neurogenerative disorder. We are expecting it to become worse and worse with time, that the patient is having decline in more than one of the brain domains or the cognitive domains. You need me to go further?

**Interviewer:**

Yeah, talk about age and how it contributes. Age as a determining factor, is it a risk factor?

**Interviewee:**

Yeah. Yes, yes, definitely, it's the first one, the first responsible, actually, is the age. As we can see that the uh...percentage of dementia is increasing with the age, and as far as I understand, after eighty it becomes almost 50%.

**Interviewer:**

I see. The next question was from a training, but you have to explain that…did you receive specialised dementia training...caring for dementia patients, or was it part of your general training?

**Interviewee:**

Yeah, it was part of my residency programme.

**Interviewer:**

Okay, and how long-how intensive was that, would you say. Were you rotating, or..?

**Interviewee:**

Uh, yes. I had a rotation dealing with patient and, admitted patients or in-patients, I saw them at the clinic as well, and part of my practice was to see patients at home, especially those with cognitive impairment.

**Interviewer:**

Describe a typical day during your practice activities at the centrr, what does a typical day look like? what time do you come in, how many patients on average do you see, what kinds of challenges do you experience, what are your high moments, happy moments, what are the challenges?

**Interviewee:**

In regard of dementia or in regard of my work? I have both of them.

**Interviewer:**

First start with the general, then go specific.

**Interviewee:**

Okay, not all the days exactly go the same, cause according to my position and according to the schedule that we have in our clinic it's different from day to day, but we can say on average that I'm reaching my work early in the morning, around 7 o clock. I'm attending the handover of the nurses, I'd like to attend what the nurses communicate to each other, it's the time to catch the problems early, and after that I'm trying to start my admin job for the first one hour, because part of my job is as a head of the medical section, then are start by my daily rounds, in-patients seeing what's going on. To be honest with you, this center work as a long-term care for elderly, we don't need to go and dig deeply in each patient more that once a month, that's the requirement, but that's not what we are doing here, we are doing the rounds daily to catch any new problem happening with the patient. We have to check their baseline, if there's any change from the baseline they'll need to do extensive examination. At least, you need to see that this patient is going with his baseline nothing here. So there's a dementia-sorry-for this patient dementia, delirium is uh... common thing to happen, so we need to catch early, plus the medical issue of the patient we handle it like in the first two hours of my day and then I'm going to my clinic. Our clinic is busy as well. We can see around ten to sixteen patient a day; as I mentioned, 50% of them are demented patients. So, loads to do; In-patient, out-patient, finishing things related to my admin job and by going home as well every few weeks I find myself on call, responding to the calls if there’s anything with the patients. Yeah, it's hectic. Sometimes we have afternoon clinic as well, the same day.

**Interviewer:**

Why 'sometimes'?

**Interviewee:**

Because we don't have an everyday afternoon clinic, we only have twice a week.

**Interviewer:**

And are you in everyday, or on rotation?

**Interviewee:**

No, I'm in every day.

**Interviewer:**

Everyday?

**Interviewee:**

Yeah. Despite being on call even

**Interviewer:**

and the number of patients?

**Interviewee:**

Yes, and I forget to mention that as you have seen right now part of our job is receive residents of family and internal medicine; we supervise their acts on the patient with us and we are teaching them the most important thing, or what we call it, the geriatrics giants, one of them is age.

**Interviewer:**

You described a difficult day, now tell me a good day; when you go home to your family friends, what kind of day do you say is a good day?

**Interviewee:**

Ahh.. a good day is a day that you feel that you really made a change for the patients, when you are getting positive feedback from a patient. For example, a patient who was supposed to, I was describing once case right now patient was referred to us as a case of vascular dementia but he was young and by making a deep or taking a deep history, we say that this could be something like pseudodementia. Started antidepressants and after one year you can see that the patient is carrying themselves is a totally different person, and alhamdulillah we didn’t start wrongly in the medication for dementia and we put her in the right way. So when you are getting feedback, positive feedback from a patient, this is the moment you have happiness.

**Interviewer:**

Regardless of how busy you are?

**Interviewee:**

Yeah

**Interviewer:**

What about a challenging one?

**Interviewee:**

It is challenging…I can feel that everyday is a very busy day (laughs) so we are taking the whole weekend to be able to breathe.

**Interviewer:**

But what kind of…give me an example of a difficulty that made you really stressed.

**Interviewee:**

To be honest with you, the most stressful day is when you have a busy schedule in the morning and you hardly reach home and you get a call that one of your patients is really sick and you have to come back and this is happening not really frequently, but it’s really happened like twice a month so you have to come back see the patient and to go home late to stay as an uncle and to come tomorrow during the same hurts the most, mostly its a physical stress more than .. yeah.

**Interviewer:**

Would you say that the people you care for that have dementia are involved in making decisions for themselves when dealing with the care staff, their family members?

**Interviewee:**

It depends on the case, if they have a capacity it definitely helps with that but in the late stage when they lose their capacity definitely they are not able to help or take decision.

**Interviewer:**

So, who makes decisions on their behalf?

**Interviewee:**

Their family and their care providers are called into what's needed.

**Interviewer:**

So immediate care providers are nurses in this case…?

**Interviewee:**

No, it's the physicians.

**Interviewer:**

Physicians.

**Interviewee:**

Yeah

**Interviewer:**

Mm, in terms of diet preferences, clothing preferences and times of waking up, do they get involved in making decisions or is it routine?

**Interviewee:**

As I said, if the patient is having capacity, he has all the right to choose whatever he wants to do, I need to eat this form I need to sleep at this time we are not interfering as long as he is able to make decision. If patients lose the capacity we are going with the daily routine. We have a day routine in the center which is really helpful, I don’t know if somebody has described for you earlier of not, so a majority of the patients are getting up at the same time, they are receiving their food, after that their daily plans, after that the rounds, after that they are going for the social activities and going home for lunch, after that for lunch and in the afternoon they are trying even to entertain them a little bit, receiving their dinner, and for some of them, we are arranging outing once a week or more if they request to that, and by bedtime or by sleeping time majority of the patients are sleeping without any need for aid. I have zero of my in-patients receiving sleeping tablets, it is one of the good achievements. To be honest with you, for the out-patients it is not exactly the same cause we are not sure that the family is following what we are saying it. We always advise them about the orientation, what to do how to keep them active and avoid the day nap if possible so they will not face any problems with the sleeping habits, but majority of the patients come in asking for sleeping pills or having problems with the sleep. But I guess it is about if you are able to apply a day routine and a nice way you will not need to give medication.

**Interviewer:**

Nice. And if um, for the ones who don't have capacity- cognitive capacity is there any form of resistance from their side since they can, let's say for example even if the person lacks cognitive capacity if you give them foods that they don’t like, there are ways that they communicate that with you. How is that received by your clinic?

**Interviewee:**

If they refuse the food? Uh…

**Interviewer:**

Let's say if they don't have the capacity to communicate verbally or...

**Interviewee:**

Yes so we won't feed the patient if you are speaking about if you are speaking about the food itself we will not feed them actually but yes actually we have a, it doesn't have to be...if its medications or something our staff is really good at that they are trained here for that, especially for those with cognitive impairment. We know that they have short attention span, we are trying to divert the attention, and later on to something else and…yeah that works.

**Interviewer:**

And do your staff receive formal training specifically for dementia? Let's say for example, Ms. ____ joined 2 months back in July I remember, on the handover that day, I happened to be here. So, as a new head of nurses, does she or any other nursing staff, do they undergo formal training regarding caring for dementia patients?

**Interviewee:**

Ah... we are doing like...

**Interviewer:**

It's not a trick question, by the way.

**Interviewee:**

No, no, I'm just trying to tell you what is going on, because I don't need to fake any of the answers. Uh, most probably we are advising the nurses during our rounds about any questions they need, we are doing uh, Alzheimer's support group and they're attending and even sharing with their knowledge. With that we do give them lecture in a regular way so it’s not exactly assigning what we do, but it is coming with experience and coming with the knowledge that we share but as far as we understand it is not part of their mandatory...

**Interviewer:**

Training or orientation? And you mentioned Alzheimer's support group, who's that for and how often, and who runs it?

**Interviewee:**

No, I can show you this, but prepare yourself it is going to be a hectic one because daily we are receiving around 200 messages on the Alzheimer's support group. Shared by care providers and the patients, relative themselves. We are inviting them almost every one month for coffee so whoever uh trying to have a real connection between the caregiver, they will support each other and they are even sharing some of the items as donation, some advice, what challenges they face when they are caring for an Alzheimer's patient and how they were able to overcome all these challenges we are learning from; it’s not only that we are teaching them, and tomorrow as well we have an event if you'd like to come.

**Interviewer:**

Where does it take place?

**Interviewee:**

It takes place by 9 o' clock tomorrow.

**Interviewer:**

How do you accommodate 200 people? Or not all of them show up-?

**Interviewee:**

Uh, I'm not sure all of them will attend but I'm saying that the group is really big but not all of them attend all the things.

**Interviewer:**

Okay. So since you mentioned it is specifically Alzheimer's the support group caters to, what happens, Let's say for example, if you have a patient with frontal temporal dementia-?

**Interviewee:**

They will be added to that one of course majority of them, as I mentioned, will come to care little bit late when at the end differentiation is not that much.

**Interviewer:**

Uh. So you just-?

**Interviewee:**

Yeah, yeah, exactly, the difference will just be in the controlling the risk factor and putting them on the treatment but the complication they are coming for, the complications and issues and the treatment in these issues is not different for different kinds of dementia.

**Interviewer:**

Okay. Generally, without focusing on one specific patient, what does a treatment plan include for a person newly diagnosed with dementia from a biomedical point of view?

**Interviewee:**

Yeah, uh, according to stage definitely according to agreed practice uh, and according to the stage to the type of dementia itself we will go with a non-pharmacological treatment. It will be exactly the same, and to the pharmacological*…phone call interruption…*

**Interviewee:**

Sorry.

**Interviewer:**

I'm sorry for keeping you.

**Interviewee:**

It's okay. I was planning to go home by 12 o' clock but I know you are coming from outside.

**Interviewer:**

Thank you very much. I really appreciate it. So you mentioned..

**Interviewee:**

Yeah, so we will see this patient is having which type of dementia, and according to the approved treatment we will start with him this is the pharmacological one and we will deal with the non-pharmalogical one; we will explain to the family or the patient with us exactly, we will explain how to deal with him, what's the type of complication expected to be happen with him, even with time so we don't look for the point of the diagnosis but we have to explain the illness trajectory and how it will end with time, and what's our goal of treatment and what we are trying to delay or to avoid.

**Interviewer:**

So the non-pharmacological treatment plan includes just providing advice, what else does it include?

**Interviewee:**

Yes, yes, providing advice, what kind of exercise they do, how their food should be like, how they'd like to deal with him on a daily base, how to orient the patient, and how to reorient the patient, what expected complication they will face with the patient, and even they start to speak about the treatment and the goal of treatment to the deterioration, and then we will see them in a regular pace: asking how they feel communication is working or not, shall we continue it, so it's almost a holistic treatment, we will offer them to be enrolled in the Alzheimer's support group we will explain to them what they can face like caregiver stress and how they will overcome all these things.

**Interviewer:**

And pharmacologically typically what medications-?

**Interviewee:**

Typically, we have 2 kinds of approved medication, will now let’s speak about that we have ______ we do accept to start the mild to moderate stage of Alzheimer dementia on the first group and on the moderate to severe dementia we can go with the second group. Sometimes, for some cases, we are trying a combination and seeing if the patient is really getting a benefit from the combination, we can continue it. But by seeing the patient, if there is no real improvement in that we can start to go with only one medication, and definitely when the patient’s function is deteriorated and he is getting into the rough stages, we are stopping the medication when there is no use of.. yeah, uh. Are you aware that there are new classes of medication coming but till now it’s not really approved that it's safe to be given to the patient specially in the late stages?

**Interviewer:**

This is applied now across the board to any patient that is newly diagnosed with dementia or does the person have any, or are they involved in the decision making? Let's say for example-

**Interviewee:**

I didn't get you, whom? The patient himself?

**Interviewer:**

The patient, are they involved in making decision with regards to the medication they receive if it’s in the mild stages lets say and they prefer..

**Interviewee:**

Yeah yeah, we have to explain the medication, what's the idea..

**Interviewer:**

What if they don't want medication?

**Interviewee:**

It's their right.

**Interviewer:**

Ah, no obligations.

**Interviewee:**

Patients can refuse the medication. [that’s] one of the patients’ rights, they can refuse the medication if they have the capacity.

**Interviewer:**

And if no capacity, does the family make the decisions or just the doctor?

**Interviewee:**

Uh the situation, yes, the family can help, so you have to open it up and discuss with them, and then you are trying to push them actually toward the right thing to be done.

**Interviewer:**

In a convincing way?

**Interviewee:**

Yeah.

**Interviewer:**

Have you ever heard about person centred dementia care?

**Interviewee:**

Yeah.

**Interviewer:**

What is your awareness?

**Interviewee:**

Patient centered care?

**Interviewer:**

Person centered care

**Interviewee:**

Sorry.

**Interviewer:**

Person centered care.

**Interviewee:**

Yes, that we are trying to do all our intervention and care just for what is good for the patient.

**Interviewer:**

Okay, uhm, what do you do? You mentioned earlier delirium yeah, what do you do, first of all is delirium by default something that every dementia patient experiences or is it selective?

**Interviewee:**

No, but a patient with dementia is at higher risk of developing delirium, so we have to be cautious that they can enter into delirium and that is what I mentioned that every day we are, first thing to do is check the patient exactly as the baseline or is there change in the baseline, we have our tools to check if the patients are delirious or not then we gather if we need to go further or not, yeah.

**Interviewer:**

What do you do if an emotional or behavioural challenge presents itself from the person you are caring for? What is the type of action that is taken?

**Interviewee:**

Uh, if there is uh what challenge, sorry?

**Interviewer:**

Behavioural or emotional.

**Interviewee:**

We try to deal with it non-pharmacologically.

**Interviewer:**

Non-pharmacologically, what way is that exactly?

**Interviewee:**

We will try to understand what's the problem with the patient, what make him angry, what could be the trigger factor, if there is a cause that need treatment, we will actually address it if there’s any trigger we will try to avoid it and we will try to make it non-pharmacological, make the patient surrounded by familiar faces, trying to keep the patient safe, until we will treat the cause that is make this changes. If we feel or if the patient is really at a stage where he is at harm for himself or surrounding people or providers, at that time, we may go to give medication that calm him down a little bit.

**Interviewer:**

Psychiatric medications?

**Interviewee:**

Yeah, something like it.

Interviewer:

How would you, aggressive behaviour, would you say it’s always part of dementia or-?

**Interviewee:**

No, according..

**Interviewer:**

Is aggressiveness a symptom of dementia?

**Interviewee:**

No, it’s one of the complications of dementia or behavioural or psychological symptoms of dementia. Aggression is present in almost 40% of cases of dementia, so it's expected complication, as we mentioned, we are trying to deal with it and even to inform the family to deal with it non-pharmacologically if possible; trying to direct the attention, trying to see what's the things that making the patient aggressive, and if all these things fail then as I mentioned he start to affect his life or safety and the others safety, and these things we give medication for a certain period of time, reassess after a certain period of time, then remove it because all these medications is raising risk of mortality.

**Interviewer:**

Can you think of a time when you didn't know how to handle a caregiving situation, it was beyond managing?

**Interviewee:**

I didn't get you, do you mean the patient himself or somebody around?

**Interviewer:**

No, directly dealing with the patient and you couldn't handle the situation, emotionally or anything.

**Interviewee:**

Aggression or something else?

**Interviewer:**

Any of those challenges.

**Interviewee:**

Yeah yeah, yeah definitely I could remember aggressive patient who totally refused everything and he started to damage their place, and we saw that he really was at risk yes, and we sedated him with medication, we calmed him down with medication and it gave us time to learn what exactly was the reason.

**Interviewer:**

What ended up being the reason?

**Interviewee:**

I don't remember exactly what the issue was but for sure it was something, and we treated it. Infection or something like that, but even if it was nothing it gave us time to be able to change his medication and later on he can be corrected by medication and treatment. Yeah, yeah definitely some points are-

**Interviewer:**

So the end option is-

**Interviewee:**

Medication, yeah, yeah.

**Interviewer:**

Before that you try to manage? Okay. This is my last question. I noticed that not everyone here is an Arabic speaker and the staff is made up of people coming from different cultures like-

**Interviewee:**

You speak about the provider or patient?

**Interviewer:**

no, the providers, care providers. Have you ever made an observation where culture or observation was a communication barrier between provider and the patients?

**Interviewee:**

Yes

**Interviewer:**

Okay. How regularly does it happen?

**Interviewee:**

Okay. I cannot give actually a clear answer as to how often, because its not so often, because we are planned our care in this way, we try to keep an Arabic staff available in each shift to avoid this thing to happen; we tried and we started an initiative by teaching the non-Arabic staff basic Arabic language.

**Interviewer:**

So that's part of their orientation?

**Interviewee:**

It's an initiative, yeah, it’s an initiative.

**Interviewer:**

How long ago did it start?

**Interviewee:**

It started earlier this year and with time they know the basic things that we need them to..

**Interviewer:**

Yeah especially the ones that have been here long enough, they do pick up.

**Interviewee:**

But keeping one of the Arabic staff with the non-Arabic staff is the magic technique.

**Interviewer:**

If the person giving direct care to the patient is a non-Arabic speaker..

**Interviewee:**

They know how to communicate and assemble words but they face that there is a challenge they ask the Arabic staff - "can you please come and help us" and it works to be honest with you it’s not a big problem with it.

**Interviewer:**

It's not as big of a culture clash, let's say for example it's the day of Friday for example, I mean the Friday prayer is very common even non-Muslims know it but anything that is specific to the Emirati culture, for example, the care staff..

**Interviewee:**

No I didn't face it but I hear before that some of the patients were refusing to get care by non-Muslim staff long time ago, and according to that, because this is the patient’s right, as we say so we were trying to adjust his schedule with Arab Muslim staff but this was ages ago.

**Interviewer:**

It's not that common.

**Interviewee:**

Yeah

**Interviewer:**

Are all your patients Emiratis?

**Interviewee:**

No.

**Interviewer:**

Okay.

**Interviewer:**

And the in-patients, are they all-?

**Interviewee:**

We have an admission criteria for the in-patients, that the patient has to be Emirati but still there is exception to each rule, yes. For some of the cases, we are taking exceptions from higher authority for non-locals, so yes, definitely we have non-Emirati in-patients, yes.

**Interviewer:**

And, how, what percentage is that?

**Interviewee:**

Uh, comparing Emiratis to non-Emiratis, we could say 40-60, majority is for the Emiratis

**Interviewer:**

Is there anything that I haven't covered that you would like to share your thoughts with me?

**Interviewee:**

No at all, but I just want to tell you that if you any have any question race through your mind, at any time, you don’t need to make barriers you're more than welcome.

**Interviewer:**

Thank you so much.

**Interviewee:**

Anytime, and even I'll take your number right now because I'll add you to the Alzhiemer's support group and it will help you.

**Interviewer:**

A lot, a lot.

**Interviewee:**

It will help you. Just listen to them and if you need anything we can see what we can do.

**Male Nurses Interview**

**20 February 2024**

**Interviewer:**

Thank you all. Welcome everyone. I’ll start by asking what a typical day is like at work for you.

**Assistant Nurse 3:**

For dementia patients? It’s different every day. I have a patient, for example, sometimes he takes his food, sometimes he does not. Sometimes he allows for every personal carer to do therapy and everything. Like that, his mood swings.

**Assistant Nurse 4:**

Sometimes, his mood is okay; today, he is happy. When we are signing, he is repeating, sometimes he is very depressed. Whatever you say he will not be happy, you ask him to give me five dirhams he will not give hahaha.

**Interviewer:**

Right, yeah. But are you aware, that’s part of the condition that the dementia is affecting his brain that’s why he forgets.

**Assistant Nurse 4:**

Yes. Of course.

**Assistant Nurse 3:**

We know that.

**Registered Nurse 3:**

He has Alzheimer’s I think. He was a fisher.

**Assistant Nurse 4:**

Yes, before, he used to be a fisher. Vey difficult.

**Assistant Nurse 3:**

Very difficult.

**Assistant Nurse 4:**

60 years! 60 years!

**Registered Nurse 3:**

He's young also.

**Assistant Nurse 3:**

Very difficult, I mean not easy.

**Assistant Nurse 4:**

When he was still walking, it used to be more challenging.

**Assistant Nurse 3:**

Yeah more challenging like *patient name* used to be before.

**Everyone:**

Yeah, yeah.

Assistant Nurse 3:

Yes, see *patient name*. You see *patient name* when he was still walking, it was very difficult. This centre was awake. Really!

**Interviewer:**

Hmm yeah.

**Registered Nurse 3:**

All the back doors were locked. Because of him. Because we were worried he might run out.

**Interviewer:**

Hmm, yeah, you had to lock it.

**Registered Nurse 3:**

We used to have also *patient name* you remember?

**Assistant Nurse 4:**

That was the time we were running behind him.

**Interviewer:**

When was this, before covid?

**Everyone:**

Yeah, before covid, before covid.

**Interviewer:**

So that's what I mean; right now, we have only these many patients, but in the future, you will have other ones joining your care. If you have the knowledge on how to care for patients with dementia, it doesn't matter if patients come and go, you have the knowledge you know how to care for them. Right? you have the knowledge and the resources that you need so that the challenges you just described, God willing, in the future won't encounter them you will not have those kind of challenges again. Yeah?

**Assistant Nurse 3:**

Yeah, inshaAllah. What tests do they do to know it’s Alzheimer’s? For instance, in MRI, we can see something in the brain, yeah?

**Interviewer:**

Yup, you can, you can. But when the doctor gives you a diagnosis it's not just based on the MRI. They also give you questions to answer. Neuropsychological testing of cognitive functioning.Together with the MRI or other imaging techniques, they take everything into consideration, and then they make the diagnosis.

**Assistant Nurse 3:**

Then do you have any plan to [do research to] prevent dementia?

**Interviewer:**

Well, that's another project. My project is that once they get dementia, how do they care for them? Preventing dementia from happening is not part of my project, maybe other people are doing that.

**Assistant Nurse 3:**

Because now we are thinking about ourselves.

**Interviewer:**

Yourselves? InshaAllah, no, that won't happen.

**Assistant Nurse 3:**

Why I'm asking? You know, sometimes you know, yesterday my wife she called me 'Habib', then I told my kids your mother is starting Alzheimer's. They asked me why? I told them she called me Habib instead of my name, which she's forgetting.

**Interviewer:**

Hahaha.

**Assistant Nurse 3:**

Anyway, really, sometimes I feel about myself, sometimes I forget the names of other people. Now those who are working with me are asking me how do you know the names of all these people. I change my way, for example. I joke with them using their names or try to find similarities to Arabic words. I'm seriously telling you I'm using this way and will not forget.

**Interviewer:**

But that's a good strategy.

**Assistant Nurse 3:**

What do you guys think? That's a good strategy. To actually prevent dementia using your cognitive skills, trying to make connections and associations that will help you avoid or prevent dementia. Which is a good strategy, it's not a bad thing.

**Interviewer:**

No, it's a very good thing. So, InshaAllah, I don't know if ____ was supposed to come, but, um, we will start without him

**Assistant Nurse 3:**

He's very forgetful. He is starting to uhh.

**Interviewer:**

No, he said had to do something for one patient.

**Assistant Nurse 4:**

You should call him otherwise he will forget.

**Interviewer:**

No, so, InshaAllah, I hope you are all informed. Did I give you all the papers? Who has them?

**Assistant Nurse 3:**

And is it true that some food, if you start eating it, is good for dementia to protect your psyche?

**Interviewer:**

I wish I knew so that I would eat it myself. But uhh, I don't know. My project is not about the prevention of dementia. It's about what kind of care happens after it happens.

**Assistant Nurse 3**:

After it happens, uhh.

**Interviewer:**

Yes. So, how do I want to start this session is by asking you to share about one memory that you have about, hmm, caring for a patient with dementia. It doesn't have to be just one patient. It can be from the past like *patient name* for example that you mentioned. Just take one example from the past and tell me about that event, choose one challenging event or maybe a stressful event. If you could all share your experiences, just give me one example.

Assistant Nurse 2:

One example which I remember about *patient name*, actually he remembers his past things which happened 30 or 40 years before. He married from India, I think.

**Interviewer:**

Okay.

**Assistant Nurse 2:**

And he knows some places near to my place which is in Kerala, which street name and everything,

**Interviewer:**

Wow.

**Assistant Nurse 2:**

But at the same time, uhh, if some visitors comes for him. Some of his close family members and we asked him the next day, he forgets.

**Interviewer:**

Okay.

**Interviewer:**

Okay, yeah and how does that make you feel? As someone caring for him when you see him forgetting.

Assistant Nurse 2:

If you give him anything he forgets it.

**Interviewer:**

Okay. Hmm, right. And was there ever, like maybe just once, you found it very difficult or very challenging for *patient name* when caring for him? On even just one example you can tell us.

**Assistant Nurse 4:**

During the feeding time, it is very difficult to feed them.

**Interviewer:**

Okay, how? Tell us more.

**Assistant Nurse 2:**

Whatever we tell him, he will not open his mouth.

**Interviewer:**

And what do you do?

**Assistant Nurse 3:**

As per his mood, we will try to feed him.

**Interviewer:**

And what happens if he doesn't open his mouth? How do you deal with it?

**Assistant Nurse 3:**

We will wait for some time, we'll talk to him.

**Interviewer:**

Okay.

**Assistant Nurse 4:**

And then we'll ask for help from the Arabic staff, who will then speak to him in Arabic.

Interviewer:

Okay.

**Assistant Nurse 3:**

If he listens he will open his mouth, we will feed him something.

**Interviewer:**

So to you, the most challenging part is when he is not cooperating during feeding time. Would you say that's the most challenging time?

**Assistant Nurse 4:**

Yeah, that's it.

**Interviewer:**

I guess *patient name* is a very unique case. he is not always angry or something, so he's okay. He's easy, maybe?

**Assistant Nurse 3:**

He's easy yeah.

**Interviewer:**

Okay.

**Assistant Nurse 4:**

Sometimes he forgets to swallow.

**Assistant Nurse 2:**

Yeah, that one was very difficult. He will forget to swallow.

**Assistant Nurse 4:**

He will keep the food in his mouth, then you have to remind him, 'Baba, swallow, swallow'. You have to remind him, like in between.

**Interviewer:**

Uh.

**Assistant Nurse 4:**

Then, after that, he will hear that, and maybe he will start chewing the food and swallowing.

**Interviewer:**

Right, right.

**Assistant Nurse 3:**

Also recently, now he likes hot water, we are making hot water. We are making hot water, and during bath times, he says cold, cold!

**Interviewer:**

Aww.

**Assistant Nurse 3:**

He forgets the difference between cold and hot.

**Assistant Nurse 4:**

Before he was giving me, umm, I was joking with him. I'm asking if he want cold or hot; he said hot, then I said sugar or no sugar. He forgot what I was going to give him but he was telling me with sugar.

**Interviewer:**

Right.

**Assistant Nurse 3:**

Tea or coffee?

**Interviewer:**

And after you give him coffee, will he ask for tea?

**Assistant Nurse 3:**

No I was giving him a bath, but when I am preparing the water I am asking him you want cold or you want hot? And he's telling hot, and then I said tea or coffee.

**Registered Nurse 3:**

We were just talking about challenges, problems, and difficulties that you face when you are caring for patients with dementia. Do you have one example? One example.

**Interviewer:**

You know who was from the male patients, someone who passed away in, uh, October.

Assistant Nurse 4:

Uhh, *patient name*.

**Interviewer:**

Uhh, he had dementia; how was your experience working with him? Let's, let's uhh, I think *patient name* we have covered enough. Let's talk about *patient name*, how it was.

**Registered Nurse 3:**

Who is *patient name*?

**Assistant Nurse 3:**

*Patient name*, the one who was in Room #3.

**Assistant Nurse 4:**

Bedridden also.

**Assistant Nurse 3:**

*patient name*, the young one.

**Assistant Nurse 4:**

He was handicapped.

**Interviewer:**

Yeah, but he also had dementia.

**Assistant Nurse 3:**

Alzheimer’s.

**Interviewer:**

Alzheimers, yeah. Yeah, tell me how was your experience working with him?

**Assistant Nurse 3:**

He was in bad position. He was not talking. When he came, he was better; for example, when he was hungry, you will put the food in front of him he will not eat, he smells it, but he will not eat it.

**Assistant Nurse 4:**

Not talking.

**Registered Nurse 3:**

When we feed him something he will talk, some sounds he will make.

**Assistant Nurse 2:**

We don't know.

**Interviewer:**

Yeah, I didn't get a chance to work with him, when I started, he moved into the observation room, it was too late.

**Assistant Nurse 1:**

According to his relatives he was playing football before

**Assistant Nurse 4:**

Yeah, he was going to the club.

**Assistant Nurse 2:**

When he was admitted here, he forgot everything, only the things he was persistent with before and uh always.

**Assistant Nurse 3:**

He liked soft voices. We would always bring him other nurses.

**Interviewer:**

May he rest in peace.

**Assistant Nurse 3:**

Only he recognised his parents, he would remember them.

**Interviewer:**

Did they use to visit him?

**Assistant Nurse 3:**

Yeah, his parents came in once a week, twice a week. I remember his parents.

**Interviewer:**

Oh MasaAllah, okay. Tell us your experiences of caring for dementia patients.

Registered Nurse 3:

*patient name*.

**Interviewer:**

Yeah, you told me when *patient name* used to walk it was very difficult can we discuss that, how was that like.

Registered Nurse 3:

He didn't know which way he was going only he was looking for that open way, example, he will come here he will try to open the door, and if it's closed he will turn right, left or maybe, and just walk and walk. One day we went global village, we took him with us, and he ran all through global village.

**Interviewer:**

And did you run with him?

**Registered Nurse 3:**

No, no, another nurse went with him, poor thing.

**Interviewer:**

But how do you manage such situations? For example, let's say tomorrow there is a new patient who has Alzheimer’s, they come and they are like *patient name* before, they keep on walking. How do you manage that?

**Assistant Nurse 4:**

We'll call *other* nurses [for help].

**Interviewer**:

Assistant Nurse 2, you have something to say? How would you handle a patient who is not bedridden because now all patients are bedridden, so you are saying even for *patient name* for example bath time is very difficult because he can be heavy. What if the patient is not bedridden, what if they are walking like *patient name*, they try to go out, or maybe they are aggressive? Typically, how do you manage such situations?

**Registered Nurse 3:**

I think all of us know; you just think about the needs they may need. We plan according to that.

**Interviewer:**

Okay.

**Assistant Nurse 2**:

Especially when they are forgetting something, uhhm, that they need to use, we keep everything next to them, when there is no carer and everything we have our own timetable here, bath, feeding time, everything.

**Interviewer:**

The routine?

**Assistant Nurse 2:**

Yeah, routine is there, so when they wake up, it is according to our time routine and everything.

**Interviewer:**

Yeah.

**Assistant Nurse 3:**

If they are wandering, we will think about things we practice here as staff will escort them back, and things like that.

**Interviewer:**

Yeah, okay.

**Assistant Nurse 4:**

We should also read about their past because we should know about what happened to these people at home, always you are repeating the name. For example, we know the name of his son we say how is he, and he says he is fine. Always remind them of this.

**Interviewer:**

Okay, so you bring topics from their past and try to engage with them that way?

**Assistant Nurse 4:**

Yes.

**Interviewer:**

Hm, okay. Assistant Nurse 2, I know you have joined very recently, what has been your experience working with patients with dementia?

**Assistant Nurse 2:**

The difficult thing for me is eating and bathing times.

**Interviewer:**

They don’t cooperate? Okay? I think it's common, these challenges are very common.

**Assistant Nurse 2:**

Anything in the past or old he won't remember, you'll feed him and bathe him and he won't remember he'll say I still haven't eaten. Other times, he'll remember really old things that happened 60 years ago, like the day of his wedding or old politics. The new things, however, he will not recall.

**Registered Nurse 3:**

He can remember the past but can't maintain his memory short-term.

**Interviewer:**

Yeah, that's part of the illness, that’s part of the condition.

**Assistant Nurse 2:**

It's an issue. He'll be eating, then right after, he'll say they haven't fed me; what do you mean they didn't feed you? If someone else comes in unaware, he'll think, why did you guys not feed him? Meanwhile, he's been fed not but a moment ago. And what else? He likes to keep walking; he doesn't like to just sit around. And you may tire walking, but he won't. He would walk up and down, very strong.

**Interviewer:**

MashaAllah.

**Assistant Nurse 2:**

He would walk, twist and turn with you.

**Assistant Nurse 4:**

*patient name* has lots of stories. We would take her outdoors and we leave her walking, if she was walking we would try to make the environment safe so she would not hit or fall, so she was roaming around and around, and walking. Even at night. That’s very difficult.

**Assistant Nurse 3:**

Even sometimes maybe dirty food and things like that, he will eat, and we say, Baba what is this, why?

**Assistant Nurse 4:**

Oh, I remember.

**Registered Nurse 3:**

Remember when we were feeding him; he would not stop. We had to stop.

**Assistant Nurse 3:**

He will not stop.

**Assistant Nurse 4:**

He will not stop.

**Interviewer:**

Okay, so I have a question when that happens, when they don't stop eating or like when you said when you feed them but they forget and they say I haven't eaten anything. Do you or are you trained to know that this is what dementia does or do you think something is wrong with them? What is your conclusion? When a patient shows you some symptoms like that.

**Assistant Nurse 3:**

No everybody just knows. After a certain age he just forgets everything. But even at 90, when he starts reading a Quranic verse he finishes it. He remembers it.

**Assistant Nurse 4:**

I remember this man, because he was talking about, he was very old. Long time ago he was an Imam of a mosque. No, but that man he's very, very difficult.

**Interviewer:**

Difficult? Where did he go? Did he pass away?

**Assistant Nurse 4:**

He passed away.

**Registered Nurse 3:**

That, you know when he was reading he was reading for hours, and when he wanted to talk he'd want to talk about the Quran.

*------Interview interrupted due to emergency involving a patient-----*

*------Interview restarted but with just Assistant Nurse 2--------*

**Interviewer:**

“I will just take out some of the questions… ok. Can you please tell me a little bit about your position as an assistant nurse and how are you involved in the center? What are you doing, what are your roles?

**Interviewee:**

“Yes, actually, we are taking care of the patients, we are doing morning care, especially morning care, giving bath, nail cut, shaving, diaper, everything. And we are feeding them; we give them the snack. We are also taking them sitting outside as you see that older people they are sitting there in the tv room. Because they are having activity. Social activity, physiotherapy activity. From there, after that bath they are clean and tidy, the…eh …our...ehm. Physiotherapy starting also with them, the same program, and the timing. And we are bringing them here sitting all together, and some visitors sometimes came, same today also. We have visitors came today and they are sitting with them, talking with them. Ah…em…In the afternoon we return after they have lunch. We return them to their rooms. In the room also we observe them, and we change the position for them, diaper, ehm.. Keep the room also is clean and ehm temperature good temperature for them and if they need anything, they will call, and we will eh ehm enter the room. Almost all we are doing. Taking vitals and with them helping dressing done with the staff nurse. Sometimes also, they go outside; this is ehm.. trip! We are…they are going, they are selecting one or two from the patient. also, we are going with them As escort! And also if they have appointment or they have an x-ray in our center or any hospital we are also going with them as escort. This is mostly what we are doing with them.”

**Interviewer:**

“And do you talk to them?, Do they talk to you?”.

**Interviewee:**

Yes, some are able to talk with you and they are answering, we are sitting laughing, we are err yes, this is part of our work. We should talk with them because ehm we are feeling that this is same as our family, just put yourself our erm, I am putting myself in my home when I’m sitting my home and my kids all they are busy with the devices, and they are not talking with me I feel bad, for that one I am sitting here. In between we sho... we are talking with them because we don’t want them to feel also as what I am telling you.

**Interviewer:**

yeah, and do they feel happy when you chat with them?

**Interviewee:**

“Yes, it’s both sides. I feel happy, really and I think also they are feeling happy, because they are laughing some... erm... some they are happy. We are, you know, taking them to other places, we don’t want them to feel here in the same, the same one place and they are not going outside, and they are they do not have any relative, some like that... Always we are talking with them, yeah.

**Interviewer:**

That’s good, Masha Allah. Ehm, what do you talk about?”.

**Interviewee:**

“Generally, sometimes we are talking about, if they have, we know, if they have kids, or what they’ll they love for example; what did you work before, almost we know this is they are what is going for the sea and bringing fish and there...ehm yeah, we are talking about this one. Sometimes we are talking about the ehm...that each other. Oh, *patient name* here if he catches you, he is sitting there, he is our patient, yes, and we know ehm...from the experience, what they love. We do what they like and what they love, yeah. That’s ehm. Some jokes, we are joking with also, like that.

**Interviewer:**

Do you know any one not in the centre, but maybe from family, from friends who is diagnosed with dementia?

**Interviewee:**

Usually no, only that some outpatients come from outside, we know, with escort, we know they have ehm Alzheimer’s, and, from my side, no, from my family no.

**Interviewer:**

Even friends, family, relatives?

**Interviewee:**

No

**Interviewer:**

Ok, so your experience from dementia is only at work?

**Interviewee:**

At work, yes

**Interviewer:**

And, ok, so from ehem the inpatients, how many of them are you aware, have dementia, do they tell you; the doctors; who has dementia, or you’re not told?, let’s say for example out of the inpatients now, who do you think has dementia or do you know that if they have dementia?

**Interviewee:**

In the present, I don’t think anyone has. Ehm… I don’t think anyone has.

**Interviewer:**

Ok, tell me…”

**Interviewee:**

Yeah, Sorry, as I told you the doctor is the one, he’s telling ehm… they go, they do the diagnosis and… also when he comes inside. We know that you know, if you ask something, things from the conversation.

**Interviewer:**

You look for the signs. Do you ask the doctor?

**Interviewee:**

Yeah, but after -of course- we will ask the doctor, when anyone come, we should take all the study and we read about this patient to know what is happening, how to deal also with him.

**Interviewer:**

Tell me, what do you know about dementia, Alzheimer’s; Alzheimer’s is one type of dementia, what is your knowledge about it, just generally, what do you know about dementia?

**Interviewee:**

The first is that damage in the brain, it’s damage in the brain, and, also is coming according to the age, above 60,65 ehm… that’s what I know, about this, from brain damage and from age, it’s coming from age. Yeah.

**Interviewer:**

Ok, so, everyone who reaches that age will get dementia, or?

**Interviewee:**

No, not all, some.

**Interviewer:**

Only some?

**Interviewee:**

And very few, also not all.

**Interviewer:**

Ok, Describe a good day and challenging day for me at work?

**Interviewee:**

I told you when we are going with them outside. We are feeling happy with them because also, we are ehm…we feel they are happy, they are going outside, they are ehm…looking for the other place, they are eating different food, they are talking, they are, remember also, they, you know, sometime, we have one patient, when we are going, for example; he’s from RAK. When we are going RAK, he’s remembering area and he’s talking about this so, and this time and this one, we feel happy because, they are able to talk and remember everything about the old day, and sometimes, you know, this is the age, it’s coming the same like kids they are, sometimes they are fighting between each other, yeah, this one he’s reading Quraan, this one he likes to listen to the music, and like that.

------ Recording Interrupted------

**Interviewer:**

You were telling me, when they fight each other?

**Interviewee:**

Yes, they are you know, same I told you, like kids. They are fighting when they are sitting together, and telling; this one says no, no, and is telling that one. So [our job is to tell them], no, he is good, he likes you, like that! In the end they will forget that fighting, so many things like this. They make me happy when I am going home (ya3ny)…Alhamdulillah.

**Interviewer:**

Is the fighting stressful?, does it stress you when they fight?, sometimes maybe it’s too much fight, Do you worry, or is it challenging?

**Interviewee:**

Yes, for example; Yesterday erm…we have one patient he’s very nervous and he’s erm…he doesn’t like that you talk with him, he’s telling. He’s telling me bad words, but we know he’s not telling him such things. But why he’s giving him that man, we don’t know why then he’s shouting, we are afraid because he’s having hypertension, he’s having heart disease. If he’s shouting and he’ll be angry, maybe something will happen to him, and erm... how will we talk with him then we, the other patient, we take him a little away, and we speak with him. At least we solve the problems. Alhamdulillah, because we don’t also want them to fight and it’s not good.

**Interviewer:**

They say that if they come they don’t want to go.

**Interviewee:**

Yes! because here alhamdulilllah I think they all are happy because, they see how they are dealing, even the staff, the same as our family. All, not me only, all the staff; admin, nursing, doctors, all.

**Interviewer:**

MashaAllah. You told me the happy days, what is challenging to you as an assistant nurse, what are some things that are difficult for you and you say today was very hard day?

**Interviewee:**

yeah, it happens sometimes, when you can’t speak with them and you cannot make them happy or cannot change the situation, like that, but I think this is normal.

**Interviewer:**

They are not in the mood. Yeah, that’s true.

**Interviewer:**

Are all your patients Emirati? Arabic speakers?

**Interviewee:**

Yes

**Interviewer:**

Because you are Arab and you speak Arabic, communication is east with them, yeah?

**Interviewee:**

Yes, yes.

**Interviewer:**

What is your observation about staff who do not speak, let’s say for example nurses from India, the escorts or the assistants from Pakistan or Bangladesh?

**Interviewee:**

This is a challenge.

**Interviewer:**

A challenge for them?

**Interviewee:**

Challenge for them, yes, sometimes also sometimes, happen miscommunication also, because you know erm…this patient also not able to talk only Arabic, also it is difficult, you need something that ehm…maybe they will not understand and you will give, for example, if you give water but he doesn't want water, he will got angry, because they don’t understand and they don’t know how to explain to him.

**Interviewer:**

So language is a problem, it’s a challenge?

**Interviewee:**

uhm yes.

**Interviewer:**

And the patients get angry if they don’t feel understood?

**Interviewee:**

Yes, not all, but some if they are coming and they do not understand but alhamdulillah, I think even Indian staff or non-Arab staff also is, they are, because long time also they are have also they know, some they are coming from Saudi Arabia, they can understand some word, simple words, they can understand.

**Interviewer:**

yeah, talking about their children that’s not possible, for example. Is that what you are saying?

**Interviewee:**

No, no, no. Only simple words, and like that.

**Interviewer:**

So, would you say people you care for, the male patients, ehm…they are involved in making a decisions, let’s say for example, if you, you know, if in their schedule you have to go and give them a bath, but they don’t want to take a bath, you allow them to make decisions, or what happens in such situations?

**Interviewee:**

Yes, we will try, ehm…We will try to convince them. For example, we will give medicine, it’s good for you , but in the end if he doesn’t like we will not force him, because you know we know this is human being, sometimes I’m not eating one meal when today I’m eating one meal, nothing will happen, but if you will force him, maybe it’s not good, next time if I will come, maybe he will not accept me, better to give him the choice but we will try.

**Interviewer:**

And what about their time of sleeping, is it fixed? they have to go to bed at a certain time, maybe, let’s say 9 pm or 8 pm, and if they say “No, I don’t want to sleep”, what happens?

**Interviewee:**

yeah, we have, we have one, two patients they are not erm… they don’t like to go [to bed] in the exact time, yes according them because sometime they are going early, sometimes they are going late, sometimes in the between, they are sleeping and they wake up because when we are night duty and they are coming sitting with us in outside, we also this is you know, this is not easy to, to put them in the fixed time. Some of them are also bedridden. We are the ones, we are positioning him and putting him. So when we are going there, if they are not asleep, we explain with them, ah how are you ? why you are still wake? You need to sleep, to wake up early tomorrow, you have activity, but ehm…..

**Interviewer:**

No Forcing?

**Interviewee:**

No, but it’s also its out of their hands also cannot.

**Interviewer:**

When they don’t want to take medications, what happens?

**Interviewee:**

No, the medication is very important, it’s not the same as what I told you this we cannot also force them but will explain to them sometimes not agree we are calling the social worker. Social worker also they are helping us for these things. They are helping us also we are calling them and they are helping us also, sometimes [even] they cannot convince them.

**Interviewer:**

What about some of them, who have, let’s say money in the bank. Are they allowed to make decisions about their money? Let’s say for example they want to out money from the ATM and they want to go shopping, what happens if it’s not on the schedule?

**Interviewee:**

Yeah, it happens here, if they have money or he wants to go back, and even sometimes he wants to do something outside, we are going with them and they are taking permission from doctors, or from our director. We have these. Patients who always wants to go for shopping, they like to buy perfume clothes, everything even here they are providing everything even he still he wants to spend money.

**Interviewer:**

Do you think from your experience uhm patients who have dementia are happy or unhappy when other people make decisions for them or when they make the decisions themselves, which one makes them more happy, let’s say for example one patient he wants to go to bed at midnight but you know, the doctor had said he has to go bed early, when is the patient happy, is it when you tell them no, this is not good for you and then you convince them and then they go to sleep or you tell them, ok you can wait until midnight to sleep - which one makes them happy?

**Interviewee:**

When you are giving him the choice.

**Interviewer:**

Tell me more about that.

**Interviewee:**

For example, you will explain and he will tell no, I don’t want to go now, I want to sit for a while. Maybe he will sit five minutes then he will go, but at least he did what he want.

**Interviewer:**

Aha. So you will not let him wait until midnight you will just give him five minutes?
**Interviewee:**

Yes.

**Interviewer:**

Clever.

**Interviewee:**

Yeah, yeah the choice is better.

**Interviewer:**

What do you do when sometimes they get very upset, all of a sudden like the person you mentioned in room 13, they think you know erm…*patient name* was saying something but in reality, he didn’t and they were upset, what do you do when they get too upset and it’s very difficult to calm them down what, what do you do?

**Interviewee:**

First we will try to call the social workers erm…sometimes we call the doctor, yes of course because sometimes you know you can feel this people need a dose or change some medication. If the behaviour suddenly changes, why is he changing? Why is he saying bad word? And he is not telling why? Easy to get angry this means there is some problem saying, we should tell the doctor and the doctor again maybe he will check what is the medication he’s taking, they are taking psychiatric medication or hypertension medication mostly, maybe they will change the medication or they will give a different dose like that, it’s to be stable, the situation.

**Interviewer:**

Usually it is related to medication, you think, their behavior changes?

**Interviewee:**

Not all, but the some, yeah, especially if it’s psychiatric and he’s taking for example that he has a pump example with a 5mg medication, when we see he is changing or is becoming difficult to deal with him we will tell the doctor, the doctor will give him 10mg, then he will be okay, the same happens with hypertension, sometimes we are telling the doctor that hypertension is 140 over 80, this means he might give you a little dose uhm.. and the medication is to control this hypertension. So like that.

**Interviewer:**

Ok, other than medication is there any other reason why they get very upset or sometimes, you know they become restless?

**Interviewee:**

Yeah, I told you because it’s not easy, also here for some people not all but, for example, *patient name* because you know he is sitting, most of the time he is alone and he’s, he doesn’t have family, no wife no kids, no relatives, and sometimes… For some people it is hard.

**Interviewer:**

Yeah, ok, so, your strategy when they get upset you told me first, you try to calm them down, and then you call the social workers when it becomes too much. Is that so?

**Interviewee:**

And also we will know, what is the reason the first one is what is the reason…I will not talk about this case yesterday because…difficult case, but example, before we had one patient, sometimes he is shouting and calling, and when we are going there, first we want to know why, what is the reason why you don’t like this one, no, ok fine, will change, like that we will remove the reason of that…ehm.

**Interviewer:**

and they become ok?

**Interviewee:**

Yeah

**Interviewer:**

Is there anything else you would like to tell me? I have finished my questions.

**Interviewee:**

Yes, about Alzheimer’s or in general?

**Interviewer:**

yes Dementia, Alzheimer’s.

**Interviewee:**

yes Alzheimer’s, we should, when we are dealing with patients, we should know that this is not, they are not changing their behaviour because they mean to do that, no, [it is] because they have a problem and that’s, we have to accept them as what they are doing we have to be patient with them because it’s part of my work and this is, he’s the patient, we should accept them and we should be patient with them, and we should take care of them. That’s it.

**Interviewer:**

Thank you so much.

**Female Nurses Interview**

**22 February 2024**

**Interviewer:**

Ok. First of all, thank you so much. I know you're very busy. I've already explained the purpose of today's meeting. My presence here is to just guide the conversation but I would like you, you four to engage in the discussion. Um we can start by asking to share one experience you've had working with a dementia patient in the past, recently. It can be with anyone, even from your past. What was that like? Just pick one example, one memorable experience, and then just share that with us.

**Registered Nurse 1:**

So your research is regarding dementia, right? Uh…if you're talking like in the early stage, it's very difficult to handle the patient. Patients who forget the present things, but they remember the past things. Like say maybe ten, fifteen years back, what he was, what he did and everything he will remember. But the present things, he will forget, his position, father, mother, relatives, and everything, they will forget. And they will be little aggressive because there is a…uh, drastic changes in the chemic…you know, the chemicals in the brain, so this we can see in dementia patients, early stages of dementia is difficult to manage, yeah.

**Registered Nurse 2:**

Uh... then, like chronic patients who are here like *patient name* know. What are their basic needs, their needs, we know for those kinds of patients, because we know. So, things like maybe when they in pain, or something else, we can act, and we get, uh, we know that the patient is in pain by their facial expressions. Other things, we know, as in us, we know what that patient needs. That we wiltl do from our side. But, one thing, [caring for a patient in the] initial stages of dementia is very difficult. he difficulty is in the initial stages of a dementia patient.

**Interviewer:**

It's more demanding?

**Registered Nurse 1:**

Yeah, more…it's like uh, uhh, they're very aggressive behaviour.

**Interviewer:**

Ok. Can you think of one example of, of dealing with a patient that was the most challenging to you? Do you remember a time?

**Registered Nurse 4:**

Yeah, there's one patient, he was ambulatory, he was walking around. Because he, you know, there are so many medical equipment, so if we just leave his medications and everything there. He will carry, if he sees a mobile, he will keep in his pocket. So even he will keep in his pocket, everything, even syringe...

**Interviewer:**

Who was that? I don't think I've met him.

**Registered Nurse 2:**

He's not there anymore.

**Interviewer:**

He passed away?

**Registered Nurse 1:**

Yeah, so always, one staff should be there with him, all the time.

**Interviewer:**

Ok. Was there ever an incident that took place where you couldn't you couldn't manage?

**Registered Nurse 4:**

Yes, sometimes they will become very aggressive, that time doctor will give maybe tranquilizer, antidepressant medications, so it will suppress their symptoms. Especially their symptoms that develop when the sun set.

**Interviewer:**

Hm, the sundowning, right. Yeah, what do you do when they feel distressed during, um, when the sun is going down and they feel in distress? What do you do?

**Registered Nurse 1:**

Yeah, for such…that stage of patients, always there is one staff with always with them.

**Interviewer:**

What actions do they take when the patient is in distress?

**Registered Nurse 1:**

First action we have to take is the prevention of fall, we have to take them, because there is most chance for them to fall, because they don't know what they're doing. So we'll always take care of them, uh, from fall. Then, their needs, whether they're eating properly. Sometimes if you're telling two, three times only they will take some food.

**Registered Nurse 2:**

And also sometimes they will forget to swallow, keep inside mouth and uh…

**Assistant Nurse 1:**

They will keep it in their mouth…

**Registered Nurse 1:**

Medication and all, we have to make sure whether it's taken.

**Assistant Nurse 1:**

The most challenging time is while feeding, you know, the patient is not quick to swallow. For me, that is the most challenging.

**Interviewer:**

Okay. Which, which is it? Feeding?

**Assistant Nurse 1:**

Feeding, yeah. Because, it may affect the aspiration of...the patient.

Interviewer:

Yeah.. You worry that they might choke. Okay.

**Registered Nurse 2:**

Uh, I can tell from experience from my mother itself.

**Interviewer:**

Okay.

**Registered Nurse 2:**

It was since, uh, after her, she had two surgeries, then after that she became demented. Actually, there is, your study about this also. Is there any family history, uh, it is hereditary or not? Because my, my mother, my mother's both brothers, both had dementia and um, they passed because of all this complication of dementia, and then…After that actually, she started slowly, slowly, gradually, actually. At first, it was showing like, mm, little repeating. Repeatedly, she was asking the questions, and I understood that this was dementia and initially itself I started with some homeopathic medicines. And once, you know, she was so aggressive in the house. We were not knowing what is the reason, there is no specific reason, then, we were not able to even take her to the hospital. I was here only, and my younger- she's staying with my sister and her family, and she was talking very aggressive, going to beat my sister. Many things. Many things means they cannot control her. Then finally, I was, I was thinking what to do in such case. Then we called the ambulance also, okay. At least ambulance can take her and, take her to the hospital. Then what she did, she was not...she went inside the room and she locked the door. Then, hmm…they tried to go inside, then she was…uh…she was…there was some iron rod, and she was holding that and she was going to beat them. So, nobody came inside and take her to the hospital, then, finally the ambulance staff left. Then finally my son was calling. Then, her one grandson is there, she loves him very much. Then, we were trying to tell him just, to make her calm and tell her very cordially and…ask her, ask her loud---loudly, loud- very loud what she wants, then finally we convinced her and we have taken her to the hospital. Then after that they started giving…like uh, quetiapine and antipsychotics, then antidepressants also. And then after a few days, she is a bit okay. After that we brought her to the house, and she is okay now. Still here. I'm continuously giving this homeopathic medicine, but she is maintaining with that. After that, continue with this homeopathy, and…it's okay, with that. It can at least maintain that stage, but in between, she becomes irritated, aggressive.

**Registered Nurse 2:**

And…and another thing about the dementia patient is that, even if we are giving, some days, maybe they will forget, so they will go and ask food, say "nobody's given food." There are so many complaints from the patients. "Nobody has given food," "I don't have anything." Like this, and, uh, the caregiver or the family members, how it feels, it is very, very…very sad actually.

**Interviewer:**

Yeah, it hurts…Yeah. Thank you so much for sharing with us about your, I, I know about your mother's situation and, um, it must be so difficult for you and your family to handle that. Yeah.

**Registered Nurse 2:**

Yes, very difficult.

**Interviewer:**

So, thank you for sharing that. It is part of the illness, it is part of the condition that patients might show, such changes.

**Registered Nurse 2:**

And also, Alzheimers, you know, they will not remember the old things. Sometimes my mother, every time she starts.. She never asks where is her husband, okay. But where is my Amma, where is my father, where are my brothers, why, I want to see them, I want to go to them. Every.. Then I used to ask then, "Amma, why are you not asking about your husband", I mean my father, then she says..."I thought that he, he went for work”. Like that.

**Interviewer:**

Yeah, it's quite different from one patient to another as well, what they experience. Here in your centre, we have different patients. *patient name* for example has younger onset. *patient name* has Alzheimer’s, um. *patient name* and *patient name* have vascular dementia which happened after their stroke, and their symptoms are quite different as well. One question, I mean, one thing I'm curious about is.. When you're dealing with patients like these, for example, I know *patient name* from going through her archive, um, records, I know she used to walk, and she was in a, in a much different condition when she was first admitted. How was it? How was that experience like?

**Registered Nurse 1:**

She was running like anything…

**Registered Nurse 2:**

Many times, I think two or three times, she fell down also.

**Interviewer:**

And how long did that continue before she settled? When…Before they put her on medications?

**Assistant Nurse 1:**

Three, four months. Four months, no?

**Registered Nurse 2:**

Yeah, yeah.

**Interviewer:**

So for four months, on a daily basis, that would happen?

**Registered Nurse 2:**

At night she was not sleeping. She was roaming all patients’ rooms, she's- because she doesn't know her room. So, she is going to, some another patient's room and uh.

**Interviewer:**

And how was that like? Did one of you have to be assigned, at, at all times?

**Registered Nurse 1:**

yeah yeah yeah.

**Registered Nurse 2:**

Mm yeah yeah. Because..because we don't want an incident from others, they will complain. That they will not think that this the patient. Uh. And she used to go to.. One thing she went to my patient’s room and she was going to trying to, go into the bed. And it was, and it is, it was very difficult, she was very strong, very difficult to remove her from the…so.

**Registered Nurse 1:**

Initially, actually, there are several stages for dementia. So, initially they will…say Alzheimer’s.. Initially, uh, before we have one patient. Let me see if I remember, *patient name* Yeah.

**Interviewer:**

Oh, *patient name*.

**Registered Nurse 2:**

Her, she was…she will talking, talking, talking then, she used to go around the centre and she will pluck the flowers and she'll say, "This is for my mother." Then, uh, and like that she was walking, walking, walking, then, fine after some time, they were completely bedridden, then, it's like..

**Interviewer:**

So, it's in stages, and, as Registered Nurse 1 pointed out, the initial stages of dementia, you find it the most difficult. Is it because of the physical handling, or is it because of, um, the barriers in communication? You don't know what they want, so you don't know what to do for them? What aspect of initial stages of dementia do you find the most challenging?

**Registered Nurse 2:**

Actually, physical handling also, it is difficult, because they are, they will not sit. They, they are not to be tired also. They are walking walking walking and the one who is taking care of the patient…it's uh, it's really, it's very difficult. They will not listen. They will not hear what we are telling and you know. They cannot think anything.

**Registered Nurse 1:**

Physical handling…

**Interviewer:**

What do you, for example, let's talk um about *patient name*, cause she's a good example of someone who transitioned through the stages.

**Registered Nurse 1:**

For her, you know, when he came, she was always talking about her sons. Always to everyday she wants to see them. Now you see she is not talking about them. She…she's not telling anything. I think she forgot, yeah.

**Interviewer:**

It's actually, her condition is, yeah. Whenever she, she was roaming around at night, when you tried to tell her and she doesn't listen, what was it that you were telling her? Is it "come back to your room"? What were the things that you were communicating with her?

**Registered Nurse 1:**

Actually for them, we have to maintain, they're telling we have to make good eye contact and we have to repeat three, three times.

**Interviewer:**

Okay. What do you repeat?

**Registered Nurse 1:**

Then maybe they will catch up later. Then they will decide, and slowly they will come. Just one time if you are telling come, come, come to your room, this is your room, they will not listen. You have to tell at least three times.

**Registered Nurse 4:**

And that time, she likes male staff also, because maybe…she is remembering her sons, maybe she missed them, you know? So she likes that, like male staff, so whatever they are telling, she is listening there.

**Registered Nurse 1:**

Yeah. Like that she was listening to male staff.

**Interviewer:**

Okay. That's interesting. *Assistant Nurse 1*, how long have you worked here? you and I never cross paths, I think when your shift is here, I'm not here!

**Assistant Nurse 1:**

Only three months.

**Interviewer:**

Three months? Okay, so you're new. Yeah.

**Assistant Nurse 1:**

Yeah.

**Interviewer:**

How has it been for you here?

**Assistant Nurse 1:**

Actually when I came, that time *patient name* was, you know, on medication, so she was quiet, so I did not see. I heard from them, that she was like this, but I never saw her in that way. When I came, that time onwards, she was laughing, smiling, like that. Different behaviour.

**Interviewer:**

And what about *patient name* and *patient name*, since they're very similar in terms of their conditions? What, how is it like, caring for a patient like them?

**Assistant Nurse 1:**

*patient name*, she was talking, talking.

**Registered Nurse 2:**

*patient name*, she was talking, I told them last time. She was talking, and after her second stroke, yeah, she became…When she came, she was talking. After her second stroke only, she became quite.

**Interviewer:**

Is the care you provide different for when they are verbal? Is it easier, or more difficult when they are verbal?

**Registered Nurse 4:**

Now, it is easier.

**Interviewer:**

It's easier now? Okay.

**Registered Nurse 2:**

And because they, they are…they don't have, they cannot communicate. This patient, you know, she was resisting to do something. And whatever we are doing, it means they are satisfied.

**Interviewer:**

How do you know when they are not satisfied? What are the signs you look for?

**Registered Nurse 2:**

*Patient name* I can tell you, you know, she will-- when I, we used to go for the dressing, she doesn't like to remove her hijab, she will like to "Mm, Mm." Some noises she will make. And, and if she wants to, I mean, she used to tell, when we are talking, she used to, she wants to tell many things. When I, I feel like that. She wants to tell many things , but "Oo, ou, ou, ooou." Like this, she will do, she will try to tell right, no, *Registered Nurse 4*? But we cannot understand what she's telling. And she can understand, that inside, we, we are talking something. When we are trying to take her off this gown, she will…she will refuse. She doesn't like to feel more exposed.

**Registered Nurse 4:**

And she will get angry also, sometimes.

**Interviewer:**

So, she has expressions that are definite…Yeah. What about *patient name*?

**Assistant Nurse 1:**

Yeah, yeah. She has expressions.

**Registered Nurse 4:**

Yeah, yeah.

**Registered Nurse 2:**

*Patient name*...No. She's not there. Just a look, that's all.

**Assistant Nurse 1:**

*Patient name* nowadays…she's not…when we are talking also, she will just…But she is looking, sometimes when I am holding her hand, and telling "Momma" she's looking like that. When we are calling her name, and uh, holding my hand to her hand, she will.. she will catch and she will look at me. You know, that means she's, she knows me.

**Registered Nurse 2:**

Yeah, she will catch.

**Registered Nurse 4:**

She cannot express, but she knows that we are staff. When her son comes, he, she knows that's her son.

**Interviewer:**

Okay. How, how does she know? She's not communicating. How do we know that she is aware?

**Registered Nurse 1?**

We can understand from the face.

**Interviewer:**

Okay. When they are in pain, is there a way for you to understand that they're in pain?

**Registered Nurse 2:**

Yeah, especially since crying is familiar.

**Registered Nurse 1:**

Yeah. We can understand, yeah. There is not only that, from the facial expression, other than, we know by checking pulse rate, pulse is very high, patient is in pain. BPI, blood pressure strong, BP, BP will be high.

**Registered Nurse 2:**

*Patient name* I remember, three days she was crying and then the..

**Registered Nurse 1:**

That's another thing. Pain we know that, if patient is in pain, if there is any joint pain or something means, and there is, maybe there, there will be some redness, swelling and all. So that, we know that she's ha…she's in pain.

**Registered Nurse 4:**

Sometimes tears in the eyes and all.

**Registered Nurse 2?**

*Patient name* would [cry] straight days continuously, uh, her left shoulder I think was, there's some problem. Dislocation, something. She was crying for three days continuously, no?

**Interviewer:**

Oh, bless her. Were you, were you all there, when this happened?

**Registered Nurse 1:**

That day, I told you, no. She's not, uh, she's crying because of the pain. I think you are there?

**Assistant Nurse 1:**

Because if they have pain, they will cry.

**Interviewer:**

Okay. The routine for all, all patients, regardless of their condition, is this the same?

**Registered Nurse 2:**

Mm. Same.

**Interviewer:**

Wake up time, bath time.. okay.

**Interviewer:**

Is there a time you make accomodations, that's..

**Assistant Nurse 1:**

Same means for bedridden patients, it's the same, but for others, no.

**Registered Nurse 1:**

These conscious patients and all, maybe they have some preferences, like they want breakfast at this time, lunch, this time.. yeah, they have preferences, conscious patients.

**Interviewer:**

Okay, and they are accommodated. Yeah, to what extent do we accommodate patients that are bedridden, and that are not verbal? for example, let's say Halima on a Monday morning doesn't feel like taking a bath. Is there a way to understand?...There's no way.

**Assistant Nurse 1:**

No, they cannot. That is out of their…we are providing their…the care, the service.

**Registered Nurse 2:**

That we cannot. That patient, no?

**Interviewer:**

There is no way. Okay. Was there ever a time…

Registered Nurse 4:

*Patient name* when, when she came, she was not taking bath actually. So, after that we convinced her, nowadays, she knows that it's her routine, so she will not, uh, she will come with us. Now, before, my god, we, uh, weekly once we'll help carry, and with all members, they are coming and holding and…

**Registered Nurse1:**

Much difficulty we are giving bath. Haha.

**Interviewer:**

With the male nurses we talked about training, level of knowledge. Uh, did you all take part in the survey I gave back in the summer? The survey, long time ago, yeah? In that survey, we tried to assess the knowledge level, the awareness level of the staff. Um, and I also asked this to the male nurses. As part of your qualifications to become nurses, do they give you specific training on how to care for dementia patients? Is there, like, a portion of your training that's about--

**Registered Nurse 1:**

Actually, we attended so many trainings, like, training means CME classes and all. They contain medical education classes, about dementia, Alzheimer’s, and then, any adaptable geriatric course. Now also we are all attending geriatric course. Ten years back, we have one geriatric course. In that they are explaining all the ten geriatric cases and everything.

**Interviewer:**

Okay. Is it, is it too medical? Or is, is there a psychological component you would say, like for example, engaging with the patient emotionally, if they have an emotional need. For example, *patient name*, if she's…missing her sons, *Nurse name* says she cries. On how, how to engage with them at the emotional level. Is…does, does the training include that, or is it just medical care?

**Registered Nurse 2:**

Mm…both.

**Registered Nurse 1:**

Medical care, and psychological supporting for patients. But if conscious and or another, mostly it has to be go by social workers, right? Yeah.

**Interviewer:**

Okay um, but as part of your registered nursing training, dementia is a topic, a special topic that you cover. Is it not?

**Registered Nurse 2:**

No.

**Interviewer:**

When you started your employment here, was there training given? For patients with dementia.

**Registered Nurse 2:**

N...no actually.

**Interviewer:**

Not really? When, when the- a patient is first, like, admitted, do the doctors communicate with you about their condition in detail, or you have to refer to the notes and figure it out?

**Assistant Nurse 1:**

No, doctors fill in.

**Interviewer:**

Doctors tell? That this patient has dementia?

**Registered Nurse 1:**

Because, same staff and doctor, first will go and see. That time we are with the doctor, so we are communicating. We came to know the patient deeply.

**Interviewer:**

If there was ever any training to be given to you, what do you think it should have? What would be the most useful type of training for you to care for patients with dementia. Given the challenges that you have said; feeding time is challenging. Someone mentioned, um, you know, carrying them, positioning is very- cause some of them can be quite heavy. So, putting all these into consideration-

**Registered Nurse 2:**

Actually these patients, contracture patients, right? So that's why maybe positioning them. That's why she told you.

**Interviewer:**

And also physically, they can be quite heavy as well. For two nurses, it's hard.

**Registered Nurse 1:**

Sometimes, there are not only the nurses. For dementia patients, Doctor part, nurse, physiotherapy, dietician. Everybody, together.

**Interviewer:**

They're part of a team, right. But if there was a training, what should it have in your opinion for it to be impactful, for it to be useful. What kind of training would you benefit from?

**Registered Nurse1:**

For me, I feel like getting them medication, or initial medication and uh, triple then slowly they will move to some strain. Just it, I think everything is okay.

**Interviewer:**

If, do you think patients like *patient name* and *patient name*, if we knew what was going on in their minds, that we would offer them better care? If there was a way to understand what they're thinking.

**Assistant Nurse 1:**

Actually, we are only looking for better care. They are, uh yeah, conscious also, they will be very happy. While receiving eeing, uh, care.

**Interviewer:**

That's the thing. The patients who can communicate like *patient name*, like *patient name* also if you engage with him, I know you don't work with *patient name* directly but he can, he can actually engage with you.

**Registered Nurse 2:**

But..yeah, actually it is difficult to understand for the patient who is bedridden and who is not talking. Their exact needs, because, also..

**Interviewer:**

That's what I mean, that's what I mean. It's much easier with patients that can communicate their needs. If they feel happy or sad, they tell you. But for patients like *patient name* and *patient name*, does it mean that because they have stopped talking, that we just provide the routine care? What if, for example, *patient name* is missing her son? I've heard that he doesn't visit her as often anymore. Wouldn't it be good if we knew that, if you, we knew when she's missing him so that maybe we can call him and tell him to visit? Do you -

**Registered Nurse 2:**

But how?

**Interviewer:**

Ah, how. That’s why we need research. People are continuously doing research trying to get to that. Better ways of delivering care to those living with dementia.

**Registered Nurse 1:**

How will you know?

**Interviewer:**

I don’t. I’m just saying. If there was a way, would you, do you think you would benefit from it?

**Registered Nurse 2:**

That's. Yeah. Better. That would be great, yeah.

**Assistant Nurse 1:**

That would be great actually. If they come and visit. Weekly once, or something. In the beginning itself they are, if they are advising them, like every week, at least one day, come and visit and stay with them for ten minutes, they will be happy, but not talking also.. By, means by holding her hands or something, maybe telling something. We don't know whether they are listening, they can hear. We don't know actually. So let them talk, even if not talking, they are not communicating back also, still. At least, you know. It would be better I think.

**Registered Nurse 1:**

*patient name*, by eye movement we can understand so many things. she we will not be staying, not like *patient name*. *patient name* will still. This, uh, *patient name* will look like this if you are doing something, look like this.

**Interviewer:**

She has her eye gaze, yeah.

**Registered Nurse 1:**

That means she knows what we're doing, only she's not talking but she can understand everything.

**Registered Nurse 2:**

But what you told is correct. If you know the emotional needs of the patient, it will be a great achievement and they will be satisfied also, so that is.. but how? That is the question.

**Interviewer:**

If I had that magic of how, would you be willing to get training from me let's say for example. Would you be happy to do that training? If I were to teach you how to talk to, or communicate with patients like *patient name* and *patient name*. Would you, do you think that would be beneficial?

**Registered Nurse 2:**

It is.

**Interviewer:**

Or there is no point?

**Registered Nurse 2:**

There is.

**Assistant Nurse 1:**

There is.

**Registered Nurse 1:**

I think it's good.

**Interviewer:**

Okay. Any specific topics, specific skills that you think you're, you didn't get from your qualification. Are there any specific skills that you, you think a new training would give you?

**Registered Nurse2:**

Um, it's..

**Registered Nurse 1:**

Yeah, this only, how we can know the emotional feelings of these patients. It's very difficult to know, especially at this stage.

**Registered Nurse 2:**

It's not only for the demen- demented patients. It's whoever is becoming above 70, there should, there should be some. Somebody should be take care of their emotional needs. Somebody has to take them outside, because, you know.

**Interviewer:**

I understand you. So the care that you are giving as qualified nurses, it's called biomedical care. We're psychologists, my students are studying psychology. My, my background is in psychology. So we approach an illness from the psychosocial point of view because the biomedical is achieved by people like you, experts like you. So we fill the gap that exists, which is the psychosocial, which is, you know the surroundings, going out for example, and interacting in a social setting, that's the social element of an illness. So, *nurse name*, if I'm hearing you correctly, you're saying "what we're doing here is perfect, because we're providing the biomedical care, but there has to be more done in terms of meeting their emotional needs." I- Is that what I'm understanding?

**Registered Nurse 2:**

Yes.

**Interviewer:**

And who do you think can do that? Who do you think can provide that care? Is it you by getting more training, or is it staff that's hired specifically for caring for dementia patients. What would that look like? That improvement.

**Registered Nurse 2:**

Since, uh, in a way, mostly, most of them are Arab speaking, right. So, it is better the social workers if they are going, because they know how to communicate in their language. So if they are going and having communication with them, just asking them what they need and what is their, their day, how is their day, all these things if they are asking, it is. It's much beneficial. Because you know, we all are expatriates, we are not that much familiar about Arabic language, and um they, they, these people are talking only in Arabic, and since we have social workers, and their main responsibility is like this, this is the responsibility, so they can do it very well.

**Interviewer:**

So, you think more of that should be done. You mentioned language, you mentioned culture, which is interesting and it's part of our project as well. In what way, in what ways has language barriers or cultural differences, in what ways do they impact your care, caregiving? Do you face that, do you think that's a challenge? Do you experience it in your caregiving? If so, then how? Give me like examples, when language became a barrier.

**Registered Nurse 1:**

Especially language, that's very important, no. Because some, if, if I know Arabic only means I like a staff, a doctor for communicating because I'm so comfortable to express my feelings, pains and everything to the, that language. So if I'm like if talking in Hindi, English and every, they're not comfortable - they cannot express that feeling clearly, so language is very important.

**Interviewer:**

Was there a time a patient of yours tried to express a need, but because of language or because of the culture you come from, you are not able to understand them?

**Registered Nurse 1:**

Yeah.

**Registered Nurse 2:**

Yeah, there are. And whenever they don't, we are not understanding, we used to call some Arab staff or the social workers, somebody, we will call, we used to call them.

**Registered Nurse 1:**

So many patients.

**Interviewer:**

So many patients.

**Assistant Nurse 1:**

Yeah.

**Interviewer:**

How can that be fixed? What's the solution for, that's a challenge?

**Registered Nurse 1:**

We'll ask the patient's preferences. Patient is concious, we'll ask the patient, where they will tell that "I want Arab speaking staff" so we all put that as in..

**Interviewer:**

Okay. Is there shortage in Arab, Arabic speaking staff?

**Registered Nurse 2:**

No, there is not.

**Interviewer:**

There is enough? Okay.

**Interviewer:**

Was there ever a strategy you used and you found it was so successful that you want to use it again when caring for a dementia patient? Until now, we've talked about challenges. I want to change that and talk about success. Which strategy, as part of your caregiving, you used it, and it was so successful that you want to use again? Was there, can you think back and, and remember a time?

**Registered Nurse 4:**

This other than the medications?

**Interviewer:**

Other than the medications. Caregiving. It could be engagement. *Nurse name*, wasn't it you who told me that, um, was it *patient name*, when she used to talk? I'm confusing the nurses now, who told me this I don't remember. Um, but yeah. You mentioned she doesn't often understand or communicate, but when she does, you told me something. Trying to remember what it is. What is that like? When you were faced with a challenge, and then you, you used a strategy, and suddenly it worked. Can you share something like that, has that ever happened?

**Registered Nurse 4:**

That's what they tell, no, while doing dressing..

**Registered Nurse 2:**

Uh when uh no uh. No, some other patient before, one other patient was there. Some people, some patients no, when we are doing, they will be very much grateful. Their look itself is like, we know they're very much grateful and uh..

**Interviewer:**

They appreciate you. Mm.

**Registered Nurse 2:**

Yeah. Like that everything will be there and, we are caring for some patients.

**Interviewer:**

Now, you all have experience, and you know how to work your way around it, like the emotions and the non-verbal patients, you know how to read their faces. But for me, for example for me, when I came here, it, I couldn't instantly understand their communication as well as the staff could, because you are used to them, and that comes from a lot of experience. It's not necessarily something you get taught, because each case is so unique. So, for someone who's coming, and they don't know how to deal with patients with dementia, or non-verbal patients, what do you think, how do you think they can be trained for, to have the experience that you have? These are not necessarily something that everyone comes trained with.

**Registered Nurse2:**

Initially, the staff who is, coming to care is, they also know all of the conditions, right? So, they may be aware. One, within one or two days, they will come - we, we used to give the orientation, and they will come to know how to adapt, how- yeah. We are not telling them to go and care for the patient, no. So, we are giving orientation for few days or weeks, at least for one month then only they will start. And we do have competencies, and after that only, they will start caring the patients... in case of staff. In case of visitors,

**Interviewer:**

Yeah, yeah. It's different.

**Registered Nurse 2:**

Uh, it's different. Because some- we used to see that some visitors are coming and like middle-aged people, they will come and talk and see and cry. All these things but I don't know, I don't think that they need training for that, correct? Just to come and sit. But we can just tell, yeah, these are demented patients. They cannot know much things, they are not remembering the things, all these things we can tell.

**Interviewer:**

One final question, and *Nurse name* is giving me the sign that it's time to go, so I'll end it. If there was anything the organization can do, in terms of providing resources, um, or improving the facilities? Is there anything that you think at the organizational level, there's something that can be done, what would it be? To improve that the care you're providing to patients.

**Registered Nurse 1:**

Yeah, more recreational activities, they can do.

**Interviewer:**

More recreational activities, okay.

**Registered Nurse 2:**

Actually before, before COVID, there were, social gatherings, but now it's very less.

**Interviewer:**

Any other thoughts, before we end?

**Interviewer:**

Thank you, my questions are done but if there are any more ideas that you want throw, yeah. I would be happy to hear them. I know how super busy you are, you have no idea how much I appreciate this.

**Registered Nurse 4:**

Actually training, some training, it would be better I believe.

**Assistant Nurse 1:**

A little more than that, I think that when the patient comes, if they can take some videos, in the beginning of the patient, you know. Some video clips, and in between, changes. And uh, we don't know whether they can, and leave patient in a, some gathering situation. If we show them, like their old memories, I don't know if everybody will understand, but still, when they come in time, that situation, and at present, like some video clips. If we are keeping, it will be, I don't know. It will go to some, a... As they said, I never went to any places, but still I am telling, they said some gatherings are there outside. So on that situation and all, some clips if you keep, it will be better and we can keep here and one day we can show them. I think that's a good way.

**Interviewer:**

Show them, mm. That's a very good idea, thank you so much for sharing.
